# Oxycodone protects cardiac microvascular endothelial cells against ischemia/reperfusion injury by binding to Sigma-1 Receptor

**DOI:** 10.1080/21655979.2022.2057632

**Published:** 2022-04-12

**Authors:** Meihua Ji, Jing Cheng, Daimin Zhang

**Affiliations:** a Department of Anesthesiology, Henan Provincial People’s Hospital, Zhengzhou, Henan, China; b Department of Anesthesiology of Central China Fuwai Hospital, Central China Fuwai Hospital of Zhengzhou University, Zhengzhou, Henan, China; c Department of Cardiology, Nanjing First Hospital, Nanjing Medical University, Nanjing, Jiangsu, China

**Keywords:** Myocardial ischemia/reperfusion, Oxycodone, inflammation, apoptosis, Sigma-1 Receptor

## Abstract

Endothelial dysfunction is an important mechanism involved in myocardial ischemia-reperfusion (I/R) injury. We aimed to explore the effects of Oxycodone on myocardial I/R injury in vivo and in vitro to reveal its mechanisms related to Sigma-1 Receptor (SIGMAR1). A rat model of I/R-induced myocardial injury was developed. The ischemic area and myocardial histopathological changes after oxycodone addition were evaluated by TTC staining and H&E staining. LDH, CK-MB and cTnI levels were used to assess myocardial function. Then, the endothelial integrity was reflected by the expressions of ZO-1, Claudin-1 and Occludin. Afterward, ELISA, RT-qPCR, western blot and immunofluorescence assays were adopted for the detection of inflammation-related genes. SIGMAR1 expression in myocardial tissues induced by I/R and cardiac microvascular endothelial cells (CMECs) under hypoxic/reoxygenation (H/R) was determined using RT-qPCR and western blotting. Subsequently, after SIGMAR1 silencing or BD1047 addition (a SIGMAR1 antagonist), cell apoptosis and endothelial integrity were analyzed in the presence of Oxycodone in H/R-stimulated CMECs. Results indicated that Oxycodone decreased the ischemic area and improved myocardial function in myocardial I/R injury rat. Oxycodone improved myocardial histopathological injury and elevated endothelial integrity, evidenced by upregulated ZO-1, Claudin-1 and Occludin expressions. Moreover, inflammatory response was alleviated after Oxycodone administration. Molecular docking suggested that SIGMAR1 could directly bind to Oxycodone. Oxycodone elevated SIGMAR1 expression and SIGMAR1 deletion or BD1047 addition attenuated the impacts of Oxycodone on apoptosis and endothelial integrity of CMECs induced by H/R. Collectively, Oxycodone alleviates myocardial I/R injury in vivo and in vitro by binding to SIGMAR1.

## Graphical Abstract

**Figure:**
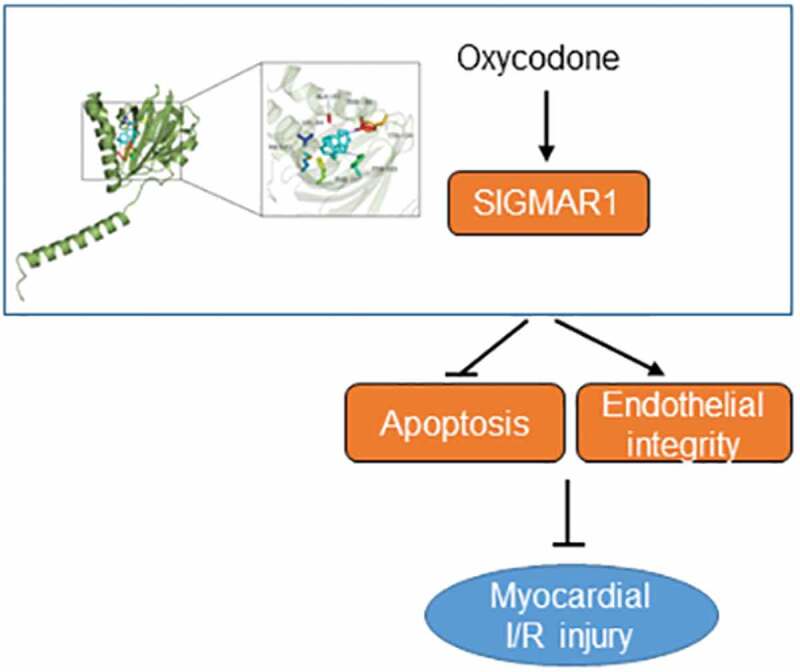

## Introduction

Myocardial ischemia/reperfusion (I/R) injury is a cardiovascular disease accompanied by high morbidity and mortality globally from acute myocardial infarction and coronary artery disease [1]. Timely restoration of blood flow (reperfusion) to the ischemic myocardium is the standard treatment for patients with myocardial ischemia [2]. However, the recovery of perfusion and concomitant reoxygenation exacerbates the dysfunction of myocardium, accelerates cardiomyocyte death and results in structural damage. Despite paramount clinical interests, there is no effective therapy for preventing and treating I/R-induced cardiac injury [3]. Thus, management of I/R injury is important for improving the outcome of these patients.

An increasing number of researches have validated that microvascular disorder is an important mechanism of myocardial I/R injury, and the mechanisms involved include inflammation, calcium overload and pH changes [4,5]. Cardiac microvascular endothelial cells (CMECs) located in the capillaries are the basic components of myocardial microcirculation, which can secrete cytokines related to cardiac growth, contractility and heart rhythm [6]. CMECs participate in up to one-third of the cardiac microvascular system and function to match myocardial perfusion and oxygen consumption [7]. As one of the major cell types damaged immediately after myocardial I/R injury, CMECs dysfunction is considered to be an important pathophysiological event of myocardial I/R injury. Therefore, maintaining microvascular endothelial integrity is a new potential therapeutic approach to improving myocardial I/R injury.

Oxycodone (6-deoxy-7,8-dehydro-14-hydroxy-3-O-methyl-6-oxomorphine) is a multiple-opioid receptor agonist first synthesized in 1917 that commonly serves as a sedative in clinic practice. Accumulating studies have confirmed that oxycodone possesses excellent anti-inflammatory and anti-apoptotic properties. For instance, oxycodone attenuates the acute lung injury-induced rat lung histological changes and reduces pulmonary microvascular permeability via inhibiting inflammation and apoptosis [8]. By downregulating nuclear factor-κB (NF-κB) expression and decreasing tumor necrosis factor-α (TNF-α), interleukin-6 (IL-6) and IL-1β levels in hippocampal astrocytes, Oxycodone protects against lipopolysaccharide-induced neuroinflammatory damage [9]. Oxycodone inhibits oxygen glucose deprivation/recovery-induced hippocampal neuronal apoptosis [10]. In addition, studies have also shown the cardioprotective effects of oxycodone, that is, it can inhibit myocardial I/R injury by hampering apoptosis of cardiomyocytes [11,12]. However, whether oxycodone can reduce the damage of CMECs is still unknown. The swisstarget database (http://www.swisstargetprediction.ch/) showed that oxycodone could target Sigma-1 Receptor (SIGMAR1), a member of a relatively novel receptor group that is a unique binding site commonly expressed in the central nervous system and other peripheral tissues [13]. Emerging evidence supports the notion that activation of SIGMAR1 relieves myocardial apoptosis in rats suffering from myocardial I/R injury [14]. Importantly, compelling evidence indicates that chronic SIGMAR1 activation can improve the ventricular remodeling after myocardial infarction in rats and decrease the susceptibility to ventricular arrhythmia [15], suggesting that SIGMAR1 might relieve myocardial I/R injury. By employing the human protein expression database (https://www.proteinatlas.org/), we observed that SIGMAR1 is widely expressed in myocardial tissues and expressed in CMECs. For this reason, we speculated that Oxycodone could target SIGMAR1 to impart protective effects against CMECs damage during myocardial I/R.

In the present study, the myocardial I/R injury rat model was established to investigate the effects of Oxycodone on the inflammation and microvascular damage. The further experiments were performed to explore the regulation of Oxycodone on SIGMAR1 in CMECs under hypoxic/reoxygenation (H/R) condition. Our findings may open novel avenues for future therapies of myocardial I/R damage.

## Materials and methods

### Animal

A total of 24 male Sprague-Dawley (SD) rats weighing 200–300 g (6–8 weeks age) provided by the Model Animal Research Center of Nanjing University (Nanjing, China) were housed in air-conditioned rooms at 23 ± 2°C with a 12-h light/dark cycle. Rats were given a standard diet and allowed to drink water freely. Before the experiment, the rats were adaptively reared for 1 week. Animal experiment got the permission from the Animal Research Ethics Committee of Nanjing First Hospital, Nanjing Medical University.

### Induction of myocardial I/R injury

Myocardial I/R surgery was conducted according to previous description [16]. To simulate myocardial I/R injury, rats were anesthetized intraperitoneally with 40 mg/kg sodium pentobarbital. After the pericardium being dissected open, the left anterior descending was exposed and occluded by ligation with a 5–0 silk suture. The slipknot was released following 30 min of ischemia and rats received 2 h of reperfusion [17]. The ischemia was confirmed by observing the color change of the ischemic area of myocardial tissue, and reperfusion was achieved by loosening the ligature. Animals were sacrificed through intraperitoneal injection of 200 mg/kg sodium pentobarbital after receiving 2 h of reperfusion. The blood samples and heart tissues were harvested with the aim of performing follow-up experiments.

### Grouping and drug administration

The SD rats were randomly distributed into groups of control, sham, I/R, I/R + Oxycodone (n = 6 in each group). Rats in the Oxycodone treated group were subjected to an intravenous injection of 0.5 mg/kg Oxycodone for 30 min prior to surgery. Rats in the other groups underwent intravenous injection with normal saline at the same time.

### Evaluation of myocardial infarct area

The infarct area of the myocardium was tested by 2,3,5-triphenyltetrazolium chloride (TTC). Following the rinse with normal saline for three times, cardiac tissues were sliced into 2-mm-thick transverse sections for five slices. Then, 1% TTC solution (cat. no. T8877; Sigma-Aldrich) was used to stain cardiac tissues at 37°C for 15 min. The infarct size was identified with the application of Image J software (Version 1.52 r; National Institutes of Health, Bethesda, MA, USA).

### Determination of release of lactate dehydrogenase (LDH), creatine kinase- myocardial band (CK-MB) and cardiac troponin I (cTnI) into serum

To reflect the myocardial cellular damage, blood samples were centrifuged at 3000 rpm for 20 min at 4°C to obtain serum. The levels of LDH (cat. no. A020-1-2), CK-MB (cat. no. H197-1-1) and cTnI (cat. no. H149-2) in serum were evaluated by the corresponding commercial kits supplied by Nanjing Jiancheng Bioengineering Institute (Nanjing, China).

### Hematoxylin and Eosin (H&E) staining

Following the rinse with normal saline for three times, heart tissues were immersed in 4% paraformaldehyde for 24 h and embedded with conventional paraffin wax. 5-μm-thick sections were obtained. Deparaffinization of the sections was performed and the sections were then stained with hematoxylin and eosin, prior to dehydration in a graded ethanol series and xylene. The sections were imaged with the aid of a light microscope (Olympus Corporation).

### Measurement of inflammatory cytokines

The secretion of TNF-α (cat. no. F16960), IL-1β (cat. no. F15810) and IL-6 (cat. no. F15870) in serum were determined with the enzyme-linked immunosorbent assay (ELISA) kits provided by Shanghai Xitang Biotechnology (Shanghai, China) as per the standard procedures of the supplier. The optical density (OD) value was evaluated at 450 nm with the adoption of a microplate reader (Molecular Devices, USA).

### Cell culture

Rat CMECs were obtained from iCell Bioscience (Shanghai, China) and cultured in the medium filling with a mixture of Dulbecco’s Modified Eagle’s Medium (DMEM; HyClone, Logan, UT) and 10% fetal bovine serum (FBS; HyClone, Logan, UT). Cells were kept in culture under 5% CO_2_ at 37°C.

### Hypoxic/reoxygenation (H/R) induction and treatment

To construct the H/R models, CMECs were placed in an anaerobic chamber with the anoxic atmosphere of 94% N_2_, 5% CO_2_ and 1% O_2_ for 30 min to simulate the hypoxic condition. After establishing hypoxia, cells were transferred to a normal cell incubator with a mixture of 95% air and 5% CO_2_ for 2 h [18]. The control group cells without hypoxia treatment were kept in normoxic conditions. 0.5, 1 and 1.5 ng/mL Oxycodone were used to treat CMECs [11]. CMECs were treated in advance with 10 μM BD1047, a SIGMAR1 antagonists, for 30 min before Oxycodone addition [19].

### Cell viability assay

CMECs (3000 cells/well) were inoculated into 96-well plates at 37°C for 24 h. Then, 10 μl cell counting kit-8 (CCK-8) reagent was applied to incubate the cells in each well for another 4 h, and the absorbance at 450 nm was determined by means of a microplate reader (Molecular Devices, USA).

### Cell transfection

To knockdown SIGMAR1, the specific short hairpin RNAs (shRNAs) targeting SIGMAR1 (shRNA-SIGMAR1-1 or shRNA-SIGMAR1-2) which regarded shRNA-NC as the negative control were designed and synthesized by Genechem (Shanghai, China). Later, these vectors were transfected into cells with the application of Lipofectamine 2000 (cat. no. 11,668,030; Invitrogen, Carlsbad, CA, USA). After 48 h of transfection, the transfection efficiency was evaluated by reverse transcription-quantitative (RT-q) PCR and western blotting.

### Immunofluorescence

After different treatment, CMECs were fixed with 4% paraformaldehyde for 30 min, washed with phosphate buffer solution (PBS) for three times, and then treated with 0.1% Triton X-100 (Solarbio, Beijing) at room temperature for 20 min. After being washed, the inhibition of cells by 5% bovine serum albumin (BSA; Solarbio, Beijing) was performed for 30 min at room temperature. Subsequently, cells were carried on incubating overnight with primary antibodies against Zona occludens 1 (ZO-1; cat. no. 20,742-1-AP; Proteintech, Chicago, IL, USA) and Gr-1 (cat. no. bs-23,111 R; Bioss, Beijing China) at 4°C. After that, samples were interacted with anti-rabbit secondary antibody (cat. no. 4412S; Cell Signaling Technology, Boston, MA, USA) at 37°C for 1 h. And the cells were subjected to a light-proof incubation with DAPI for 5 min.

### Terminal-deoxynucleoitidyl transferase mediated nick end labeling (TUNEL) staining

The apoptosis of CMECs was determined by means of a TUNEL staining kit (cat. no. C1088; Beyotime, Shanghai, China) following manufacturer’s recommendations. Briefly, cells were first immobilized in 4% paraformaldehyde and subsequently treated with 0.5% Triton X-100. 50 μl TUNEL was employed to incubate the cells for 1 h at 37°C. Nuclei was stained with the adoption of 4’,6-diamidino-2-phenylindole (DAPI) solution. The apoptotic-positive cells were indicated by green staining. Cells from five randomly selected fields of view were imaged under a fluorescence microscope (Olympus Corporation).

### RT-qPCR assay

After the isolation of total RNA from hearts tissues and cells with the aid of TRIzol Reagent (Invitrogen, Carlsbad, CA, USA), complementary DNA (cDNA) was synthesized by reverse transcription of RNA using a RevertAid RT Kit (cat. no. K1691; Invitrogen, Carlsbad, CA, USA). PCR was undertaken with the use of PrimeScript™ RT-PCR Kit (cat. no. RR014B; TaKaRa) on an Applied Biosystems (ABI) PCR System 7500 (ABI; Foster City, CA, USA). Primers used in this experiments were as follow: ZO-1, forward 5’-ACAGTACAGCCAGCCAGTTC-3’, reverse 5’-GGCTCAGCAGAGTTTCACCT-3’; Claudin-1, forward 5’-TGGGGCTGATCGCAATCTTT-3’, reverse 5’-ACTTAAGGAGCACCCTTCGC-3’; Occludin, forward 5’-ATTGAGCCCGAGTGGAAAGG-3’, reverse 5’-GAGGTAGCACCACGTTGGAA-3’; TNF-α, forward 5’-AACACACGGACGCTGAAGT-3’, reverse 5’-TCCAGTGAGTTCCGAAAGCC-3’; IL-1β, forward 5’-CCTTGTCGAGAATGGGCAGT-3’, reverse 5’-CAGGGAGGGAAACACACGTT-3’; IL-6, forward 5’-CACTTCACAAGTCGGAGGCT-3’, reverse 5’-AGCACACTAGGTTTGCCGAG-3’; monocyte chemoattractant protein 1 (MCP-1), forward 5’-TAGCATCCACGTGCTGTCTC-3’, reverse 5’-CAGCCGACTCATTGGGATCA-3’; SIGMAR1, forward 5’-ATGGCCATTCGGGACGATAC-3’, reverse 5’-TCATTGCTCCCCAAGAGCTG-3’; Glyceraldehyde-phosphate dehydrogenase (GAPDH), forward 5’-TGTGAACGGATTTGGCCGTA-3’, reverse 5’-GATGGTGATGGGTTTCCCGT-3’. Quantification was undertaken in accordance with a comparative Ct (2^−ΔΔCT^) method [20]. GAPDH was designated as a standard internal control for relative gene expression.

### Western blot analysis

Total proteins were isolated from cardiac tissues and cultured cells utilizing ice-cold RIPA buffer (Beyotime, Shanghai, China), followed by the identification of protein concentrations with the bicinchoninic acid (BCA) Protein Assay kit (cat. no. P0012S; Beyotime, Shanghai, China) in line with the instructions of the supplier. Following protein electrophoresis separation applying sodium dodecyl sulfate-polyacrylamide gel electrophoresis (SDS-PAGE) gels, these proteins were moved onto polyvinylidene fluoride (PVDF) membranes which were blocked for 2 h. Subsequently, appropriate primary antibodies were employed to incubate the membranes at 4^°^C overnight. The primary antibodies used in this study included anti-ZO-1 (cat. no. 20,742-1-AP; Proteintech, Chicago, IL, USA), anti-Claudin-1 (cat. no. 28,674-1-AP; Proteintech, Chicago, IL, USA), anti-Occludin (cat. no. 91131S; Cell Signaling Technology, Boston, MA, USA), anti-TNF-α (cat. no. 17,590-1-AP; Proteintech, Chicago, IL, USA), anti-IL-1β (cat. no. ab254360; Abcam, Cambridge, UK), anti-IL-6 (cat. no. sc-57,315; Santa Cruz Biotechnology, CA, USA), anti-phospho (p)-NF-κB p65 (cat. no. 3033 T; Cell Signaling Technology, Boston, MA, USA), NF-κB p65 (cat. no. 8242 T; Cell Signaling Technology, Boston, MA, USA), p-inhibitor of kappa B-α (IκB-α) (cat. no. 2859 T; Cell Signaling Technology, Boston, MA, USA), anti-IκB-α (cat. no. 4812S; Cell Signaling Technology, Boston, MA, USA), anti-MCP-1 (cat. no. 25,542-1-AP; Proteintech, Chicago, IL, USA), anti-SIGMAR1 (cat. no. 61994S; Cell Signaling Technology, Boston, MA, USA), anti-B-cell lymphoma 2 (Bcl-2) (cat. no. 60,178-1-Ig; Proteintech, Chicago, IL, USA), anti-Bcl-2-associated X protein (Bax) (cat. no. 2772 T; Cell Signaling Technology, Boston, MA, USA), anti-cleaved caspase3 (cat. no. 9661 T; Cell Signaling Technology, Boston, MA, USA), anti-caspase3 (cat. no. 9662S; Cell Signaling Technology, Boston, MA, USA) and anti-GAPDH (5174 T, Cell Signaling Technology, Boston, MA, USA). Then, these blots were incubated by horseradish peroxidase (HRP)-conjugated secondary antibodies (cat. no. 7074S and cat. no. 7076S; Cell Signaling Technology, Boston, MA, USA) at room temperature for 1 h. After washing with PBS, an Ultra High Sensitivity Enhanced Chemiluminescence kit (cat. no. HY-K1005; MedChemExpress) was prepared and adopted to detect protein bands. Finally, the blotting film was placed in a luminescent imager for photography with Image J software (Version 1.52 r; National Institutes of Health, Bethesda, MA, USA).

### Molecular docking

The interaction of Oxycodone and SIGMAR1 was predicted by molecular docking method. The structures of Oxycodone and SIGMAR1 were acquired from the PDB database (https://www.rcsb.org/) and constructed by the Molecular Operating Environment software. Subsequently, the 3D structures of Oxycodone and SIGMAR1 were matched. And the 32-bit command program CMD.exe was used to calculate the affinity between Oxycodone and SIGMAR1.

### Statistical analysis

The measured data were presented in the form of mean ± standard deviation. Comparisons among multiple groups were done by One-way analysis of variance (ANOVA) as well as the corresponding Tukey post-hoc test. All data processing was done on GraphPad Prism software 8.0 (La Jolla, CA, USA). The variability among groups was regarded as statistically significant at P-value <0.05.

## Results

### Oxycodone decreases the ischemic area and improves myocardial function in rat with myocardial I/R injury

**Figure 1.**
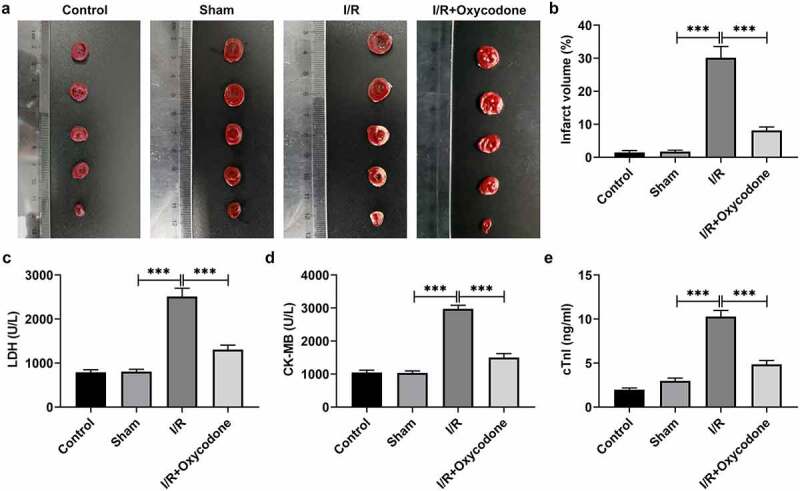
Oxycodone decreased the ischemic area and improved myocardial function in rat with myocardial I/R injury. (a) The myocardial ischemic area was identified through TTC staining. (b) Percentage of myocardial infarct volume. The levels of (c) LDH, (d) CK-MB and (e) cTnl in serum were evaluated with commercial kits. ***P < 0.001.

Several previous studies have demonstrated the cardioprotective effects of oxycodone on cardiomyocytes under I/R condition by hampering apoptosis [11,12]. However, whether oxycodone could reduce the damage of CMECs during I/R was still unknown. Firstly, the effects of Oxycodone on the myocardial ischemic area were detected by TTC staining. As presented in Figure 1a-b, the myocardial infarct size in the sham group had no significant difference (vs Control). A significant increase in ischemic area was observed after I/R induction as comparison to the sham group, which was prominently reduced following the further addition of Oxycodone. Additionally, to reflect the myocardial cellular damage, the levels of LDH, CK-MB and cTnl in serum of rats in each group were tested using commercial kits. It could be found that I/R exposure led to notably elevated LDH, CK-MB and cTnl levels when compared to the sham group (Figure 1c-e). However, Oxycodone treatment inhibited the increase in LDH, CK-MB and cTnl levels induced by I/R. These results implied a protective role of Oxycodone in myocardial I/R injury.

### Oxycodone ameliorates myocardial histopathological injury and enhances endothelial integrity in rat with myocardial I/R injury

**Figure 2.**
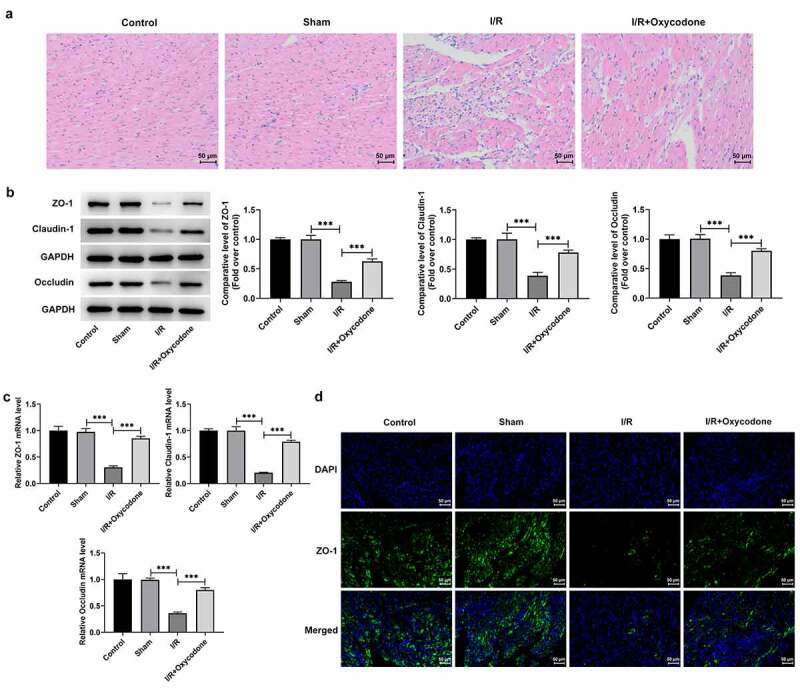
Oxycodone ameliorated myocardial histopathological injury and enhanced endothelial integrity in rat with myocardial I/R injury. (a) The myocardial histopathological changes were measured by H&E staining. The (b) protein and (c) mRNA expressions of ZO-1, Claudin-1 and Occludin in myocardial tissues were examined by western blot analysis and RT-qPCR, respectively. (d) Immunofluorescent staining was applied for the determination of ZO-1 expression. ***P < 0.001.

Then, H&E staining was performed to evaluate the effects of Oxycodone on the pathological changes of myocardial tissues in each group. As displayed in Figure 2a, the myocardia in the sham group were arranged regularly and showed no significant changes relative to the control group. By contrast, in the I/R group, the myocardia were arranged disorganized and ruptured, with inflammatory cells in the interstitial tissues. Particularly, Oxycodone administration distinctly ameliorated the myocardial histopathological injury induced by I/R stimulation. Results from Figure 2b-c revealed that I/R stimulation caused conspicuously downregulated protein and mRNA expressions of tight junction proteins such as ZO-1, Claudin-1 and Occludin, in myocardial tissues in comparison with the sham group, which were partially reversed by Oxycodone treatment. Meanwhile, immunofluorescence results indicated that I/R-induced decrease in ZO-1 level was markedly elevated by Oxycodone (Figure 2d). These findings suggested that Oxycodone could attenuate myocardial histopathological injury and enhance endothelial integrity in rat with myocardial I/R injury.

### Oxycodone attenuates inflammatory response in myocardial tissues of rat with myocardial I/R injury

**Figure 3.**
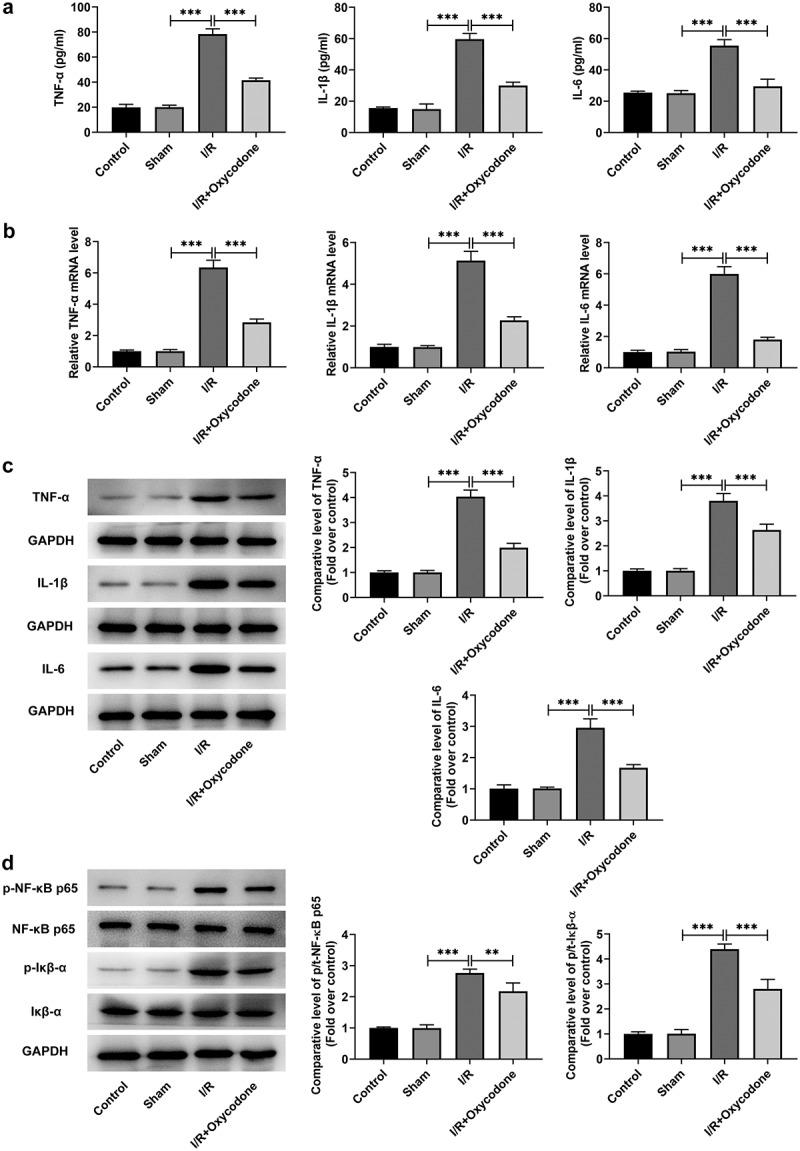
Oxycodone attenuated inflammatory response in myocardial tissues of rat with myocardial I/R injury. (a) The releases of TNF-α, IL-1β and IL-6 in rat serum were detected utilizing ELISA. The (b) mRNA and (c) protein expressions of TNF-α, IL-1β and IL-6 were examined with RT-qPCR and western blotting. (d) Analysis of p-NF-κB p65 and p-IκB-α proteins was conducted using western blot assay. **P < 0.01, ***P < 0.001.

**Figure 4.**
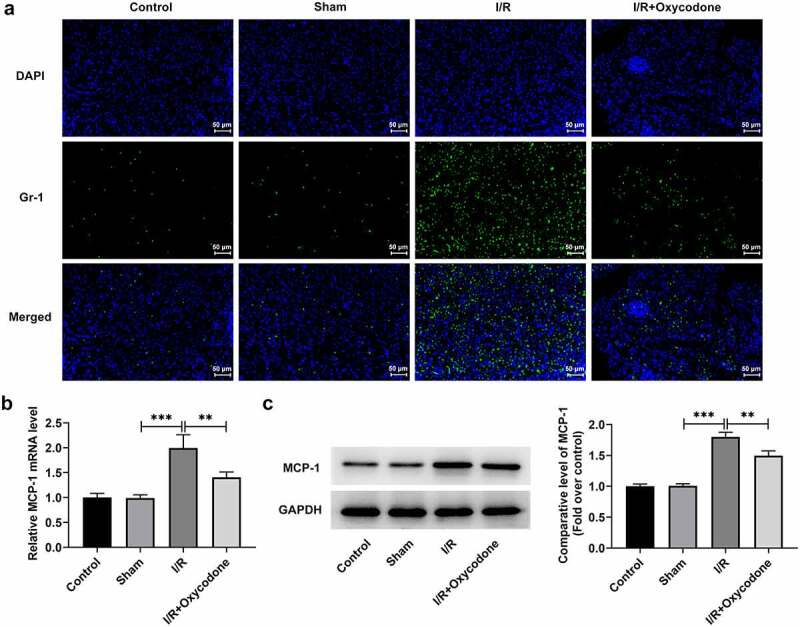
Oxycodone mitigated inflammatory response in myocardial tissues of rat with myocardial I/R injury. (a) Detection of Gr-1 expression was carried out through immunofluorescent staining. The (b) mRNA and (c) protein expressions of MCP-1 were assessed with RT-qPCR and western blot analysis. **P < 0.01, ***P < 0.001.

To study the effects of Oxycodone on the inflammation of myocardial tissues during myocardial I/R injury, the levels of TNF-α, IL-1β and IL-6, in serum and heart tissues were detected with ELISA and RT-qPCR. As what is observable from Figure 3a-b, no significant difference was found in the levels of TNF-α, IL-1β and IL-6 between sham and control groups. On the contrary, I/R exposure dramatically increased the secretion of these inflammatory factors as comparison to the sham group, whereas the addition of Oxycodone alleviated the impacts of I/R induction on TNF-α, IL-1β and IL-6 levels. Concurrently, the protein levels of TNF-α, IL-1β and IL-6 tested by western blot analysis presented the same results as ELISA and RT-qPCR assay (Figure 3c). Besides, it could be found in Figure 3d that the expression levels of p/t-NF-κB p65 and p/t-IκB-α were obviously elevated in the I/R group compared with the sham group, which were restored by Oxycodone addition. Moreover, the expressions of Gr-1 and MCP-1 was evaluated. As exhibited in Figure 4a-c, i/R stimulation notably enhanced Gr-1 and MCP-1 expressions, which was partially counteracted by Oxycodone. These observations revealed that Oxycodone ameliorated inflammatory response in myocardial tissues of rat with myocardial I/R injury.

### Oxycodone activates SIGMAR1 expression in I/R-induced rat myocardial tissues and H/R-induced CMECs

**Figure 5.**
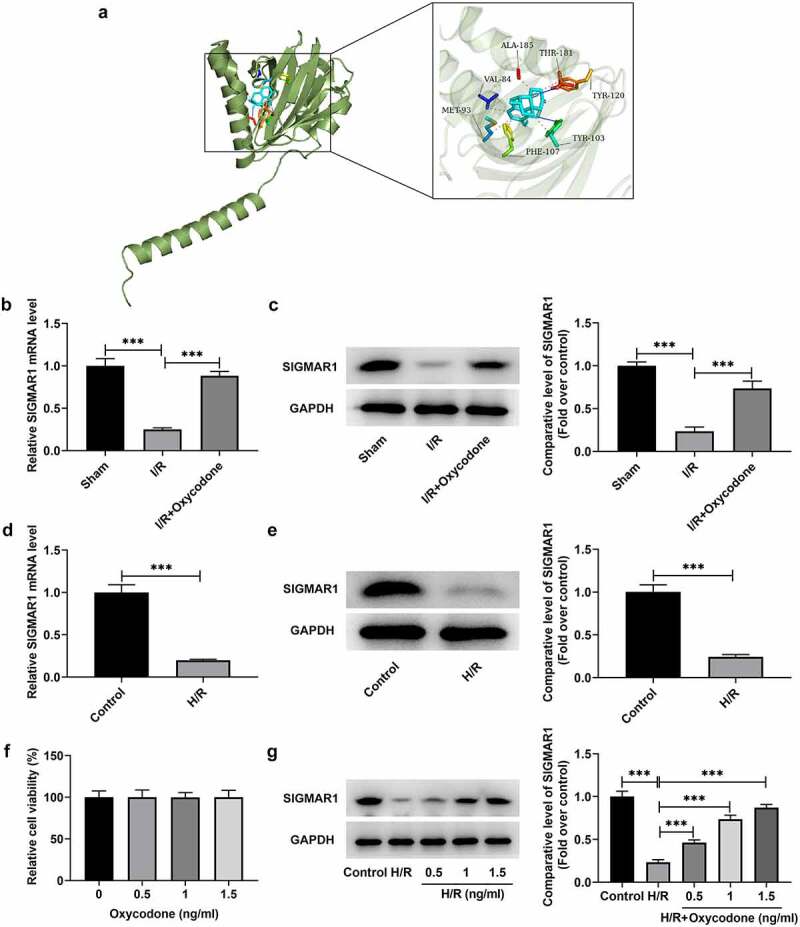
Oxycodone could target and upregulate SIGMAR1 expression in I/R-induced rat myocardial tissues and H/R-induced CMECs. (a) The molecular docking result between Oxycodone and SIGMAR1. The (b) mRNA and (c) protein expressions of SIGMAR1 in I/R-induced rat myocardial tissues were assessed using RT-qPCR and western blotting. The (d) mRNA and (e) protein expression levels of SIGMAR1 were detected by RT-qPCR and western blotting. (f) Cell viability was tested by a CCK-8 assay after CMECs being treated with different concentrations of Oxycodone. (g) SIGMAR1 expression was tested with the application of western blot analysis in H/R-induced CMECs with Oxycodone treatment. ***P < 0.001.

To explore the potential mechanisms of Oxycodone in the protective effects of myocardial I/R injury, Swisstarget database was used to predict the molecule that could be targeted by Oxycodone. SIGMAR1 was found to be a potential protein that could be combined with Oxycodone, which was confirmed by the result of molecular docking experiments (Figure 5a). Then, it was illustrated in Figure 5b-c that SIGMAR1 expression was notably decreased in the I/R group relative to the sham group. On the contrary, the further Oxycodone addition increased SIGMAR1 level when compared to the I/R group. In vitro, H/R treatment also upregulated SIGMAR1 expression when compared to the control group (Figure 5d-e). Afterward, it was found that 0.5, 1 and 1.5 ng/ml Oxycodone had no obvious impacts on the viability of CMECs (figure 5f). Furthermore, under the H/R condition, CMECs exhibited low SIGMAR1 expression as comparison to the control group (Figure 5g). However, Oxycodone dose-dependently elevated SIGMAR1 expression in H/R-induced CMECs. Through the above findings, we proved that Oxycodone could target and upregulate SIGMAR1 expression in I/R-induced rat myocardial tissues and H/R-induced CMECs.

### Oxycodone reduces apoptosis of H/R-induced CMECs by upregulating SIGMAR1 expression

**Figure 6.**
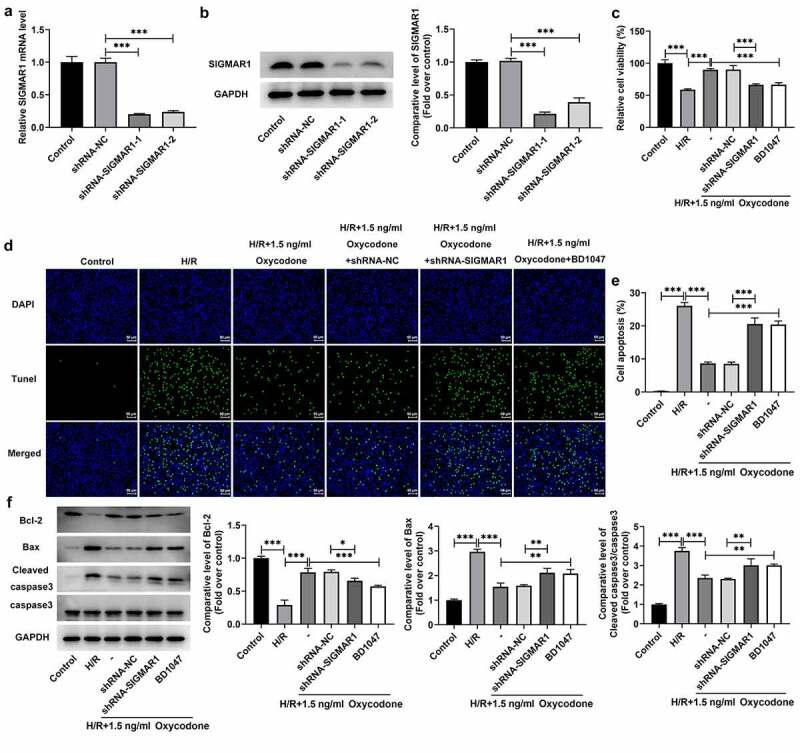
Oxycodone reduced apoptosis of H/R-induced CMECs by upregulating SIGMAR1 expression. The (a) mRNA and (b) protein expressions of SIGMAR1 were examined with the use of RT-qPCR and western blotting. (c) Assessment of cell viability employed CCK-8. (d-e) Cell apoptosis estimation was undertaken utilizing TUNEL. (f) Western blot analysis was adopted for the evaluation of apoptosis-related proteins. *P < 0.05, **P < 0.01, ***P < 0.001.

To further explore whether the impacts of Oxycodone on I/R-induced myocardial damage were realized by regulating SIGMAR1 expression, SIGMAR1 was silenced in CMECs by transfection with shRNAs targeting SIGMAR1. As displayed in Figure 6a-b, SIGMAR1 expression was conspicuously downregulated in the shRNA-SIGMAR1-1 and shRNA-SIGMAR1-2 groups compared with the shRNA-NC group. CMECs transfected with shRNA-SIGMAR1-1 were selected to conduct subsequent experiments due to its better transfection efficiency. Then, as what is observable from Figure 6c, h/R stimulation-induced loss of CMECs viability, which was then increased after Oxycodone addition. By contrast, the further SIGMAR1 deletion reduced cell viability when compared to the H/R + 1.5 ng/ml Oxycodone+shRNA-NC group. The addition of BD1047, a SIGMAR1 antagonist, exhibited the same effects on cell viability as shRNA-SIGMAR1 transfection. Afterward, results obtained from Figure 6d-e indicated that H/R-induced increase in apoptosis of CMECs was significantly decreased by Oxycodone treatment. On the contrary, SIGMAR1 silencing and BD1047 intervention markedly elevated cell apoptosis. Meanwhile, a drop in the expression of Bcl-2 was observed in H/R-stimulated CMECs, accompanied by upregulated Bax and cleaved caspase3 expression, which was reversed by Oxycodone treatment (figure 6f). However, SIGMAR1 silencing and BD1047 intervention attenuated the impacts of Oxycodone on the expression of aforementioned apoptosis-related proteins. These data evidenced that Oxycodone inhibited apoptosis of H/R-induced CMECs by upregulating SIGMAR1 expression.

### Oxycodone enhances endothelial integrity of H/R-induced CMECs by upregulating SIGMAR1 expression

**Figure 7.**
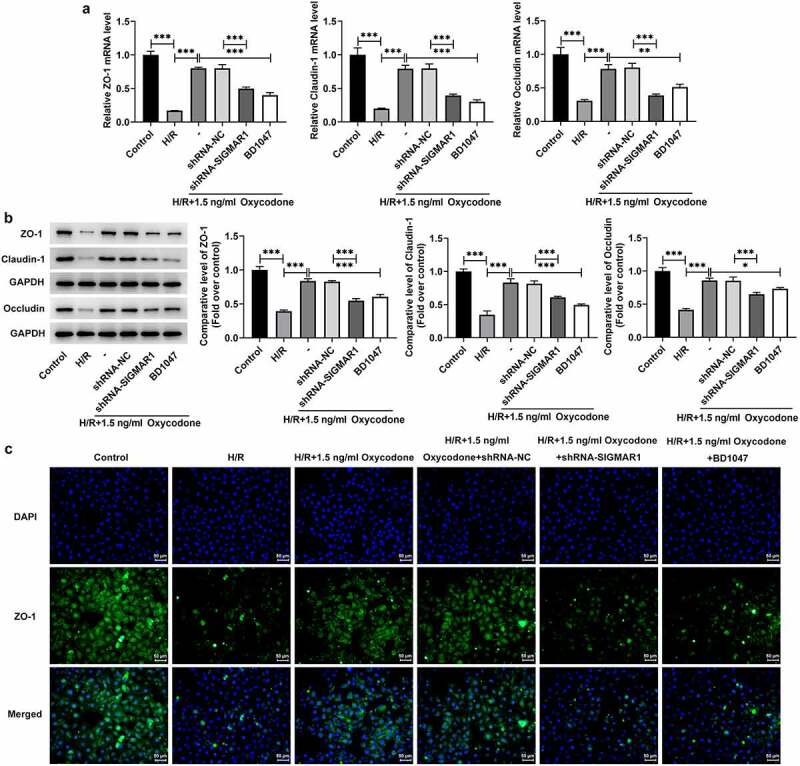
Oxycodone could enhance endothelial integrity of H/R-induced CMECs by upregulating SIGMAR1 expression. The (a) mRNA and (b) protein expression levels of ZO-1, Claudin-1 and Occludin were examined employing RT-qPCR and western blot analysis, respectively. (d) Immunofluorescent staining was applied for the identification of ZO-1 expression. *P < 0.05, **P < 0.01, ***P < 0.001.

Disruption of endothelial integrity is a common pathological process following myocardial I/R, which promotes neutrophil infiltration, inflammation and ultimately impairs myocardial function [21]. Then, endothelial integrity was evaluated in H/R-induced CMECs with Oxycodone treatment. It was found that H/R stimulation downregulated ZO-1, Claudin-1 and Occludin expressions at transcriptional level and post-transcriptional level relative to the control group, which were restored by Oxycodone addition (Figure 7a-b). However, SIGMAR1 knockdown and BD1047 application alleviated the impacts of Oxycodone on ZO-1, Claudin-1 and Occludin expressions in H/R-induced CMECs. Consistently, immunofluorescent staining showed the same results on ZO-1 as RT-qPCR and western blotting (Figure 7c). Together, these findings suggested that Oxycodone could enhance endothelial integrity of H/R-induced CMECs by upregulating SIGMAR1 expression.

## Discussion

Myocardial infarction is a common and serious clinical disease and a threat to public health worldwide in the future and tissue damage caused by myocardial ischemia is an important cause of fatal diseases [22,23]. In the present study, the protective effects of Oxycodone on myocardial I/R injury were investigated in vivo and in vitro. Results demonstrated that Oxycodone decreased the ischemic area, improved myocardial function, ameliorated myocardial histopathological injury and enhanced endothelial integrity in rat with myocardial I/R injury. The further in vitro experiments suggested that Oxycodone alleviated myocardial I/R injury by binding to SIGMAR1.

Multiple mechanisms contribute to the progression of myocardial I/R injury. Among them, endothelial damage is a key mediator of myocardial I/R injury. Endothelial cells make up the endocardial epithelium and the inner layer of myocardial capillaries, which are much more sensitive to I/R in the heart than cardiomyocytes [24,25]. Baars et al. have highlighted that amelioration of endothelial injury can improve long-term outcomes in patients and animals suffering from myocardial infarction [26]. A previous study has shown that I/R induction can upregulate the expression of surface adhesion molecules on endothelial cells and recruit neutrophils, which further damages the endothelial cells [21]. Disruption of endothelial integrity is a common pathological process following myocardial I/R, which promotes neutrophil infiltration, inflammation and ultimately impairs myocardial function. Endothelial integrity depends on the intercellular junction complex between adjacent endothelial cells, and ZO-1 has been shown to be one of the key components in this complex [27]. Occludin and Claudin-1 are two important transmembrane tight junction proteins [28]. Although distributed mainly in the nervous system, opioid receptors have also been proven to be expressed in endothelial cells [29]. Oxycodone has been demonstrated to ameliorate permeability damage of pulmonary microvascular endothelial cells in acute lung injury-induced rat model via inhibiting inflammation and apoptosis [8]. Shao et al. held the opinion that oxycodone treatment notably improved the permeability damage by elevating the expression of ZO-1 and occludin in oxygen-glucose deprivation/reoxygenation-induced brain microvascular endothelial cells [30]. It is noteworthy that the cardioprotective effects of Oxycodone are reflected in its ability to prevent myocardial damage from I/R by suppressing apoptosis of cardiomyocytes [11,12]. This study was the first to explore the effects of Oxycodone on endothelial integrity in rat with myocardial I/R injury. We found that Oxycodone greatly upregulated ZO-1, Occludin and Claudin-1 expressions in rat myocardial tissues during myocardial I/R injury, suggesting that Oxycodone could effectively improve myocardial endothelial cell integrity.

Myocardial inflammation has been recognized as a key hallmark of myocardial I/R injury [31–33]. Compelling evidence indicate that a large number of inflammatory mediators and chemokines are produced during myocardial I/R injury, and leukocytes, platelets and vascular endothelial cells are activated to express a large number of adhesion molecules, which promote the adhesion of leukocytes to vascular endothelial cells [34]. Furthermore, I/R increases endothelial permeability by disrupting endothelial barrier function, which will exacerbate myocardial inflammation after I/R stimulation [35]. TNF-α, IL-1β, and IL-6 are important inflammatory factors that play crucial roles in cell damage during myocardial I/R [36,37]. Additionally, the accumulation of neutrophils in the myocardium is a key event in myocardial I/R injury. In the early inflammatory response after acute myocardial injury, neutrophils dominate the infiltrating immune cell population and cause the greatest tissue damage [38]. Gr-1 and MCP-1 are important markers to reflect neutrophil infiltration in myocardial I/R injury [39]. Myocardial I/R damage exhibits a distinct relationship with cell apoptosis [40]. A previous study has suggested that in isolated perfused hearts subjected to I/R, endothelial apoptosis occurs earlier than it does in cardiomyocytes [41]. Therefore, strategies aimed at suppressing the inflammatory response and apoptosis favor amelioration of myocardial I/R injury. In recent years, an increasing number of researches have focused on the anti-inflammatory and anti-apoptotic effects of Oxycodone. For instance, by inhibiting NF-κB signaling, Oxycodone inhibited lipopolysaccharide-induced neuroinflammation in hippocampal astrocytes [9]. Kong et al. demonstrated that Oxycodone hampered oxygen-glucose deprivation/recovery-induced hippocampal neurons apoptosis in rats [10]. Oxycodone attenuated pulmonary microvascular permeability via inhibiting inflammation and apoptosis [8]. Similarly, the present study revealed that Oxycodone treatment relieved the inflammation and apoptosis, evidenced by the decreased levels of TNF-α, IL-1β, IL-6, p-NF-κB p65, p-IκB-α, Gr-1, MCP-1, Bax, cleaved caspase3 and increased level of Bcl-2. These results demonstrated the inhibitory effects of Oxycodone on inflammation and apoptosis in I/R-challenged rat myocardial tissues and H/R-induced CMECs.

To study the mechanism of Oxycodone action on myocardial I/R injury, the possible targets were predicated by using swisstarget database and SIGMAR1 was noticed as a potential target of Oxycodone, which was further confirmed by the result of molecular docking analysis. As a molecular chaperone, SIGMAR1 possesses multiple cellular functions and is implicated in various diseases. For example, the activation of SIGMAR1 alleviated the release of inflammatory cytokines by retinal Müller glial cells [42]. Accumulating study suggested that the induction of SIGMAR1 expression exhibited outstanding neuroprotective effects in Parkinson’s disease and Alzheimer’s disease [43–45]. Additionally, overexpression of SIGMAR1 has been demonstrated to inhibit cell apoptosis previous in vivo and in vitro studies [46,47]. Importantly, SIGMAR1, a molecular chaperone protein commonly expressed in the body, is also widely expressed in myocardial tissues and in CMECs [48]. SIGMAR1 loss of function results in the impairment of mitochondrial function and adverse cardiac remodeling, ultimately leading to cardiac contractile dysfunction [49]. It has been well reported that activation of SIGMAR1 relieved myocardial apoptosis in rats suffering from myocardial I/R injury [14]. Particularly, chronic SIGMAR1 activation can improve the ventricular remodeling after myocardial infarction in rats and decrease the susceptibility to ventricular arrhythmia [15], suggesting that SIGMAR1 might help to alleviate myocardial I/R injury. In this study, the in vivo and in vitro experiments supported that Oxycodone conspicuously upregulated SIGMAR1 expression after I/R or H/R stimulation. The rescue experiments indicated that SIGMAR1 silencing or SIGMAR1 antagonists BD1047 intervention alleviated the protective effects of Oxycodone on H/R-induced damage of CMECs, suggesting that Oxycodone attenuated myocardial I/R injury by binding to SIGMAR1.

Of course, our experiment has a limitation. In this study, we only discussed the regulatory effect of Oxycodone on SIGMAR1 by silencing SIGMAR1 or adding SIGMAR1 antagonist in H/R-induced CMECs. The further in vivo experiments involved in transgenic animals will be performed in the future investigation to support the conclusion obtained in this study.

## Conclusion

Collectively, this study is the first evidence confirming the protective effects of Oxycodone on myocardial I/R injury from the perspective of cardiac microvascular endothelial cells. We demonstrated that Oxycodone can alleviate myocardial I/R damage in both I/R-exposed rats and H/R-induced CMECs by binding to SIGMAR1 and upregulating SIGMAR1 expression. Our results might provide a rationale to use Oxycodone, a multiple-opioid receptor agonist, as a new therapy to treat myocardial I/R injury via targeting SIGMAR1, suggesting that Oxycodone may be a promising complementary agent for clinically treating ischemic heart disease.

## Supplementary Material

Supplemental Material

## Data Availability

All data present in the paper is available for reasonable request to the corresponding author.
