# Supplementary figures and images for: Oxycodone protects cardiac microvascular endothelial cells against ischemia/reperfusion injury by binding to Sigma-1 Receptor (part 2 of 2)
Source: Bioengineered. 2022 Apr 12;13(4):9628–44. doi: 10.1080/21655979.2022.2057632 (PMC9161947; doi:10.1080/21655979.2022.2057632)

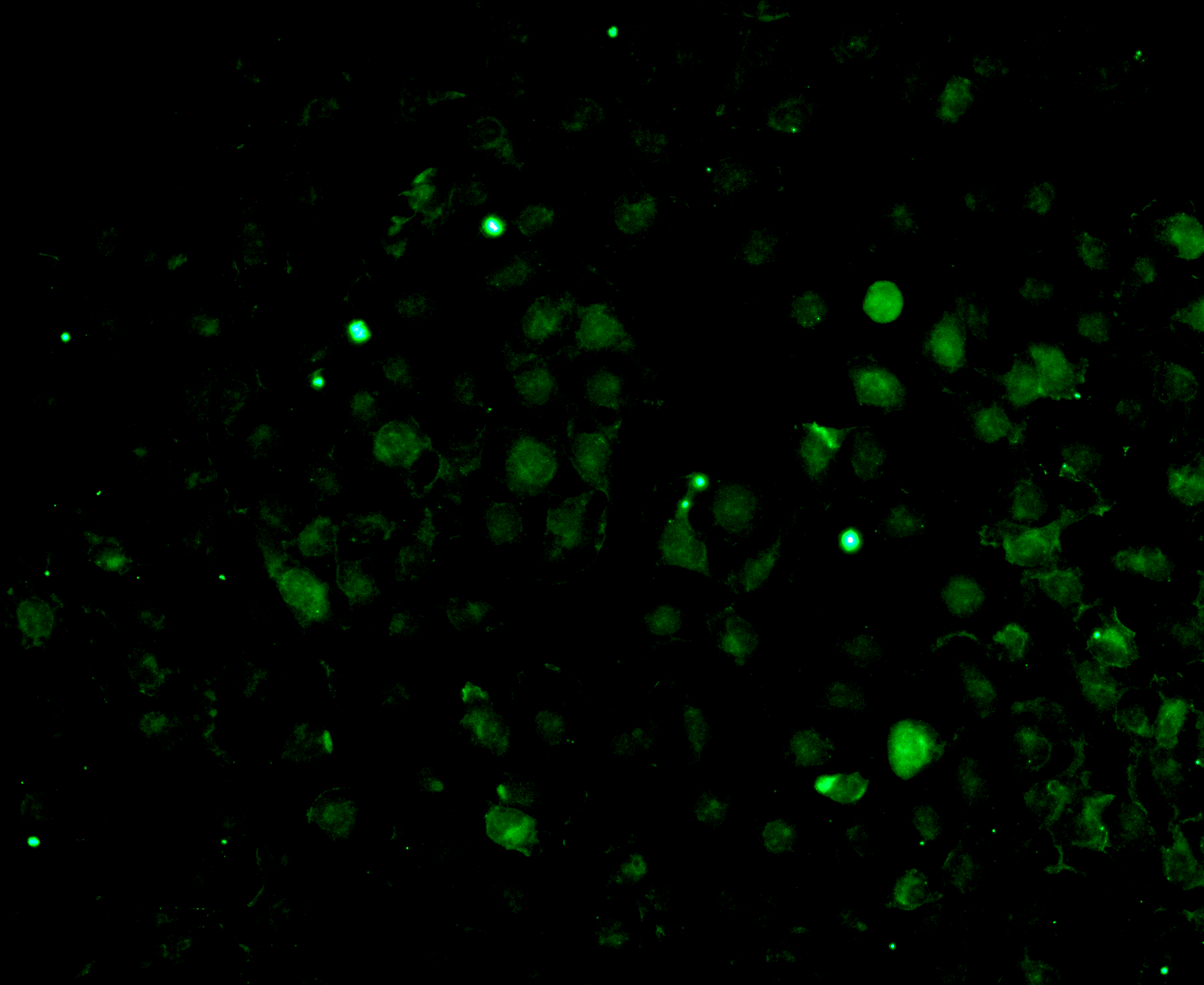

Supplement: Supplemental Material [file KBIE_A_2057632_SM9317.zip › supplementary/Fig7C_HR_1_5 ngmL_Oxycodone_shRNA_SIGMAR1_ZO_1.tif]

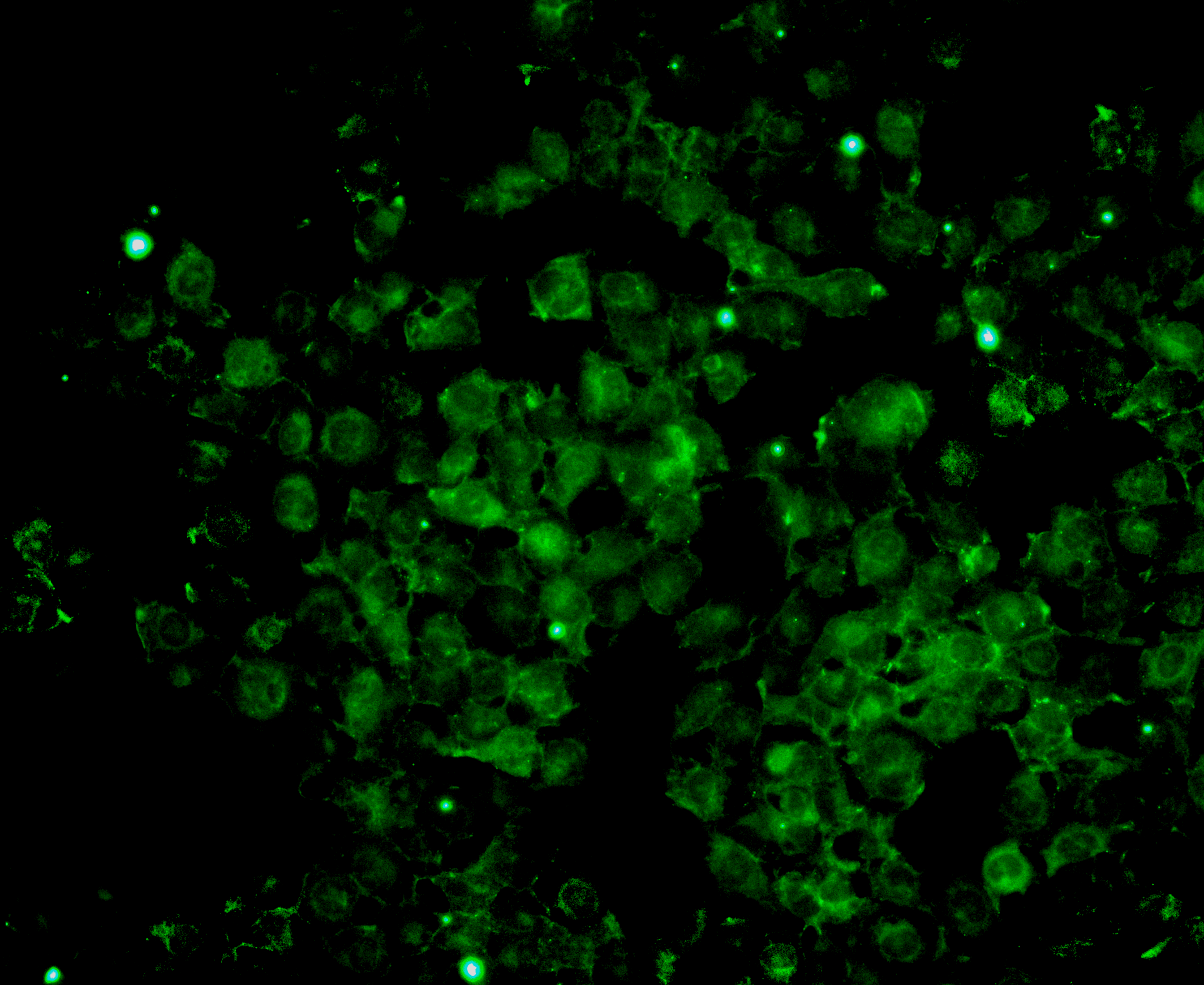

Supplement: Supplemental Material [file KBIE_A_2057632_SM9317.zip › supplementary/Fig7C_HR_1_5 ngmL_Oxycodone_ZO_1.tif]

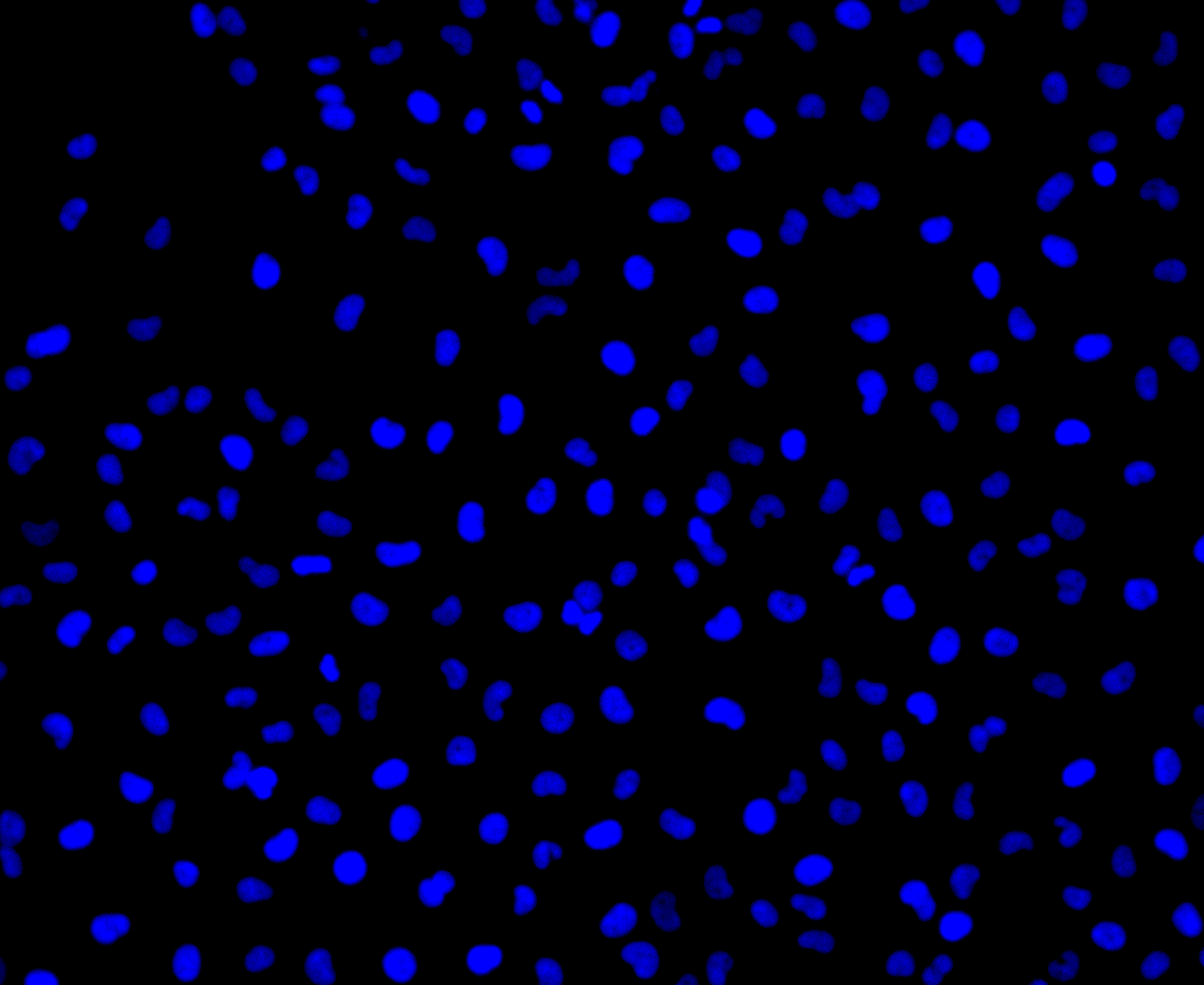

Supplement: Supplemental Material [file KBIE_A_2057632_SM9317.zip › supplementary/Fig7C_HR_DAPI.tif]

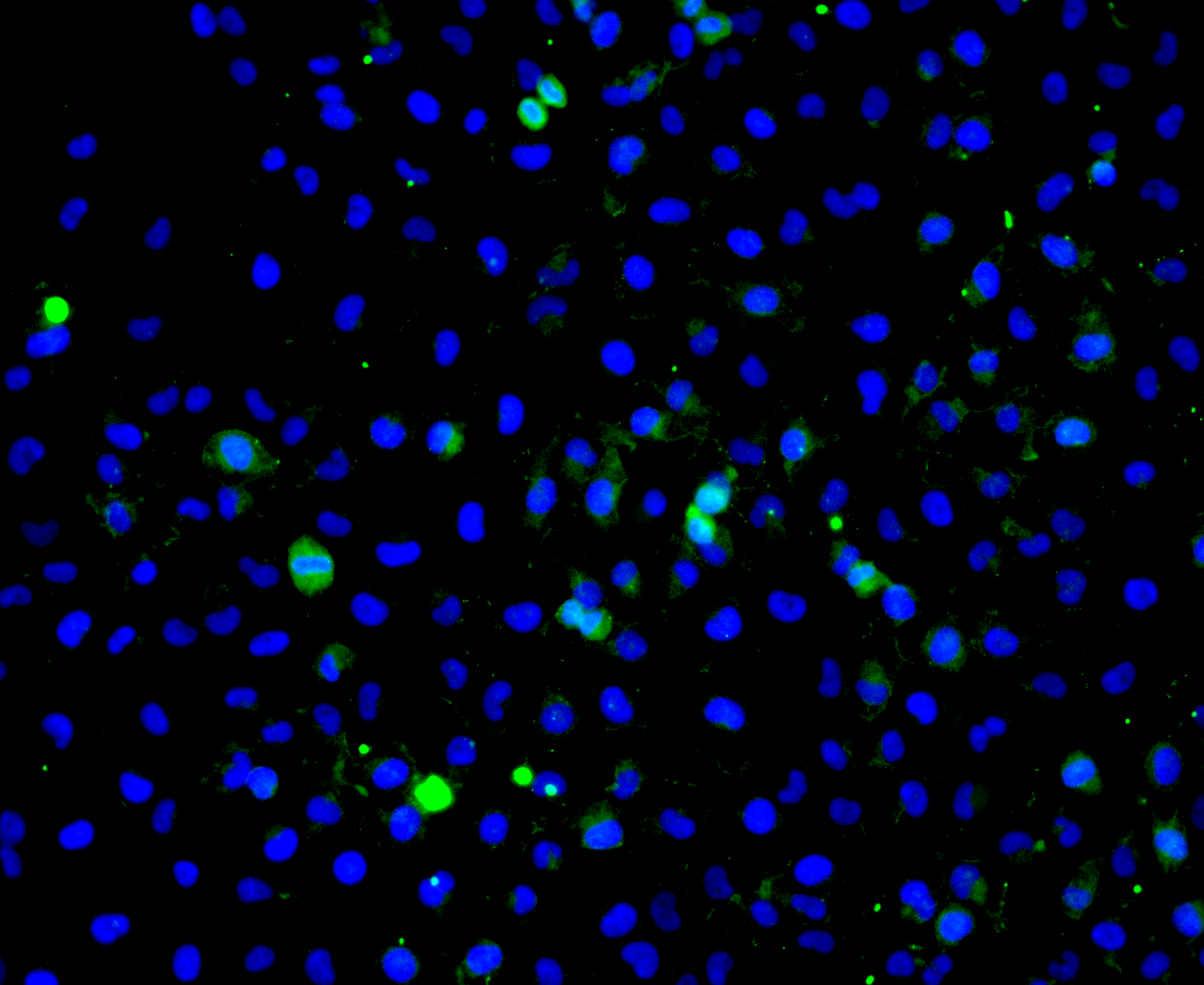

Supplement: Supplemental Material [file KBIE_A_2057632_SM9317.zip › supplementary/Fig7C_HR_Merged.tif]

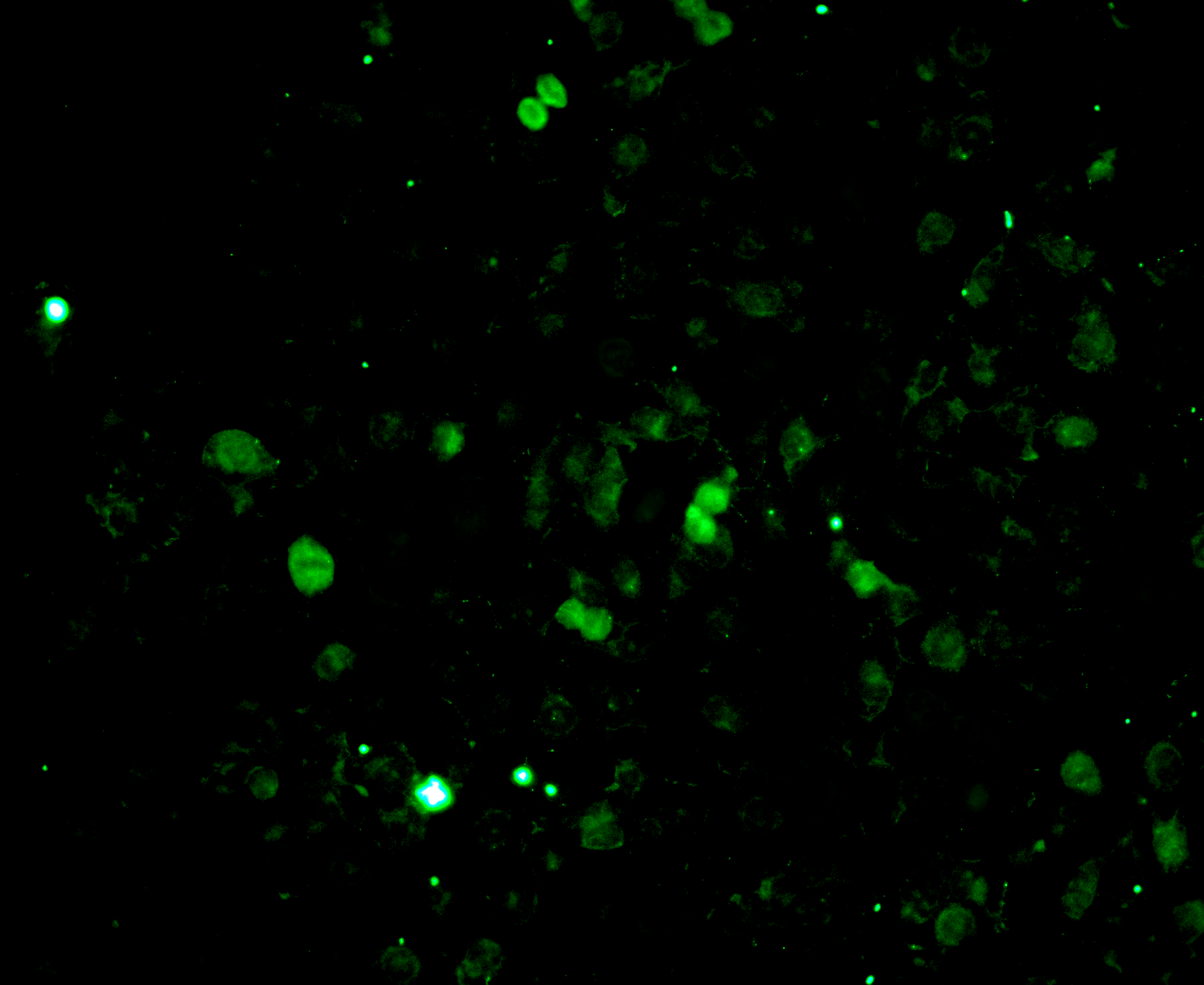

Supplement: Supplemental Material [file KBIE_A_2057632_SM9317.zip › supplementary/Fig7C_HR_ZO_1.tif]

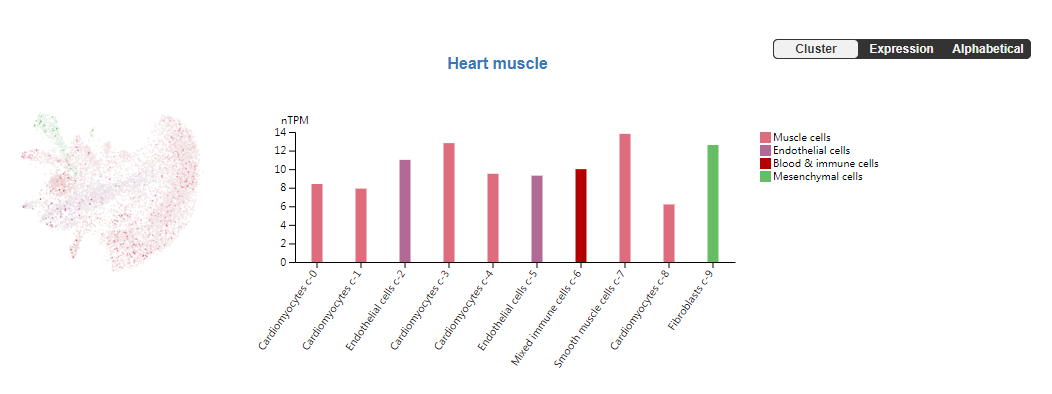

Supplement: Supplemental Material [file KBIE_A_2057632_SM9317.zip › supplementary/human protein expression database_SIGMAR1.png]
