# Supplementary figures and images for: Oxycodone protects cardiac microvascular endothelial cells against ischemia/reperfusion injury by binding to Sigma-1 Receptor (part 1 of 2)
Source: Bioengineered. 2022 Apr 12;13(4):9628–44. doi: 10.1080/21655979.2022.2057632 (PMC9161947; doi:10.1080/21655979.2022.2057632)

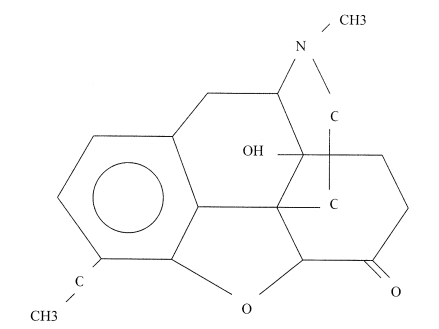

Supplement: Supplemental Material [file KBIE_A_2057632_SM9317.zip › supplementary/Chemical structure of oxycodone.png]

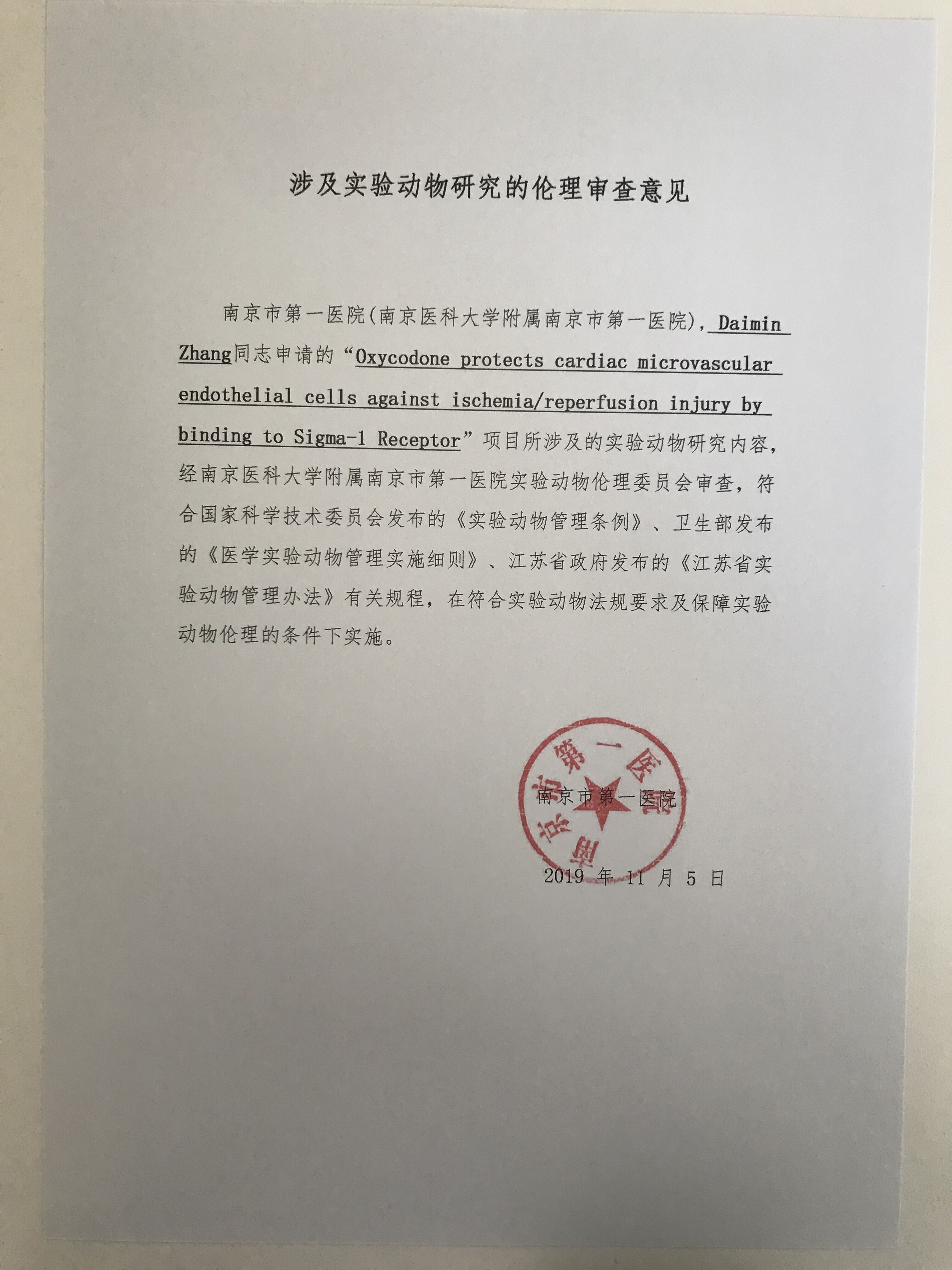

Supplement: Supplemental Material [file KBIE_A_2057632_SM9317.zip › supplementary/Ethical approvement.jpg]

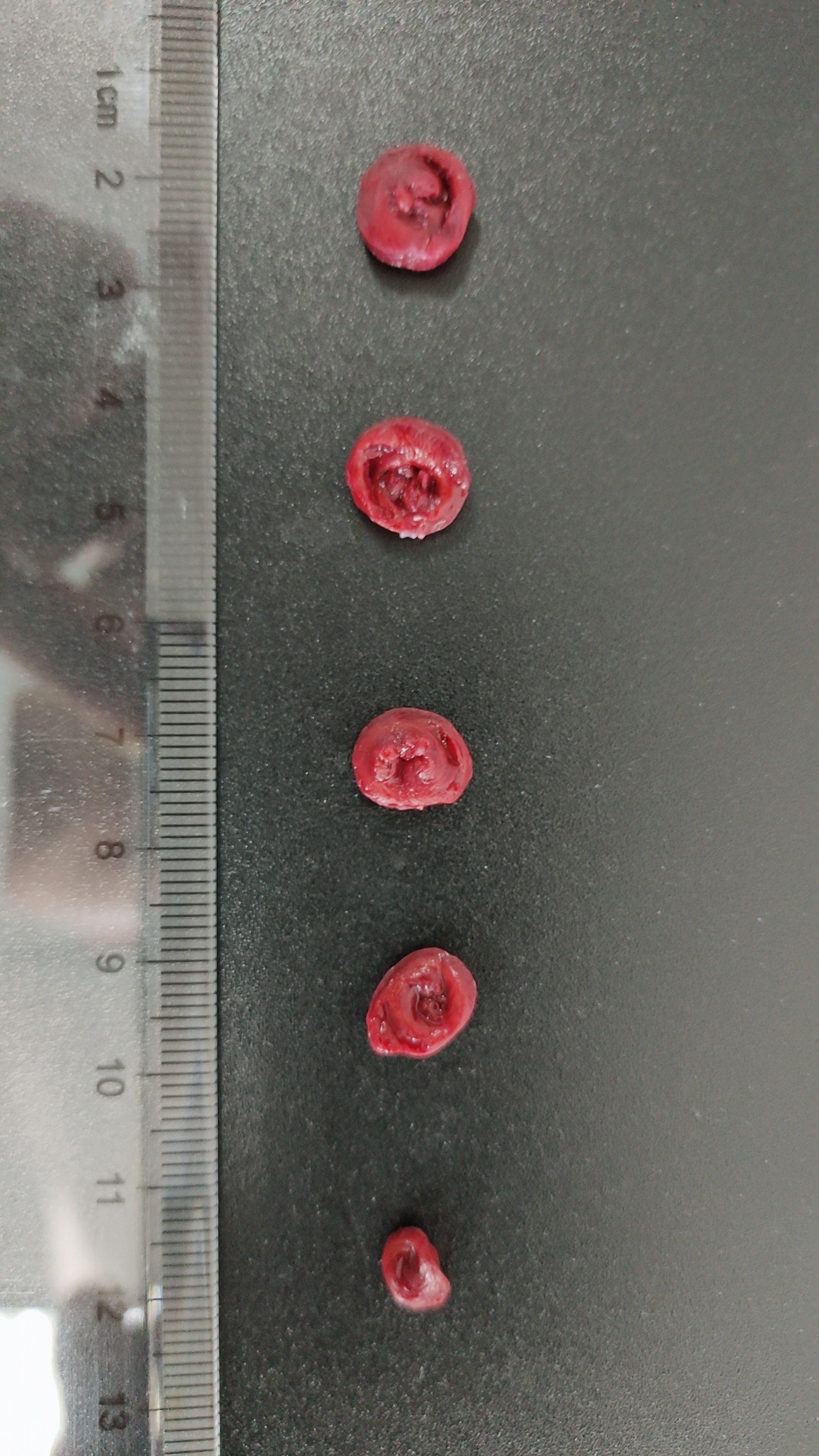

Supplement: Supplemental Material [file KBIE_A_2057632_SM9317.zip › supplementary/Fig1A_Control.jpg]

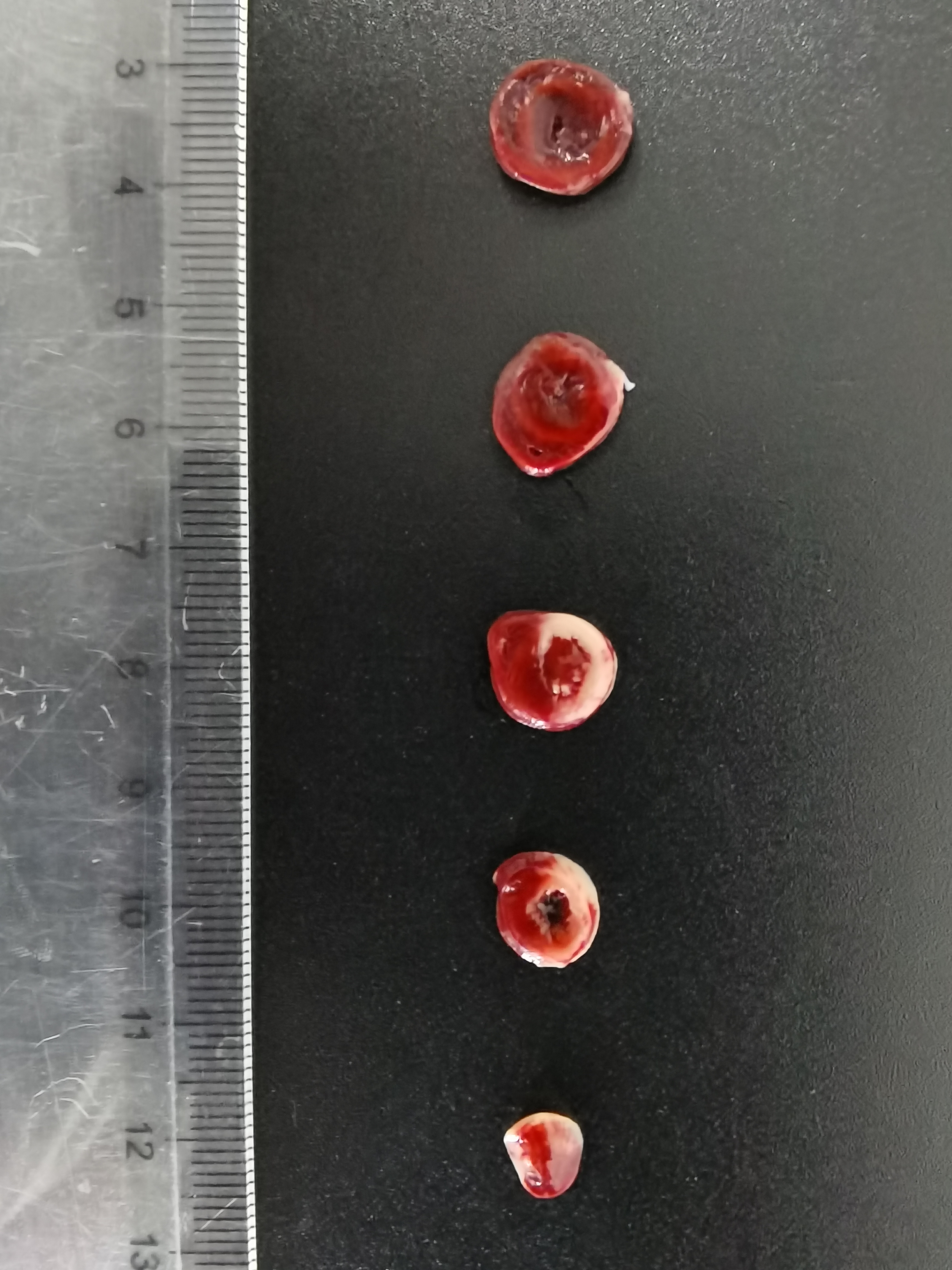

Supplement: Supplemental Material [file KBIE_A_2057632_SM9317.zip › supplementary/Fig1A_IR.jpg]

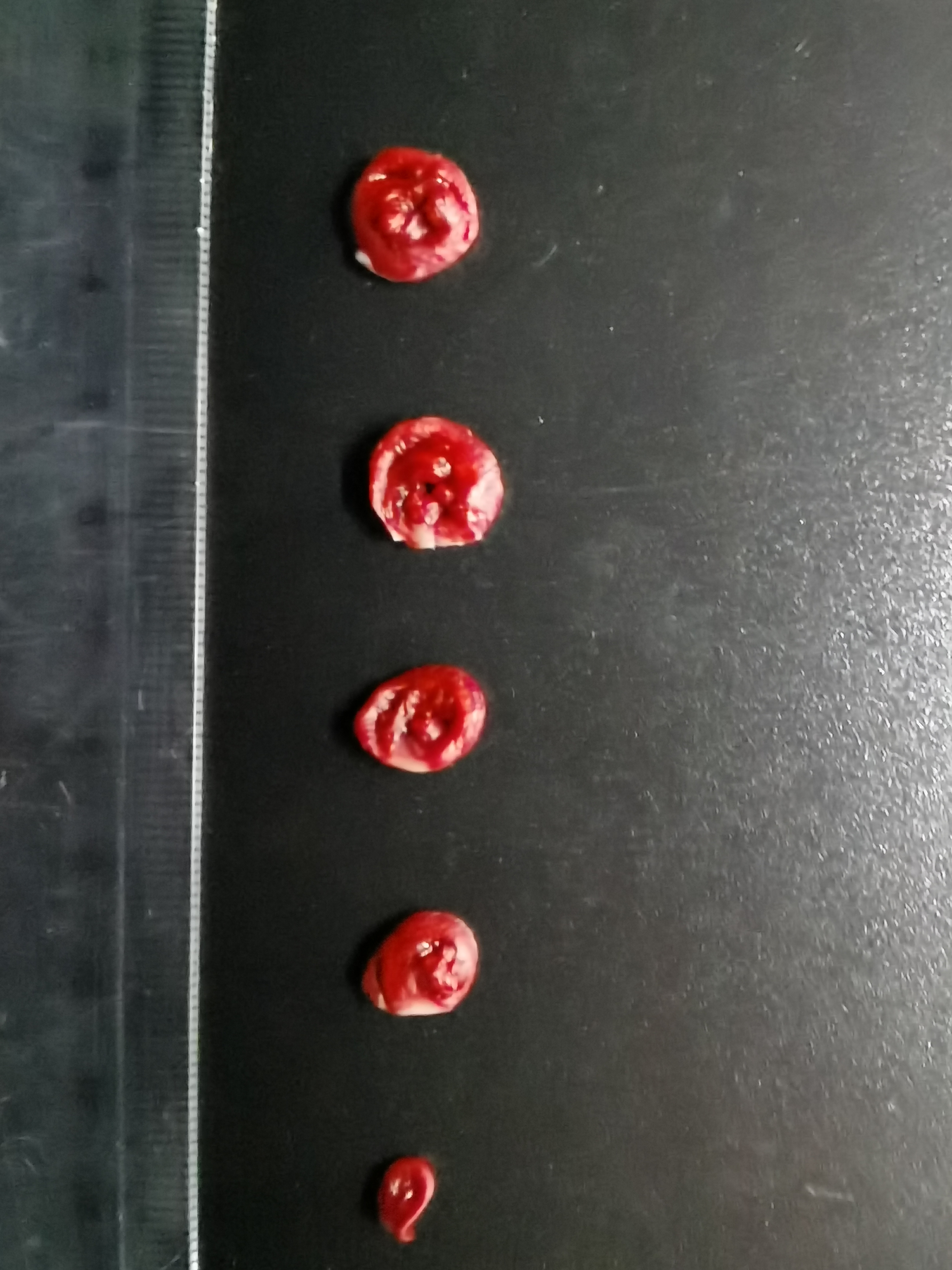

Supplement: Supplemental Material [file KBIE_A_2057632_SM9317.zip › supplementary/Fig1A_IR_Oxycodone.jpg]

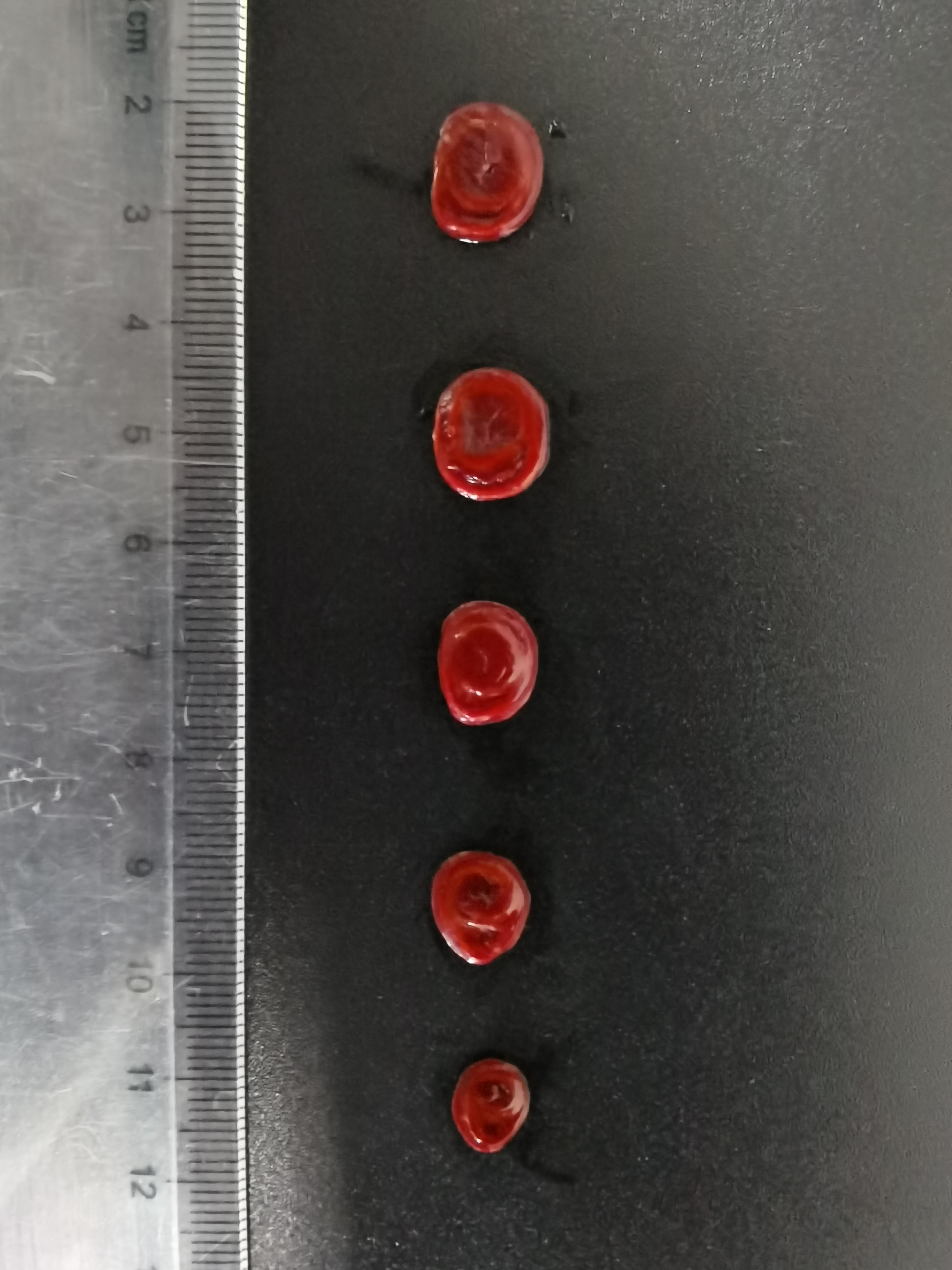

Supplement: Supplemental Material [file KBIE_A_2057632_SM9317.zip › supplementary/Fig1A_Sham.jpg]

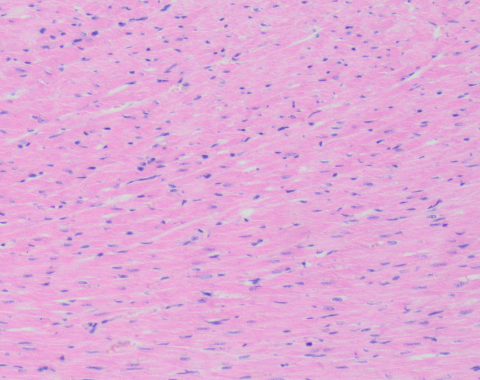

Supplement: Supplemental Material [file KBIE_A_2057632_SM9317.zip › supplementary/Fig2A_Control.png]

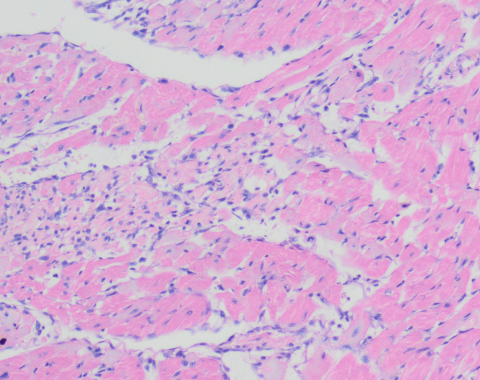

Supplement: Supplemental Material [file KBIE_A_2057632_SM9317.zip › supplementary/Fig2A_IR.png]

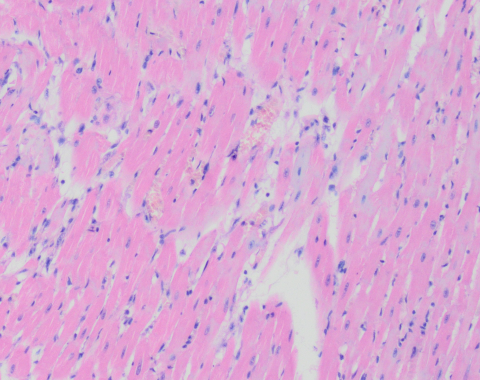

Supplement: Supplemental Material [file KBIE_A_2057632_SM9317.zip › supplementary/Fig2A_IR_Oxycodone.png]

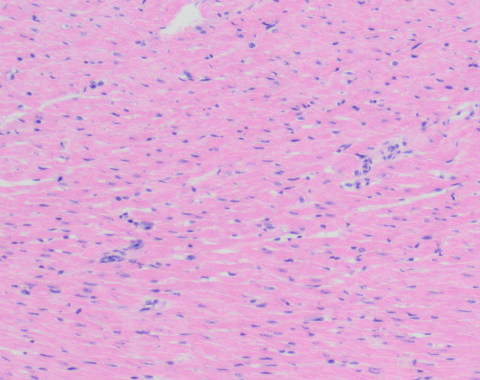

Supplement: Supplemental Material [file KBIE_A_2057632_SM9317.zip › supplementary/Fig2A_Sham.png]

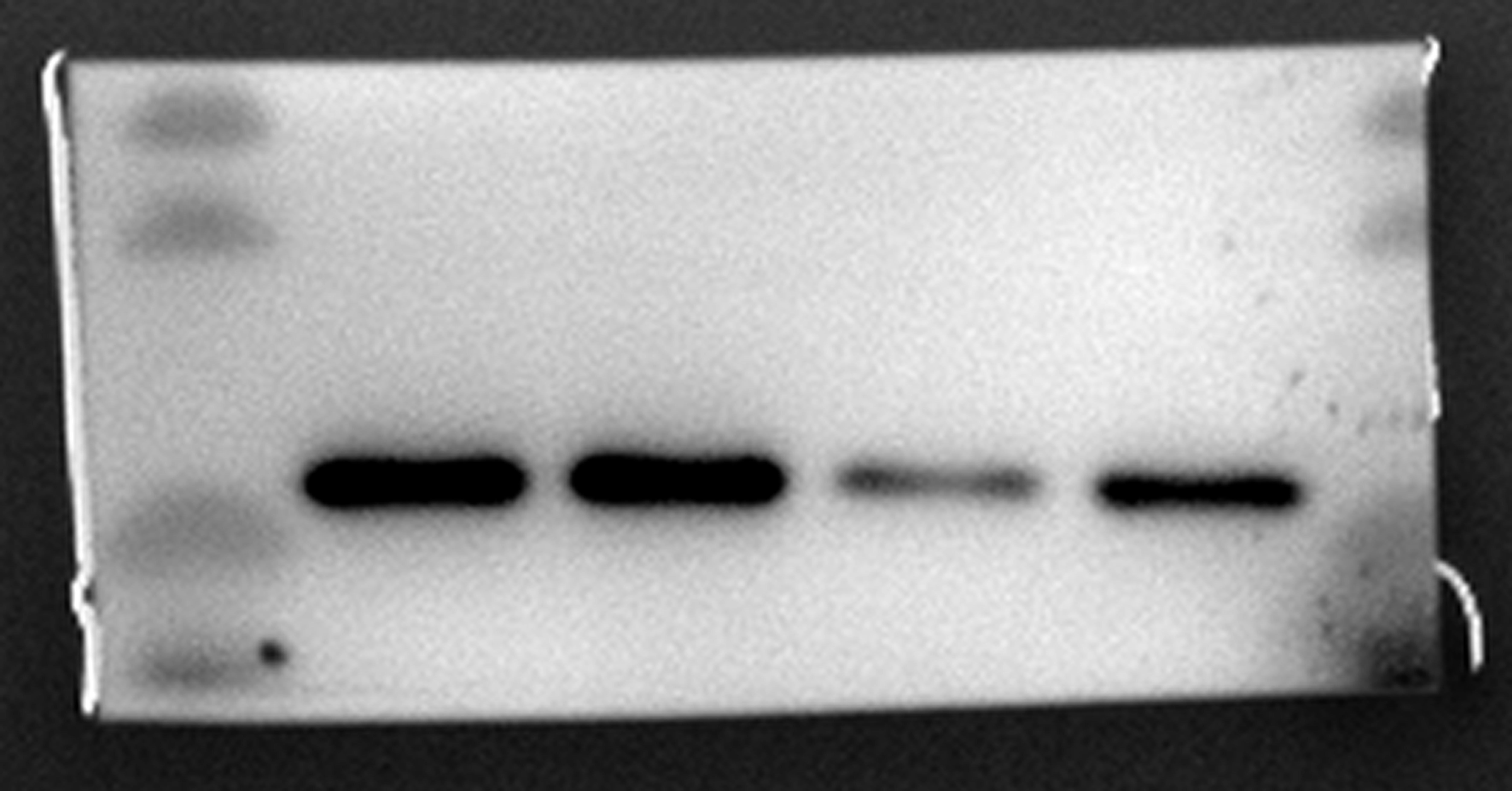

Supplement: Supplemental Material [file KBIE_A_2057632_SM9317.zip › supplementary/Fig2B_claudin_1.tif]

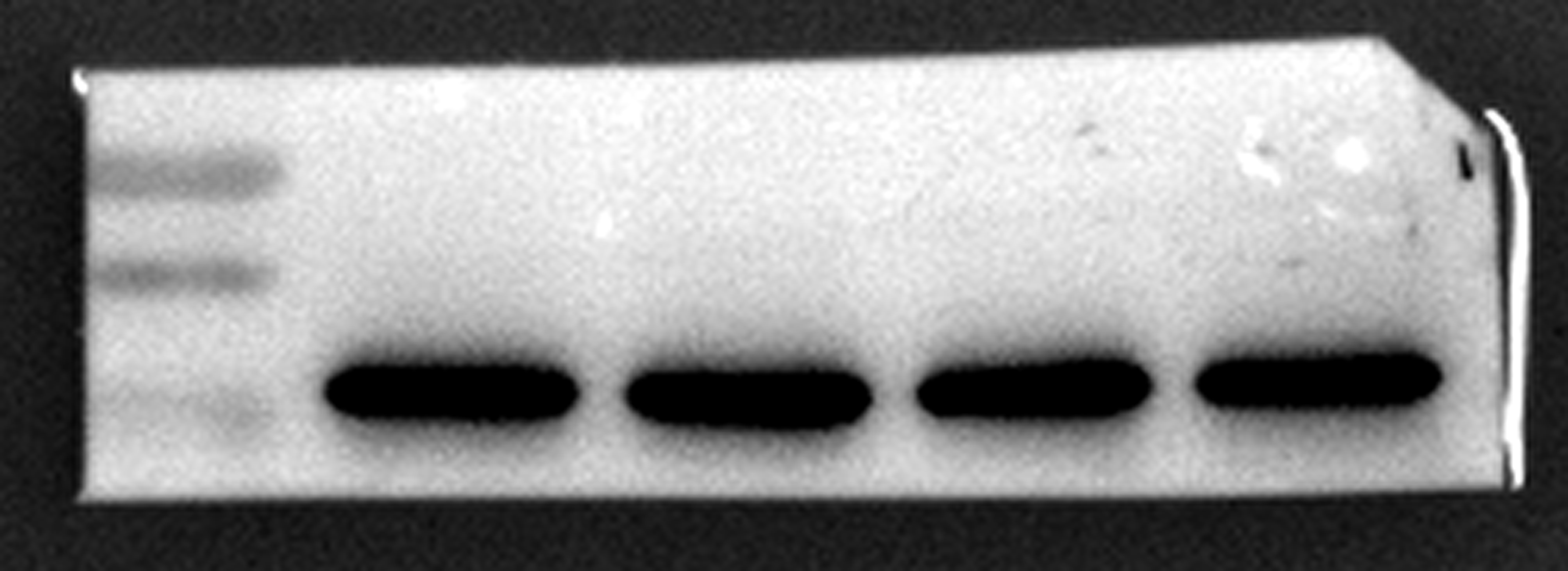

Supplement: Supplemental Material [file KBIE_A_2057632_SM9317.zip › supplementary/Fig2B_GAPDH.tif]

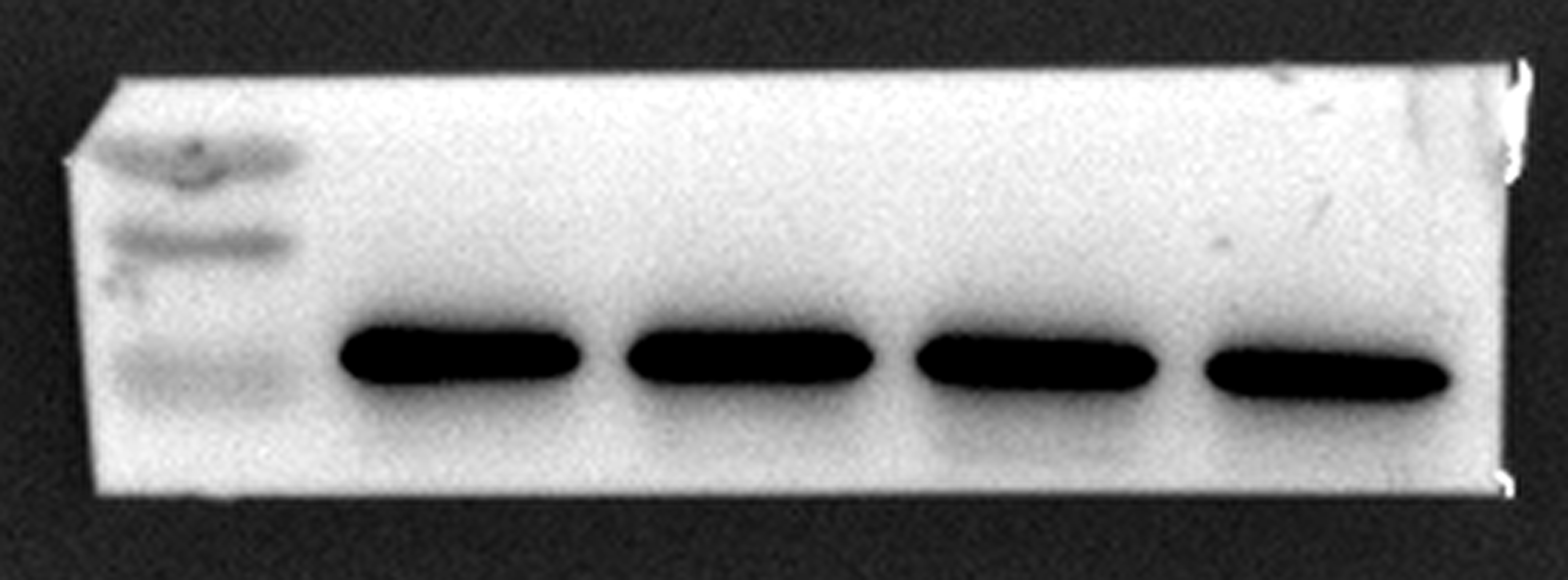

Supplement: Supplemental Material [file KBIE_A_2057632_SM9317.zip › supplementary/Fig2B_GAPDH_1.tif]

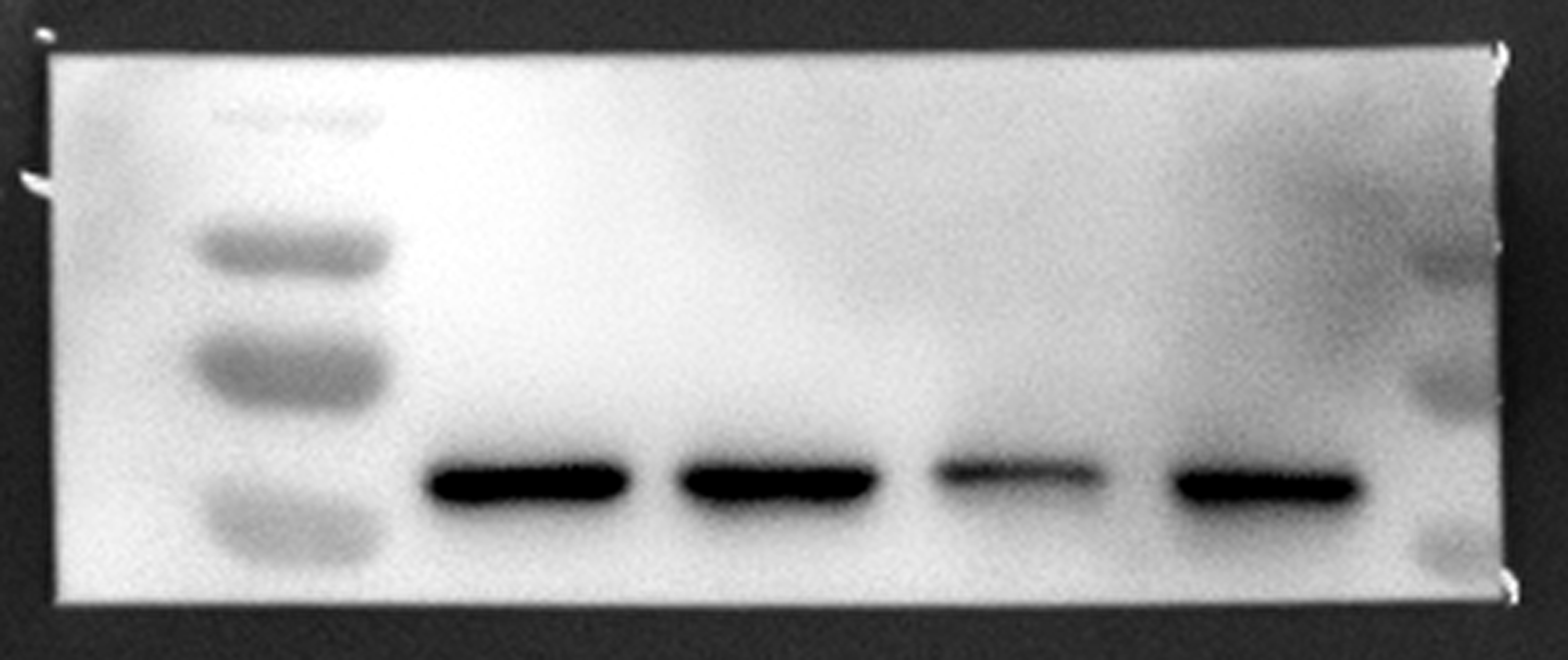

Supplement: Supplemental Material [file KBIE_A_2057632_SM9317.zip › supplementary/Fig2B_occludin.tif]

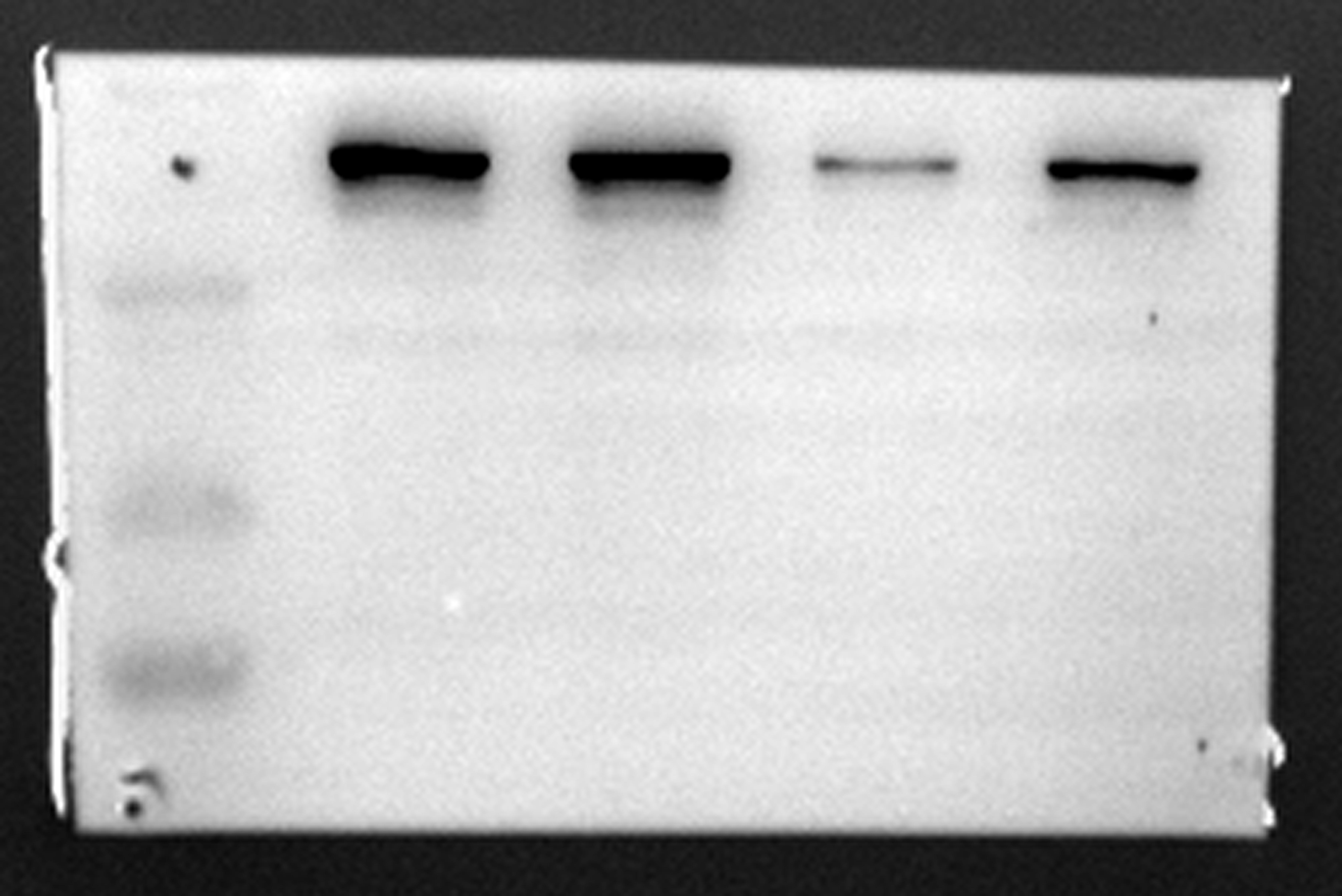

Supplement: Supplemental Material [file KBIE_A_2057632_SM9317.zip › supplementary/Fig2B_ZO_1.tif]

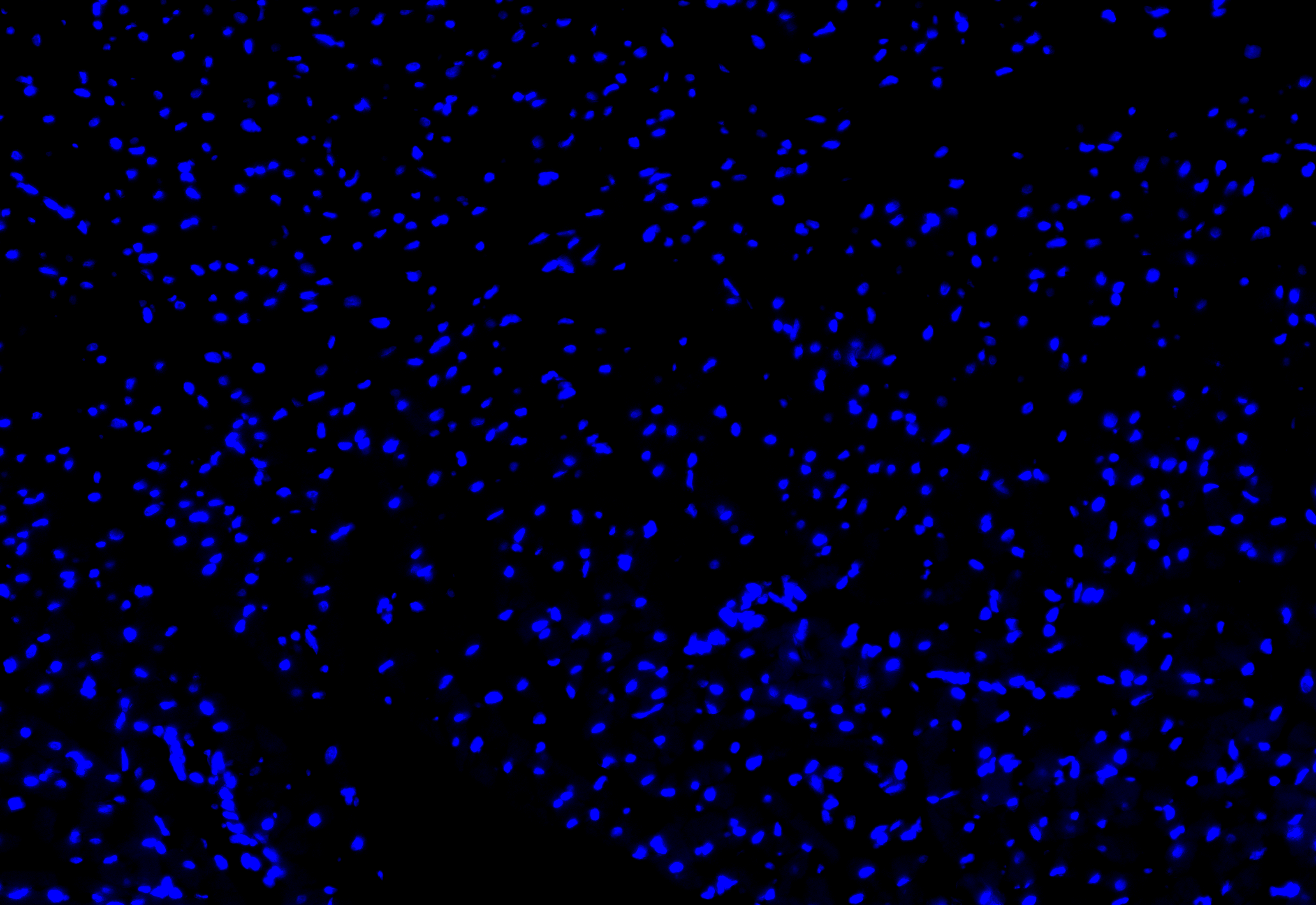

Supplement: Supplemental Material [file KBIE_A_2057632_SM9317.zip › supplementary/Fig2D_Control_DAPI.tif]

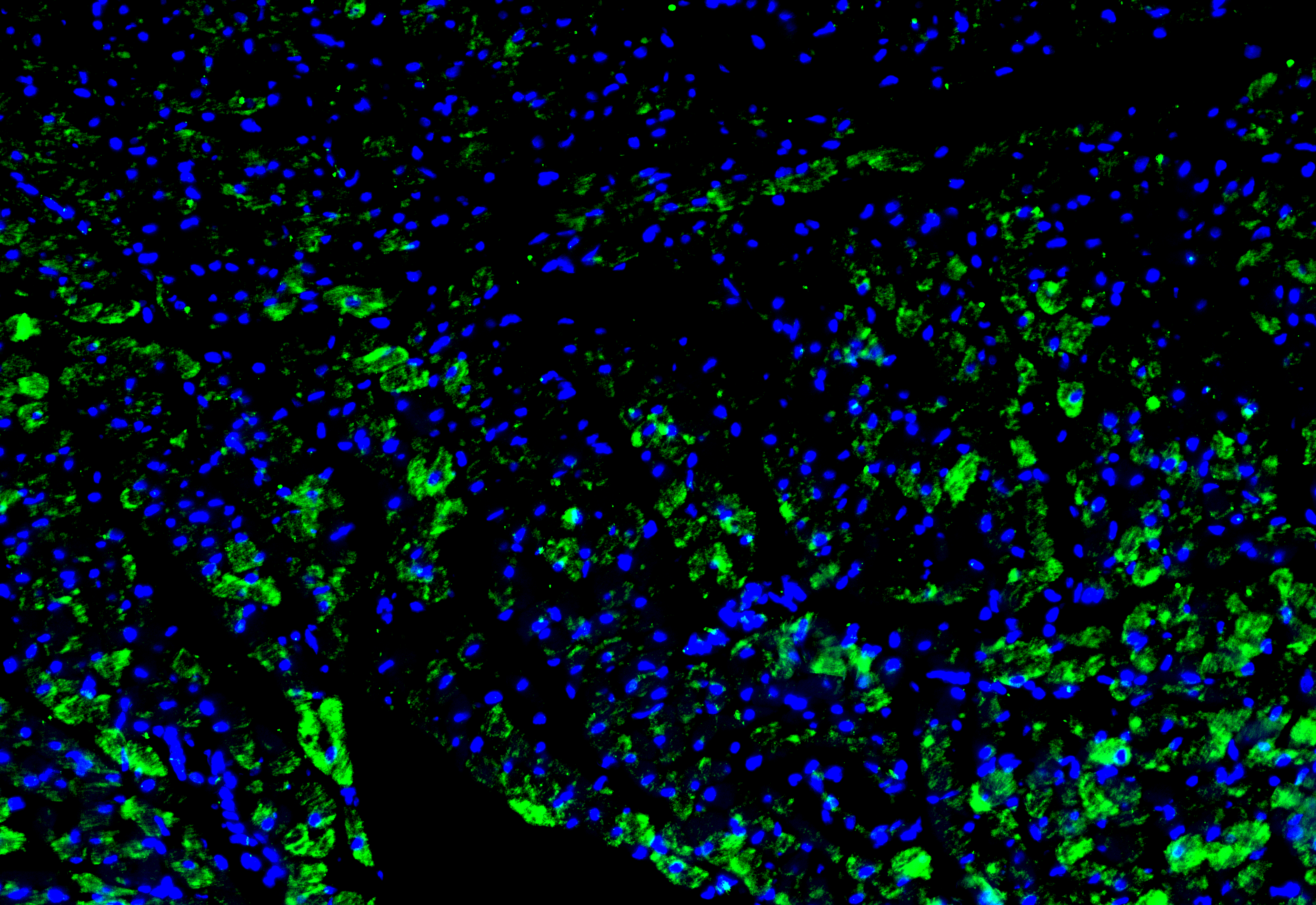

Supplement: Supplemental Material [file KBIE_A_2057632_SM9317.zip › supplementary/Fig2D_Control_Merged.tif]

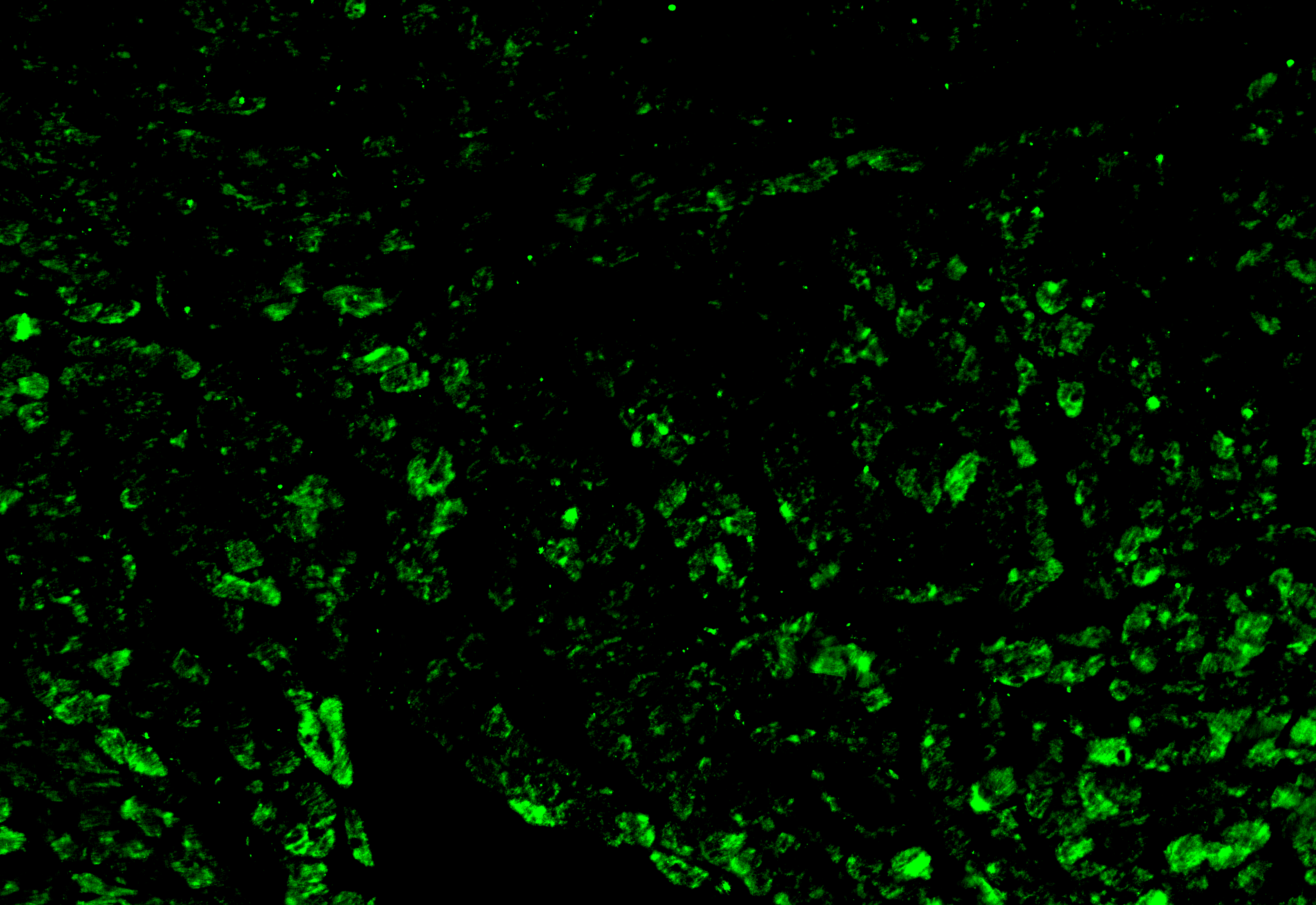

Supplement: Supplemental Material [file KBIE_A_2057632_SM9317.zip › supplementary/Fig2D_Control_ZO_1.tif]

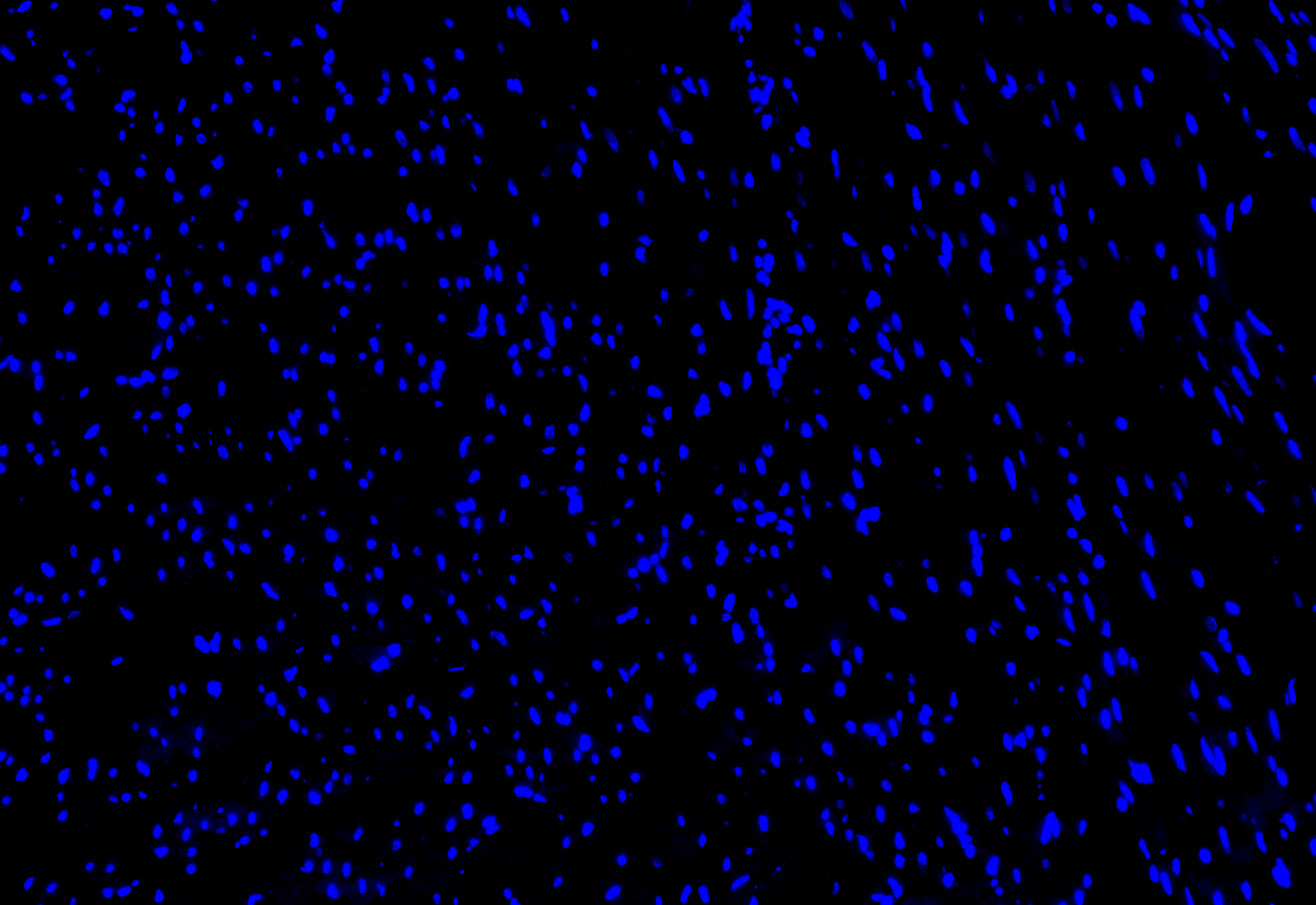

Supplement: Supplemental Material [file KBIE_A_2057632_SM9317.zip › supplementary/Fig2D_IR_DAPI.tif]

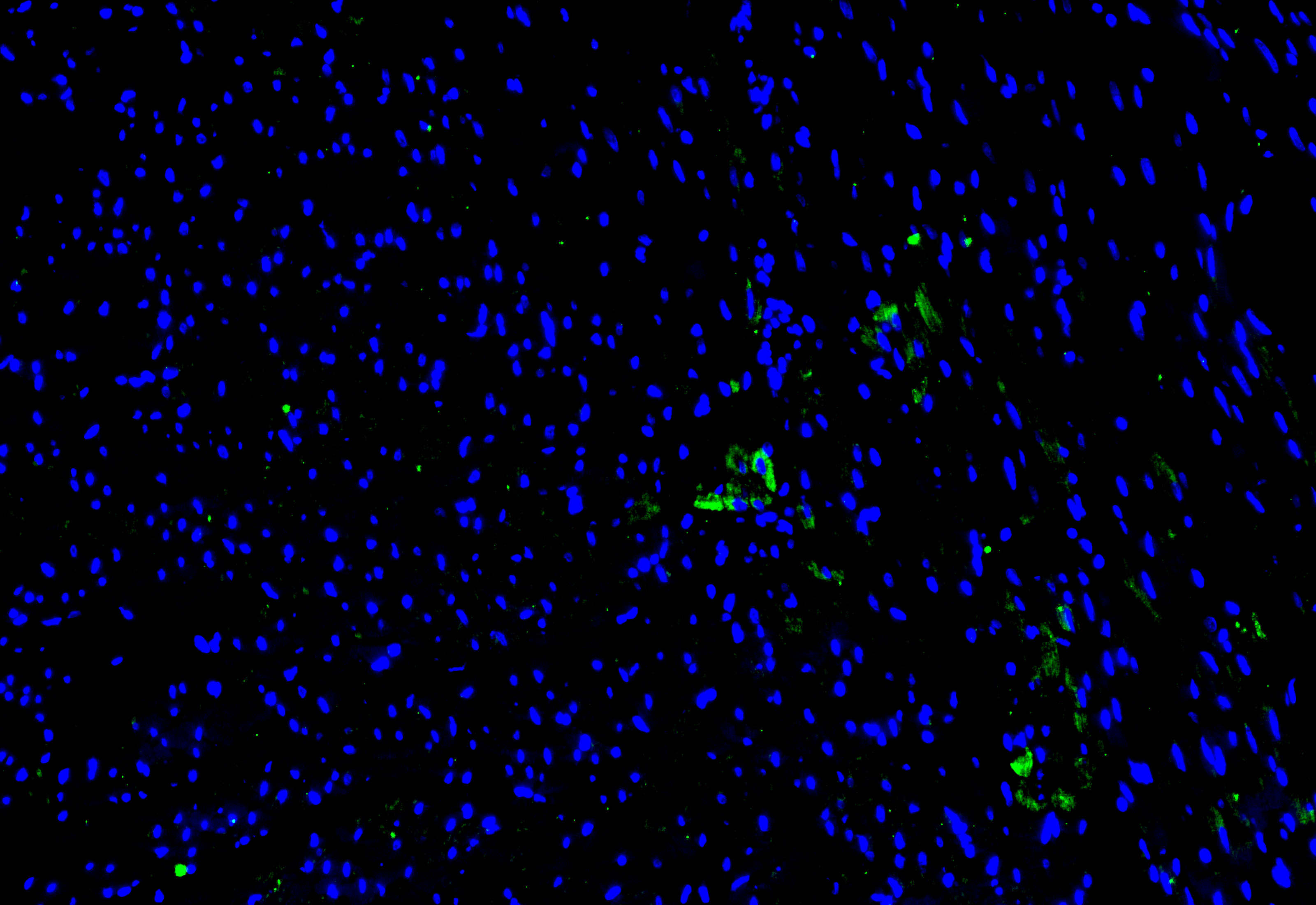

Supplement: Supplemental Material [file KBIE_A_2057632_SM9317.zip › supplementary/Fig2D_IR_Merged.tif]

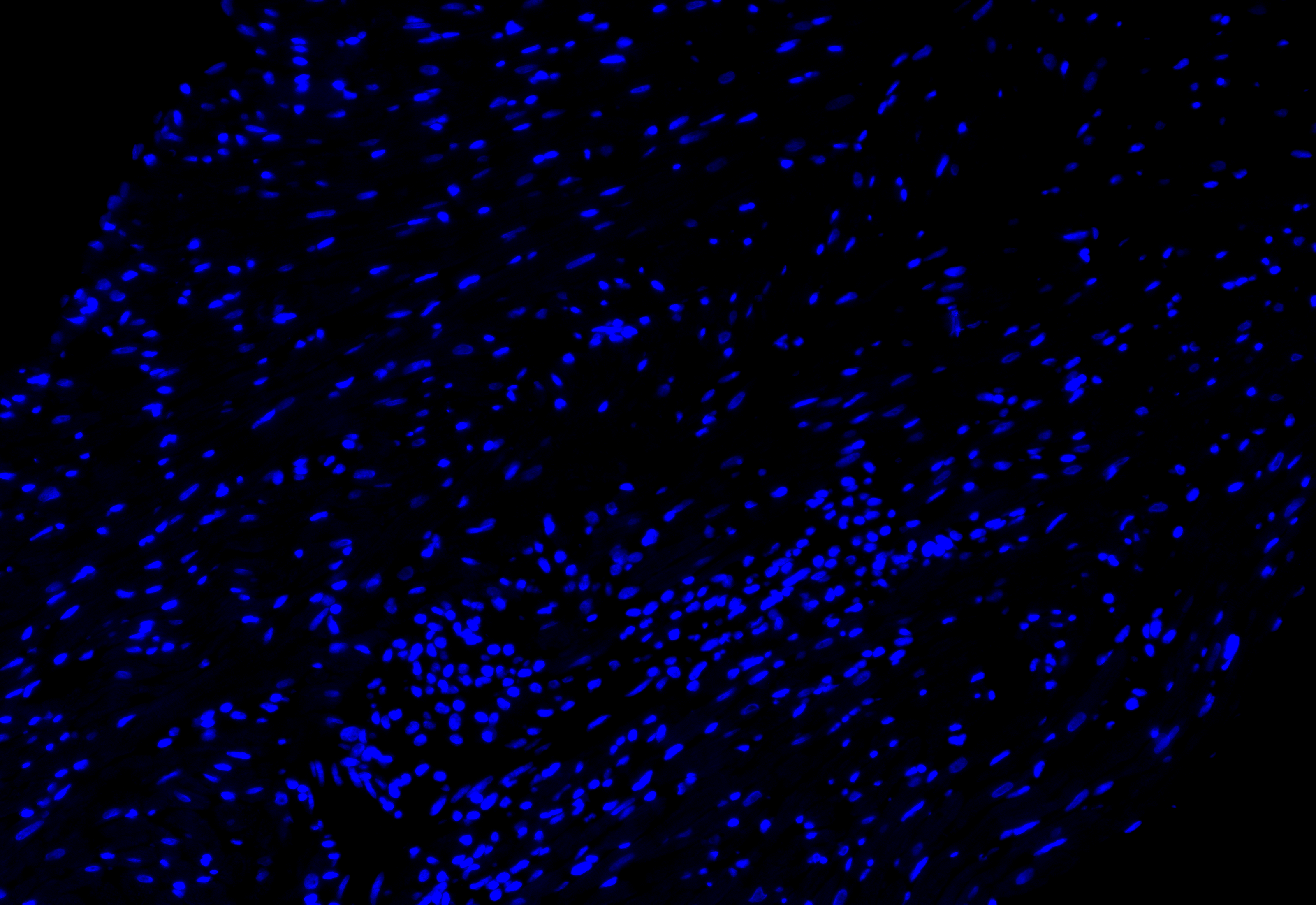

Supplement: Supplemental Material [file KBIE_A_2057632_SM9317.zip › supplementary/Fig2D_IR_Oxycodone_DAPI.tif]

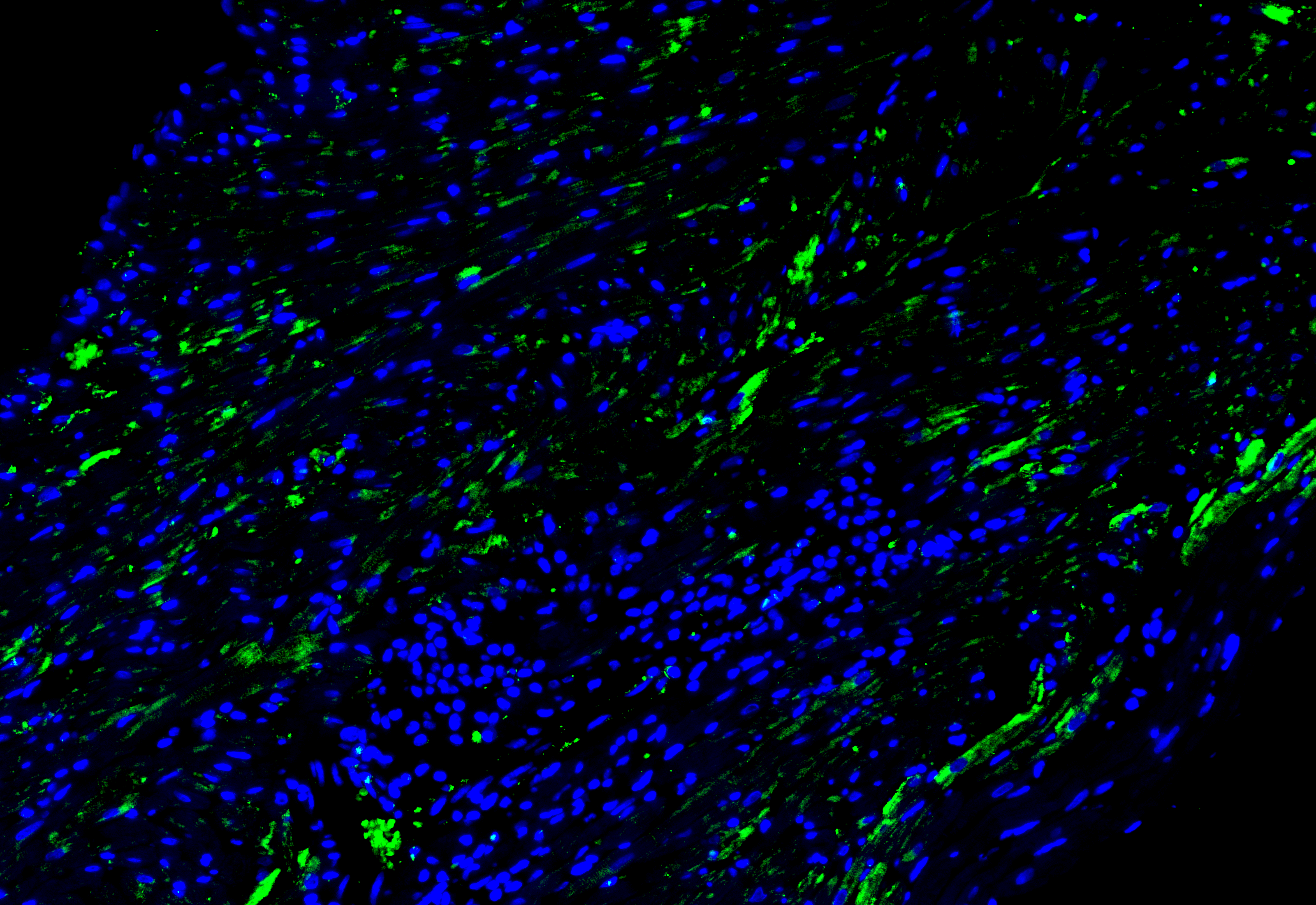

Supplement: Supplemental Material [file KBIE_A_2057632_SM9317.zip › supplementary/Fig2D_IR_Oxycodone_Merged.tif]

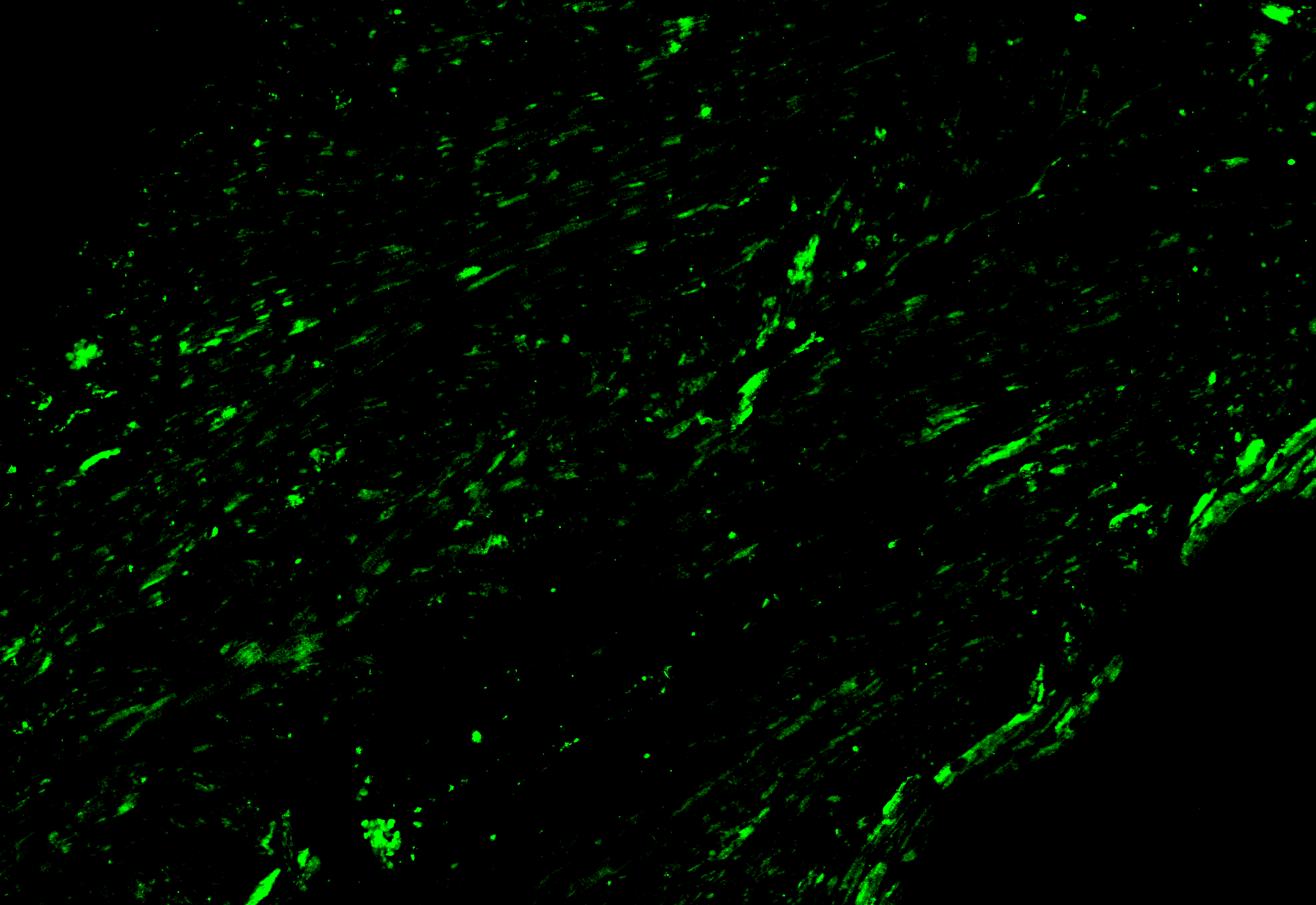

Supplement: Supplemental Material [file KBIE_A_2057632_SM9317.zip › supplementary/Fig2D_IR_Oxycodone_ZO_1.tif]

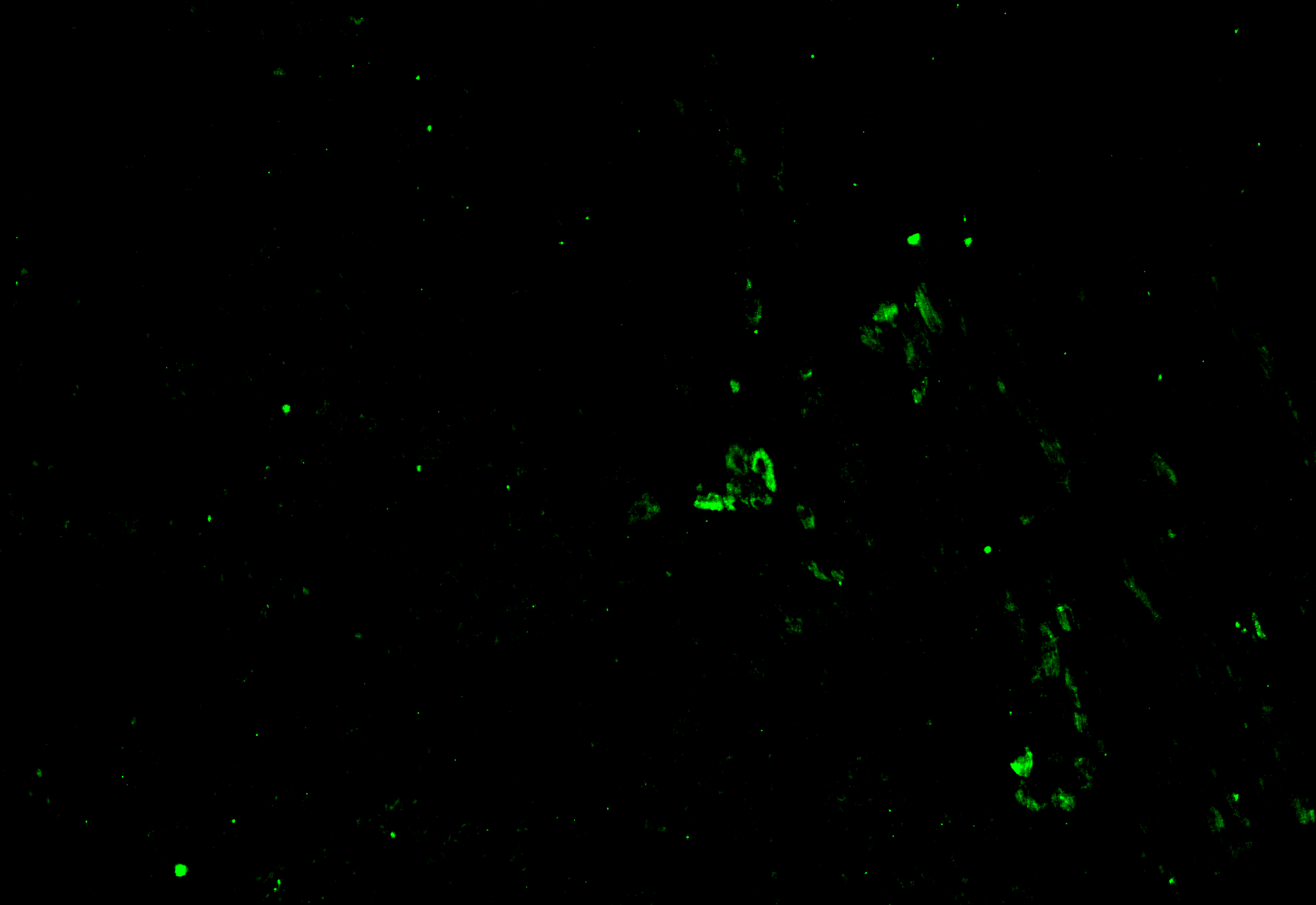

Supplement: Supplemental Material [file KBIE_A_2057632_SM9317.zip › supplementary/Fig2D_IR_ZO_1.tif]

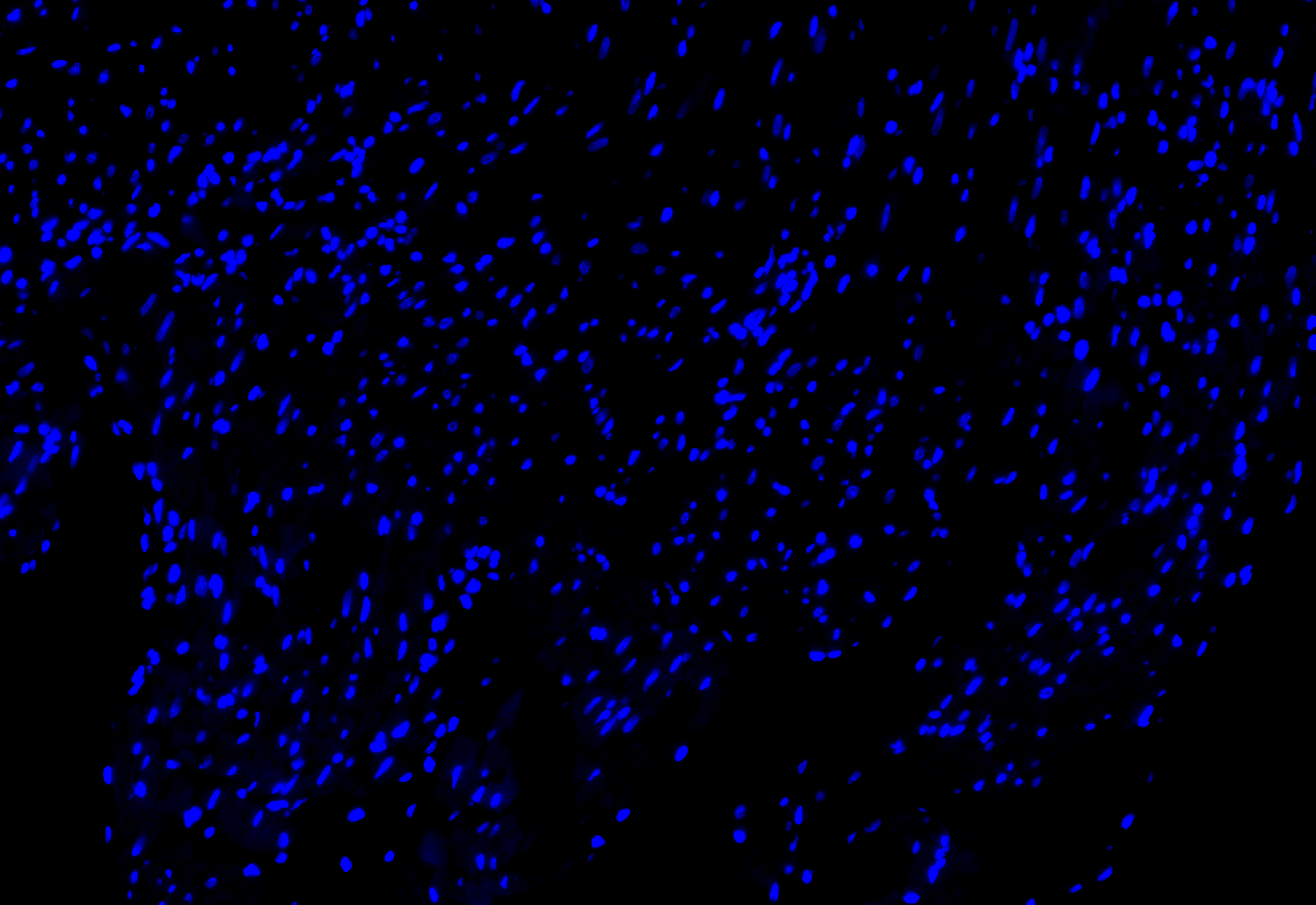

Supplement: Supplemental Material [file KBIE_A_2057632_SM9317.zip › supplementary/Fig2D_Sham_DAPI.tif]

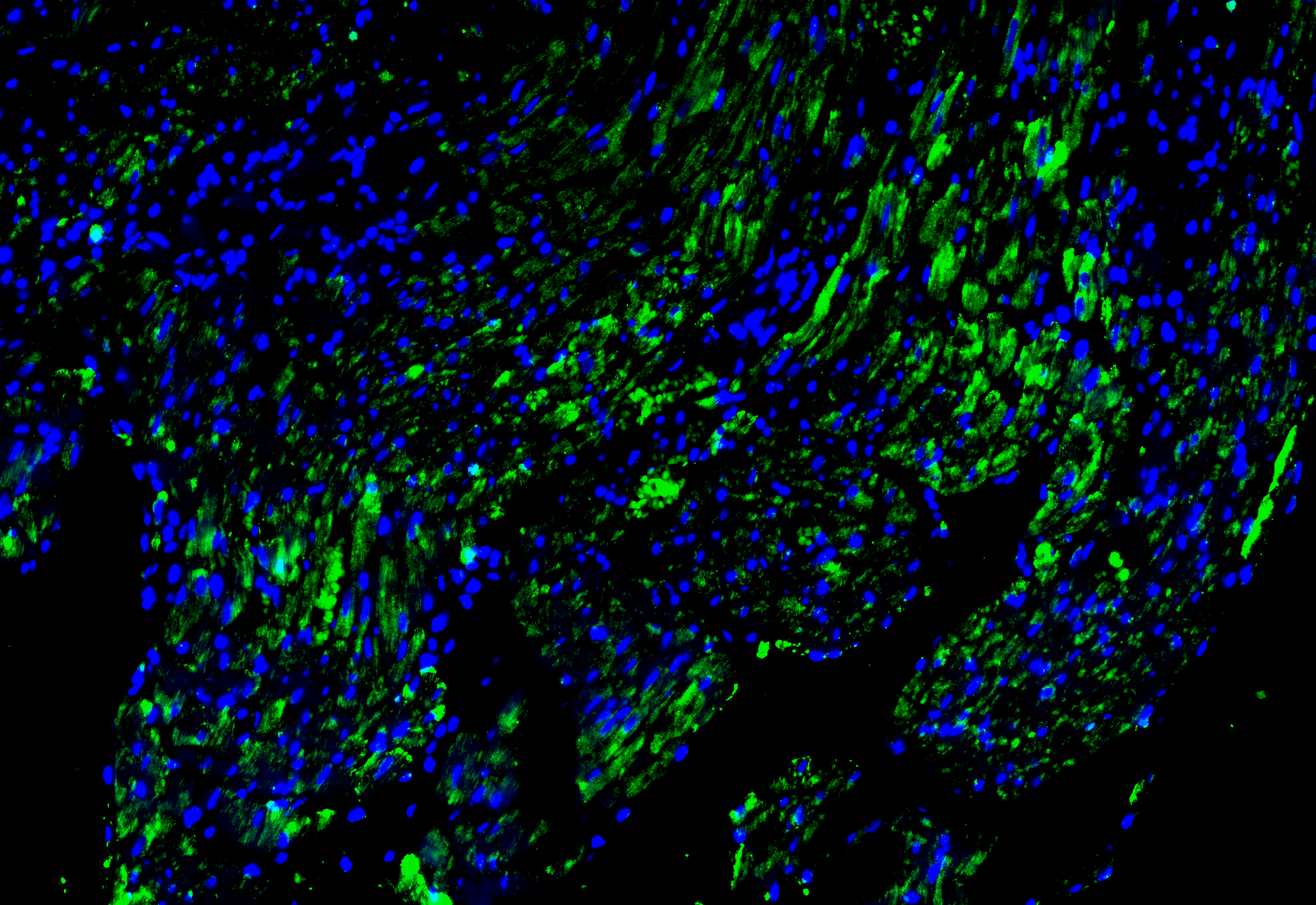

Supplement: Supplemental Material [file KBIE_A_2057632_SM9317.zip › supplementary/Fig2D_Sham_Merged.tif]

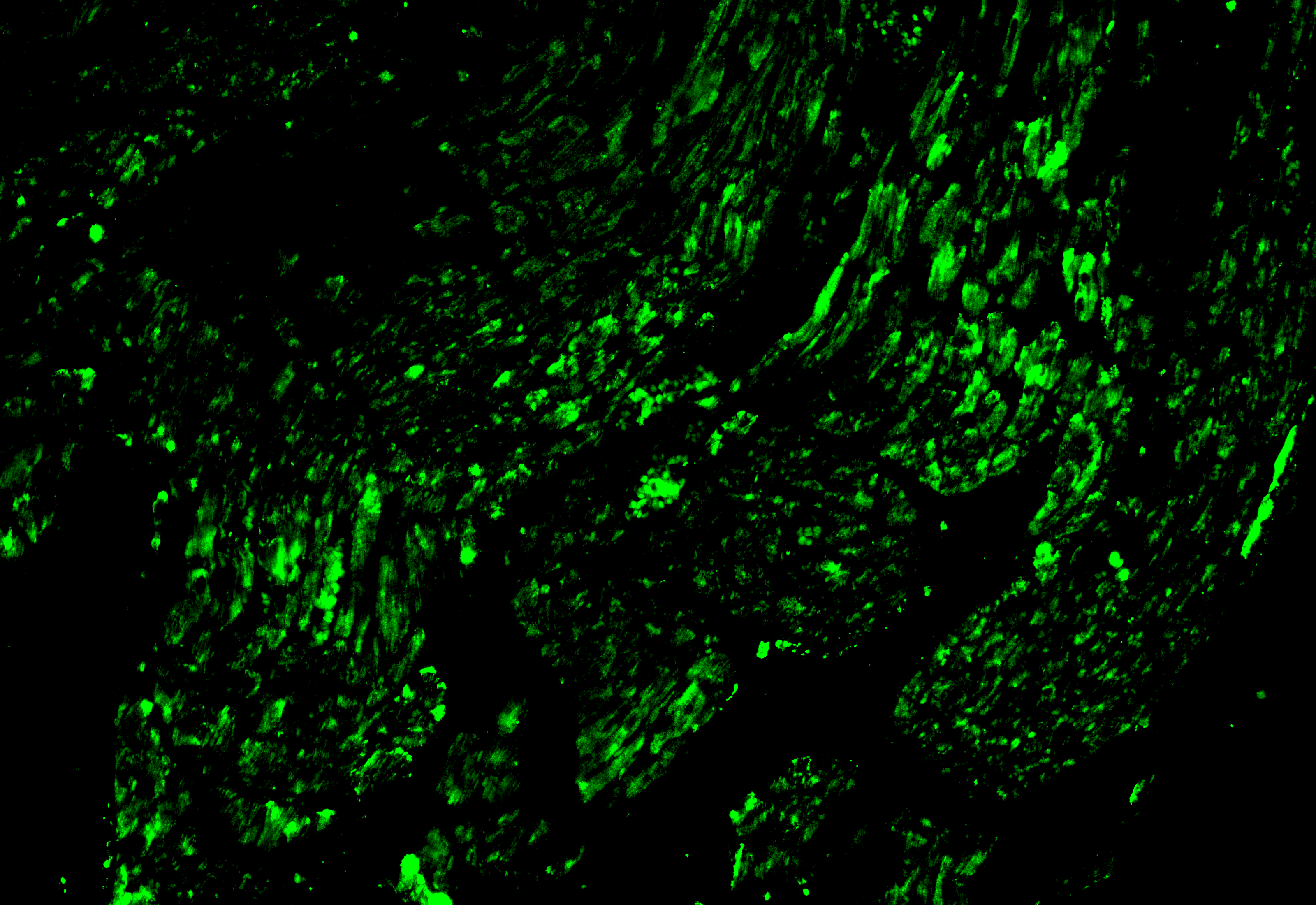

Supplement: Supplemental Material [file KBIE_A_2057632_SM9317.zip › supplementary/Fig2D_Sham_ZO_1.tif]

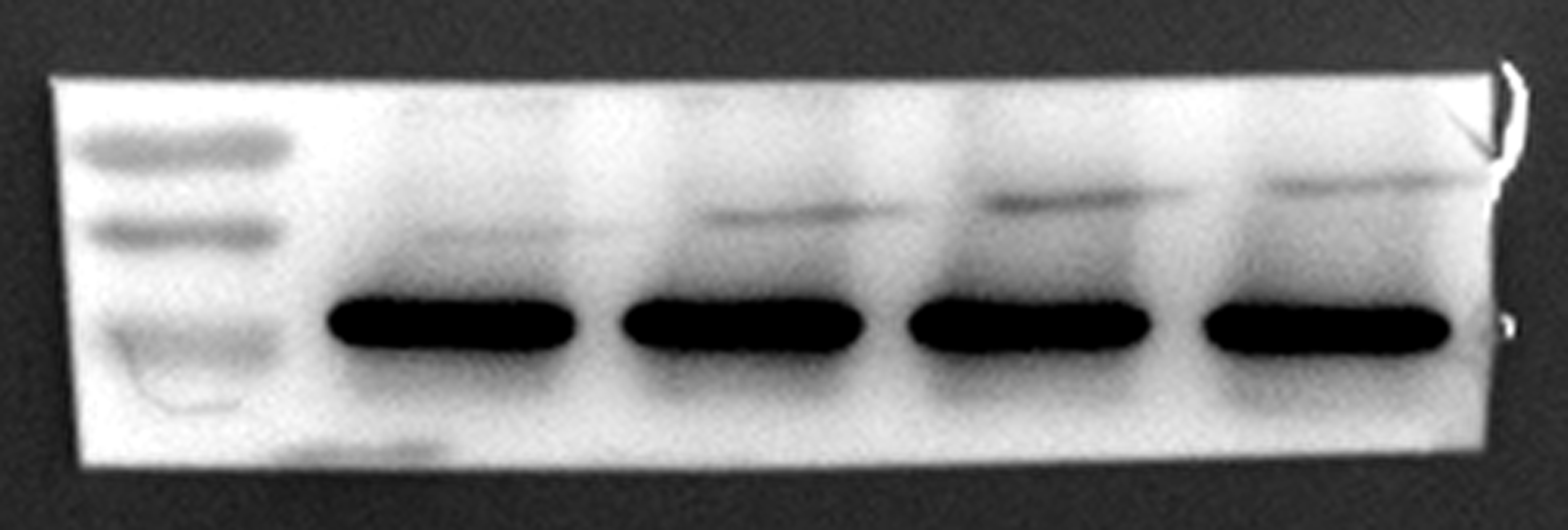

Supplement: Supplemental Material [file KBIE_A_2057632_SM9317.zip › supplementary/Fig3C_GAPDH.tif]

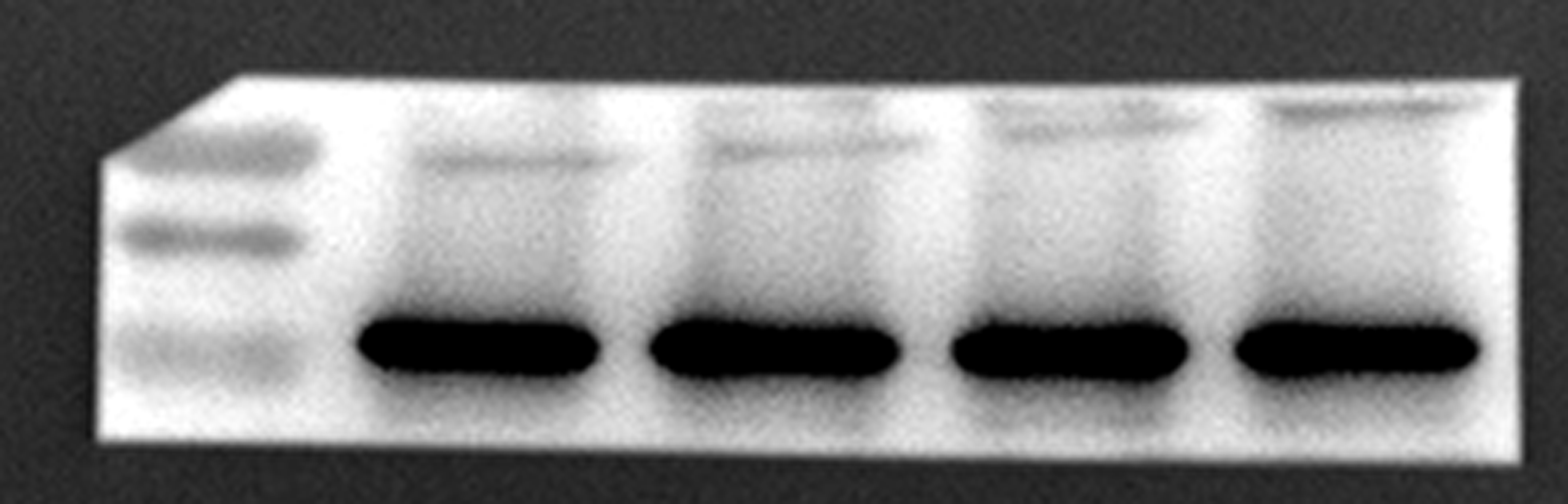

Supplement: Supplemental Material [file KBIE_A_2057632_SM9317.zip › supplementary/Fig3C_GAPDH_1.tif]

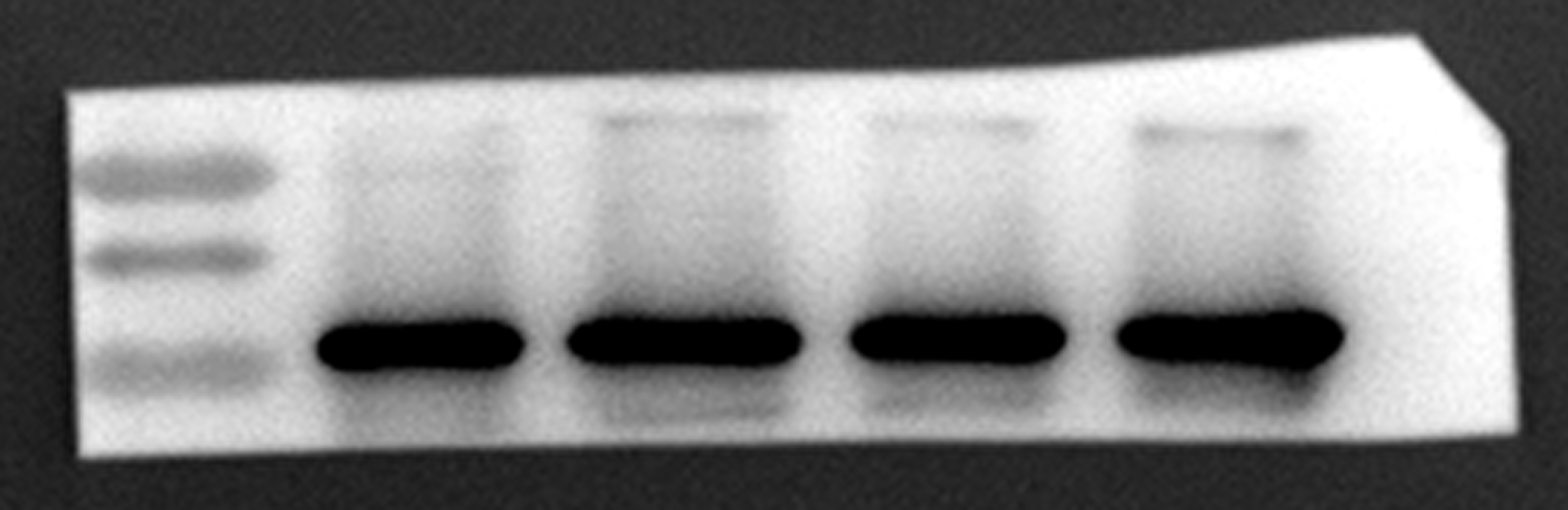

Supplement: Supplemental Material [file KBIE_A_2057632_SM9317.zip › supplementary/Fig3C_GAPDH_2.tif]

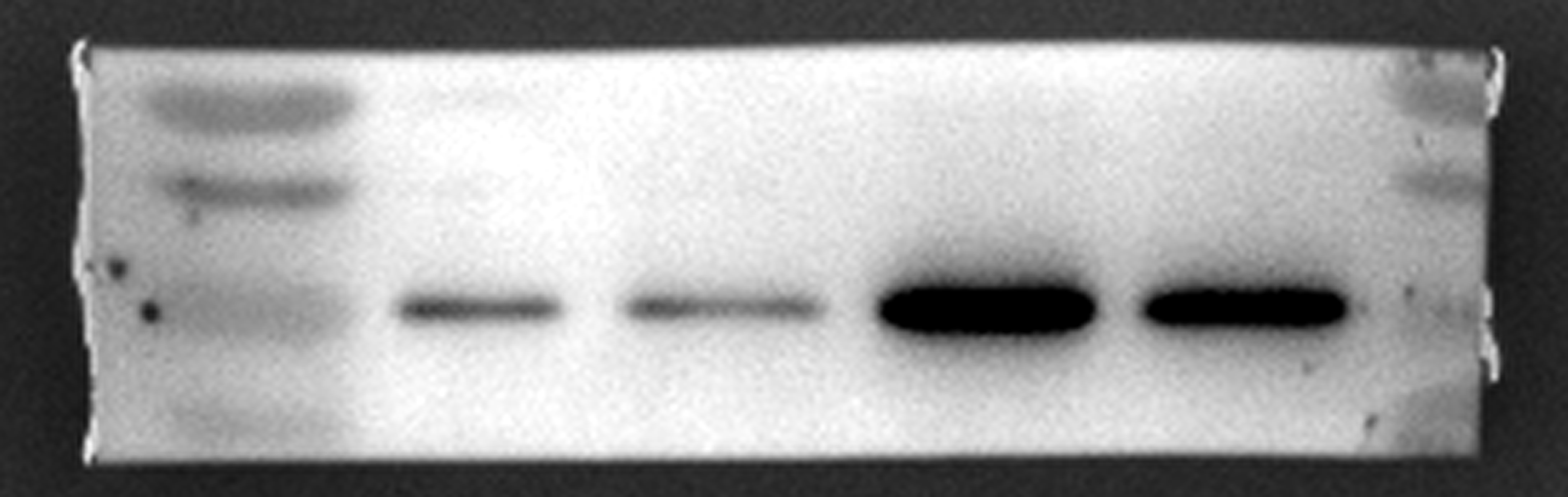

Supplement: Supplemental Material [file KBIE_A_2057632_SM9317.zip › supplementary/Fig3C_IL_1b.tif]

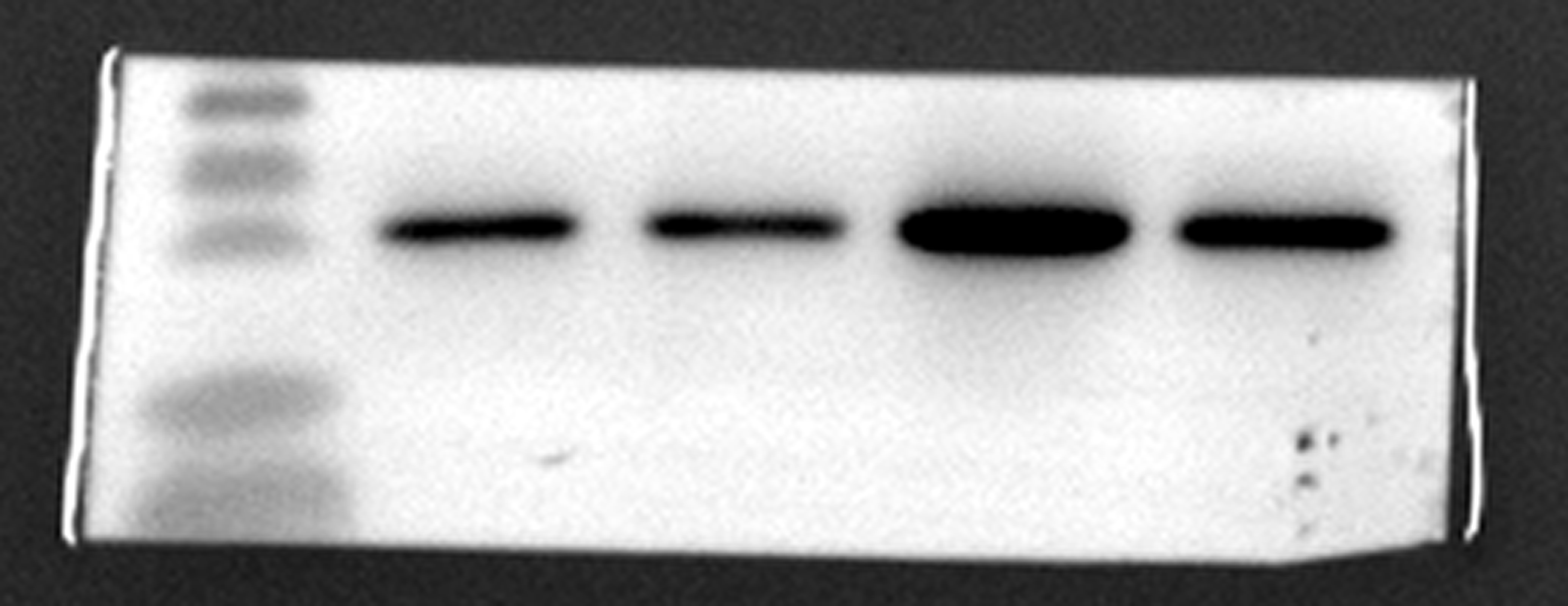

Supplement: Supplemental Material [file KBIE_A_2057632_SM9317.zip › supplementary/Fig3C_IL_6.tif]

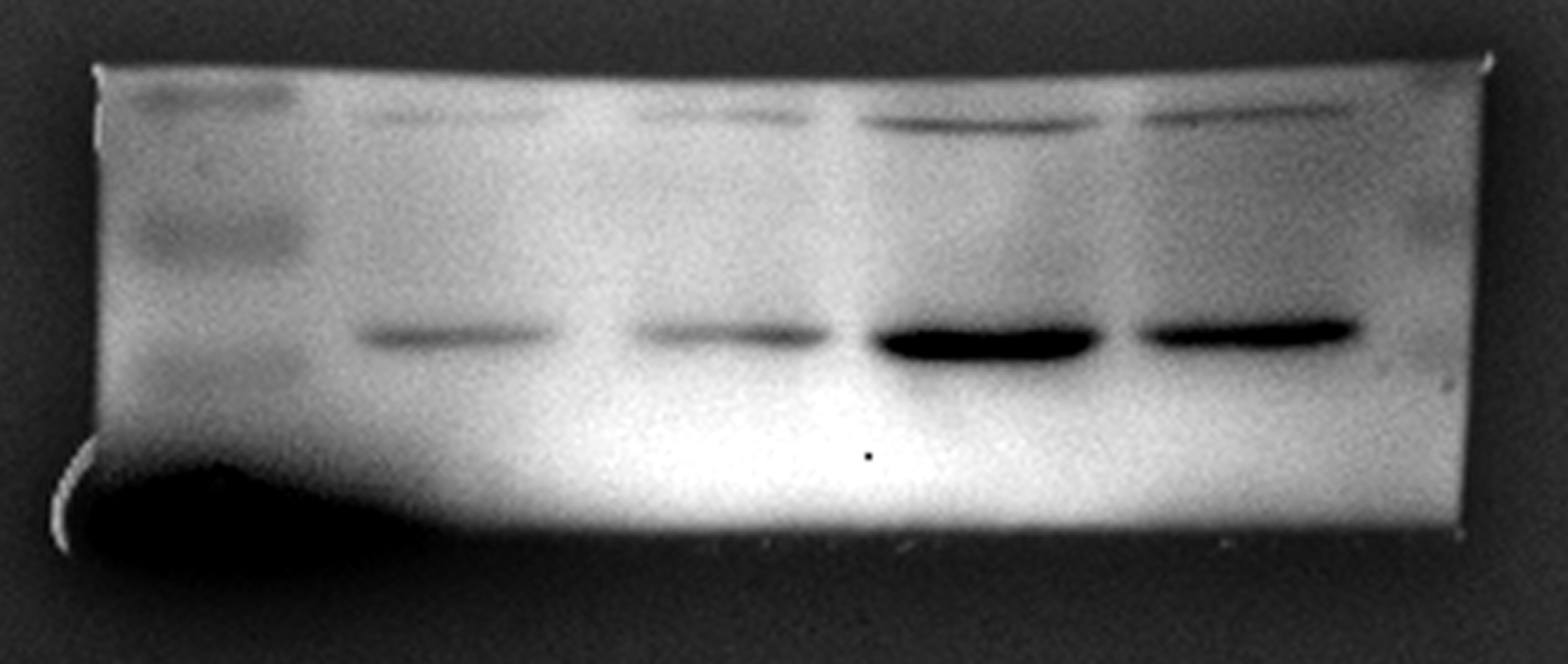

Supplement: Supplemental Material [file KBIE_A_2057632_SM9317.zip › supplementary/Fig3C_TNF_a.tif]

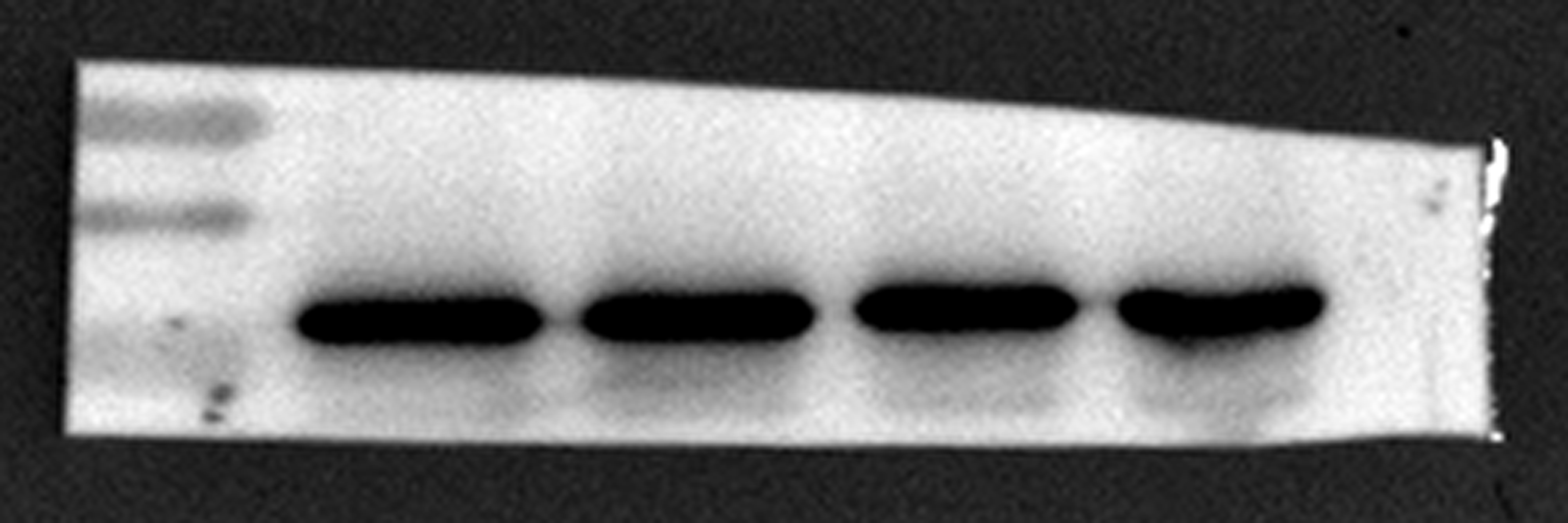

Supplement: Supplemental Material [file KBIE_A_2057632_SM9317.zip › supplementary/Fig3D_GAPDH.tif]

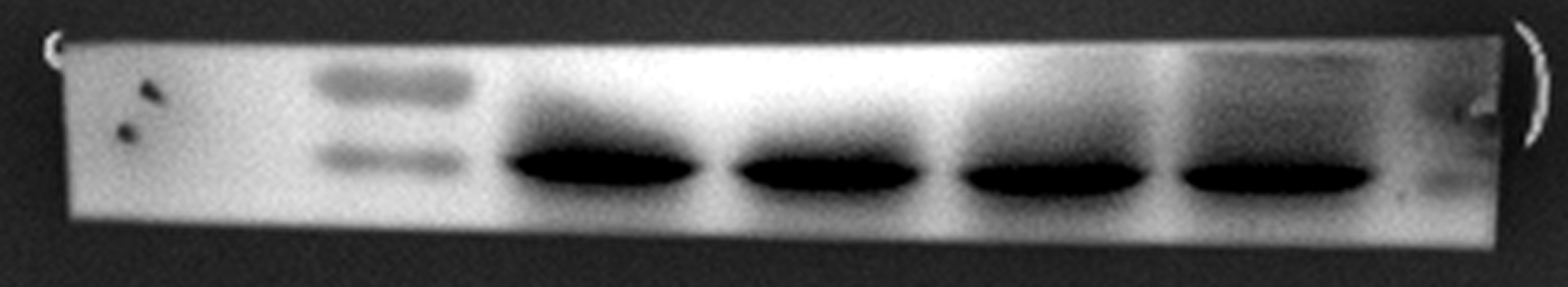

Supplement: Supplemental Material [file KBIE_A_2057632_SM9317.zip › supplementary/Fig3D_Ikba.tif]

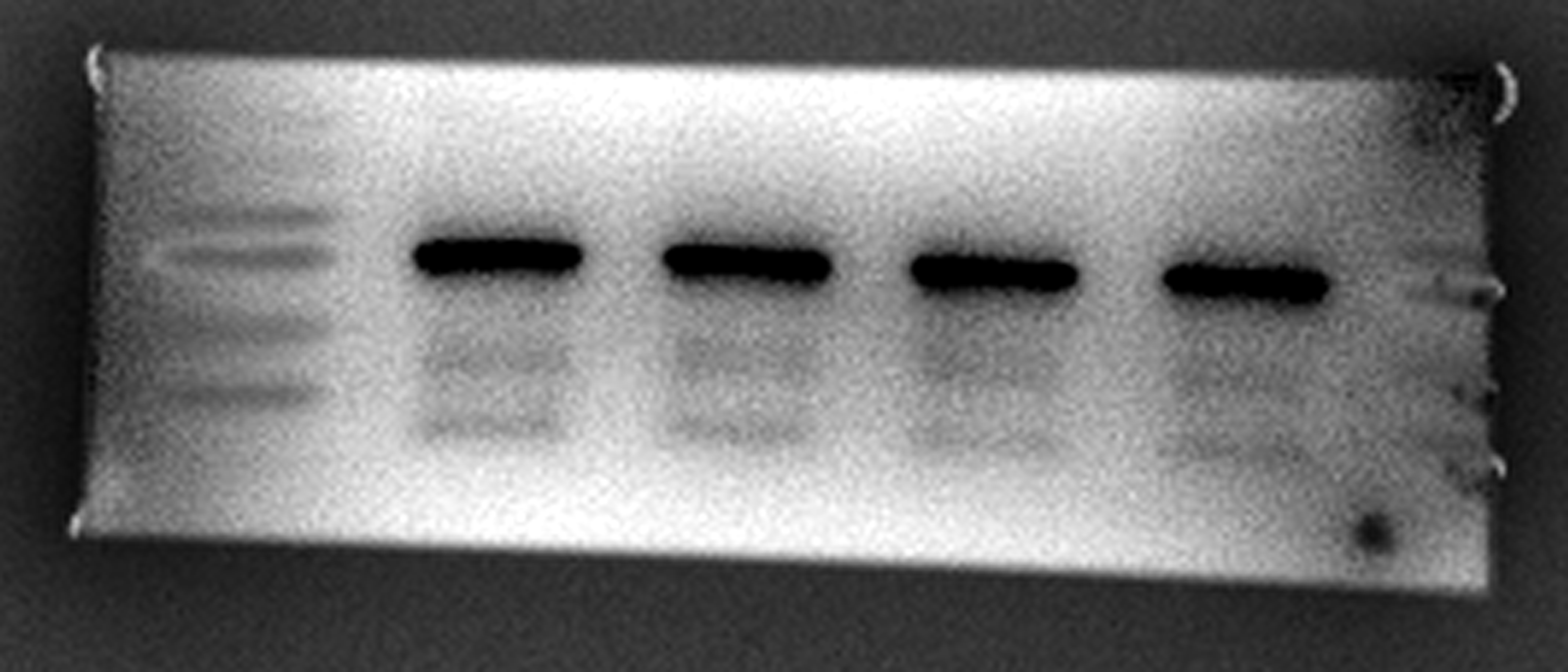

Supplement: Supplemental Material [file KBIE_A_2057632_SM9317.zip › supplementary/Fig3D_NF_kB_p65.tif]

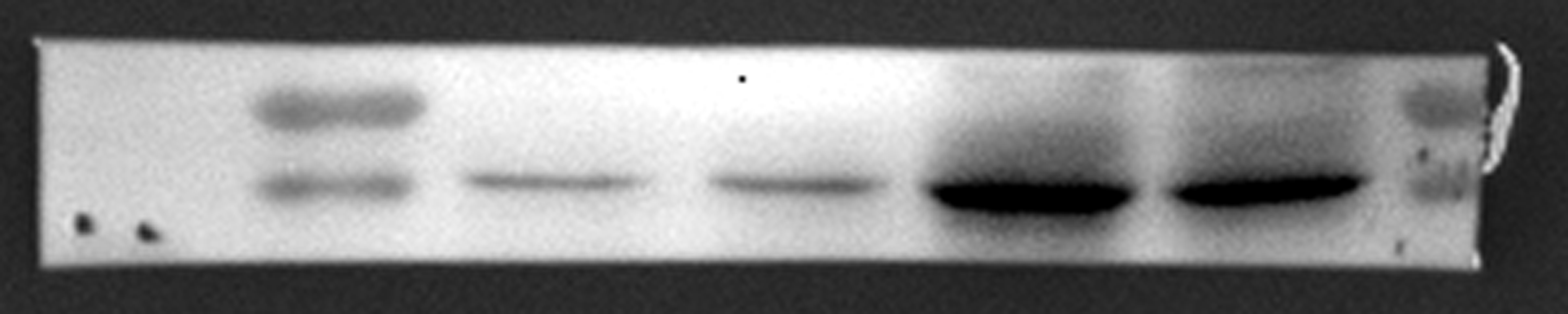

Supplement: Supplemental Material [file KBIE_A_2057632_SM9317.zip › supplementary/Fig3D_p_Ikba.tif]

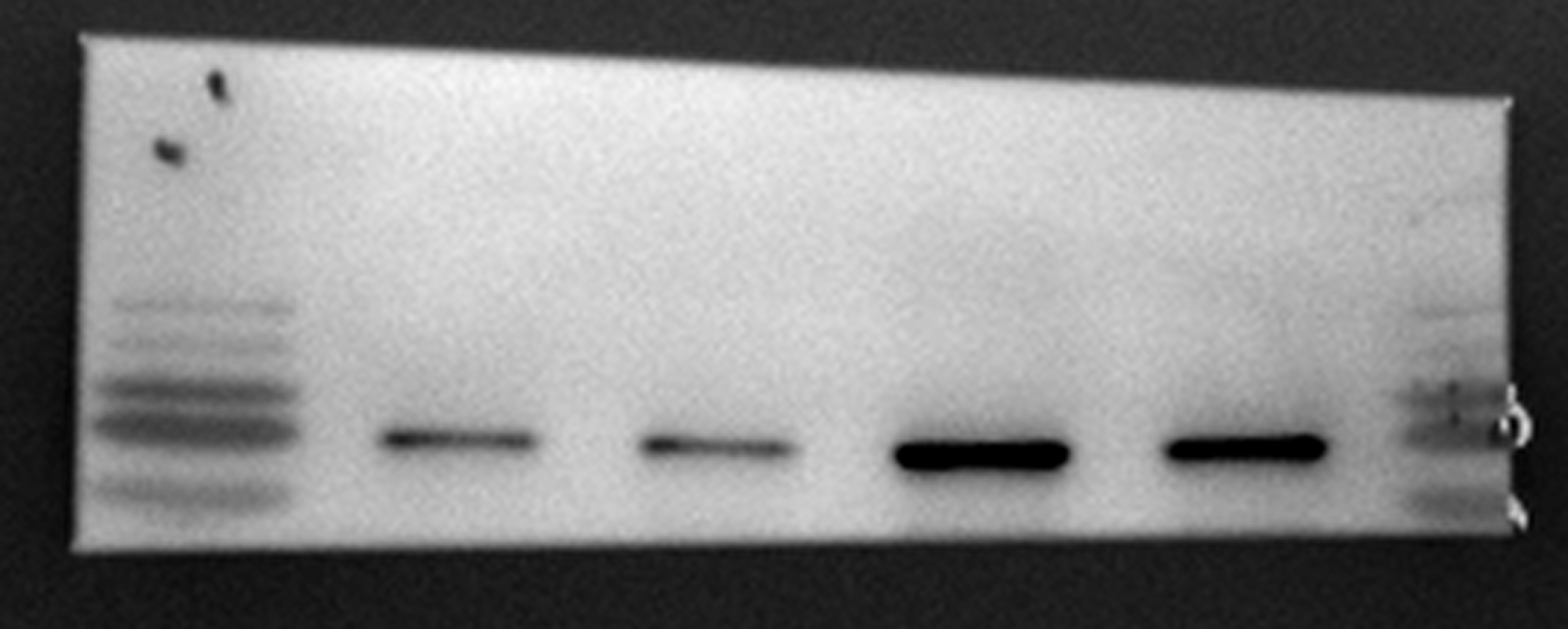

Supplement: Supplemental Material [file KBIE_A_2057632_SM9317.zip › supplementary/Fig3D_p_NF_kB_p65.tif]

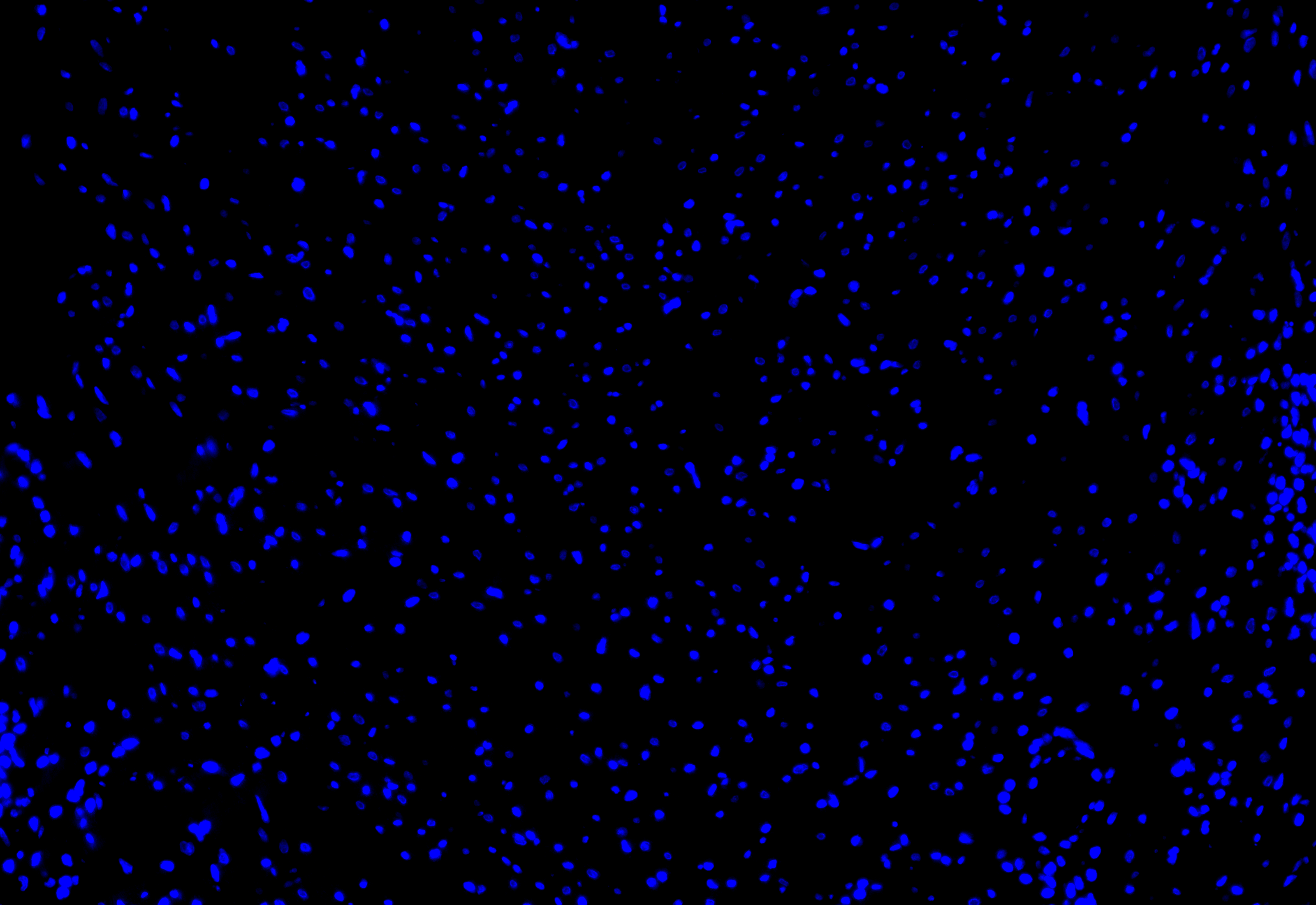

Supplement: Supplemental Material [file KBIE_A_2057632_SM9317.zip › supplementary/Fig4A_Control_DAPI.tif]

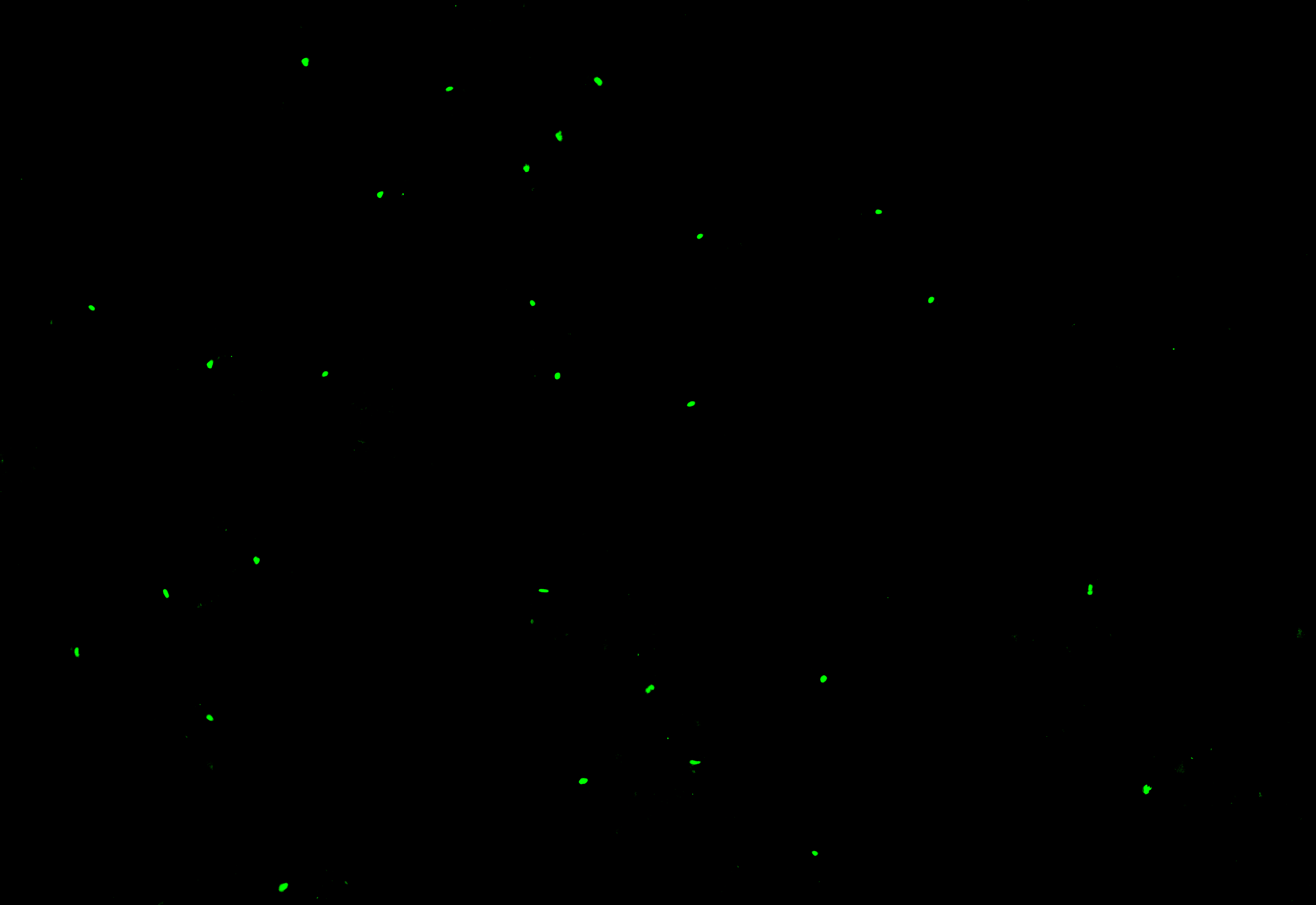

Supplement: Supplemental Material [file KBIE_A_2057632_SM9317.zip › supplementary/Fig4A_Control_Gr_1.tif]

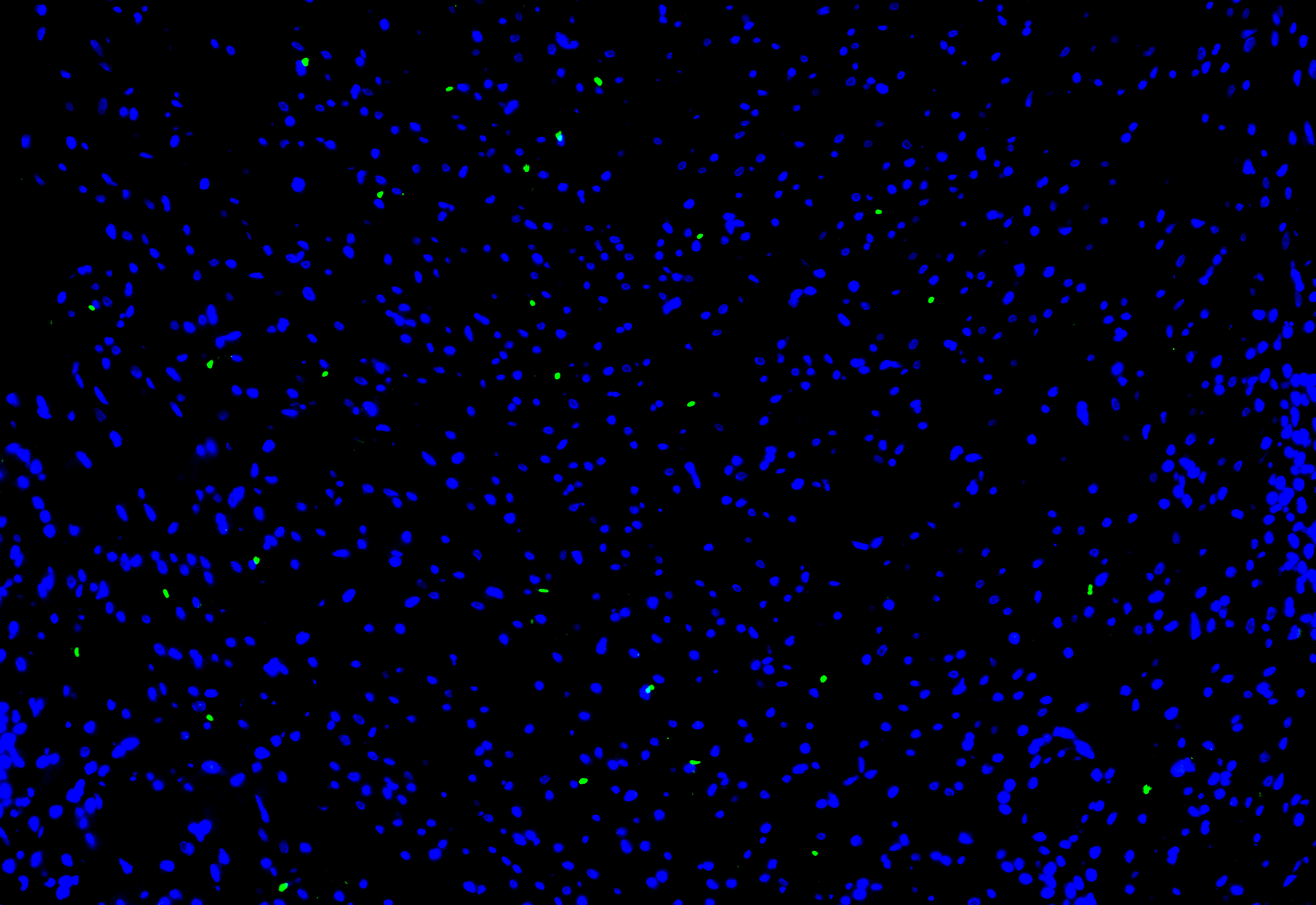

Supplement: Supplemental Material [file KBIE_A_2057632_SM9317.zip › supplementary/Fig4A_Control_Merged.tif]

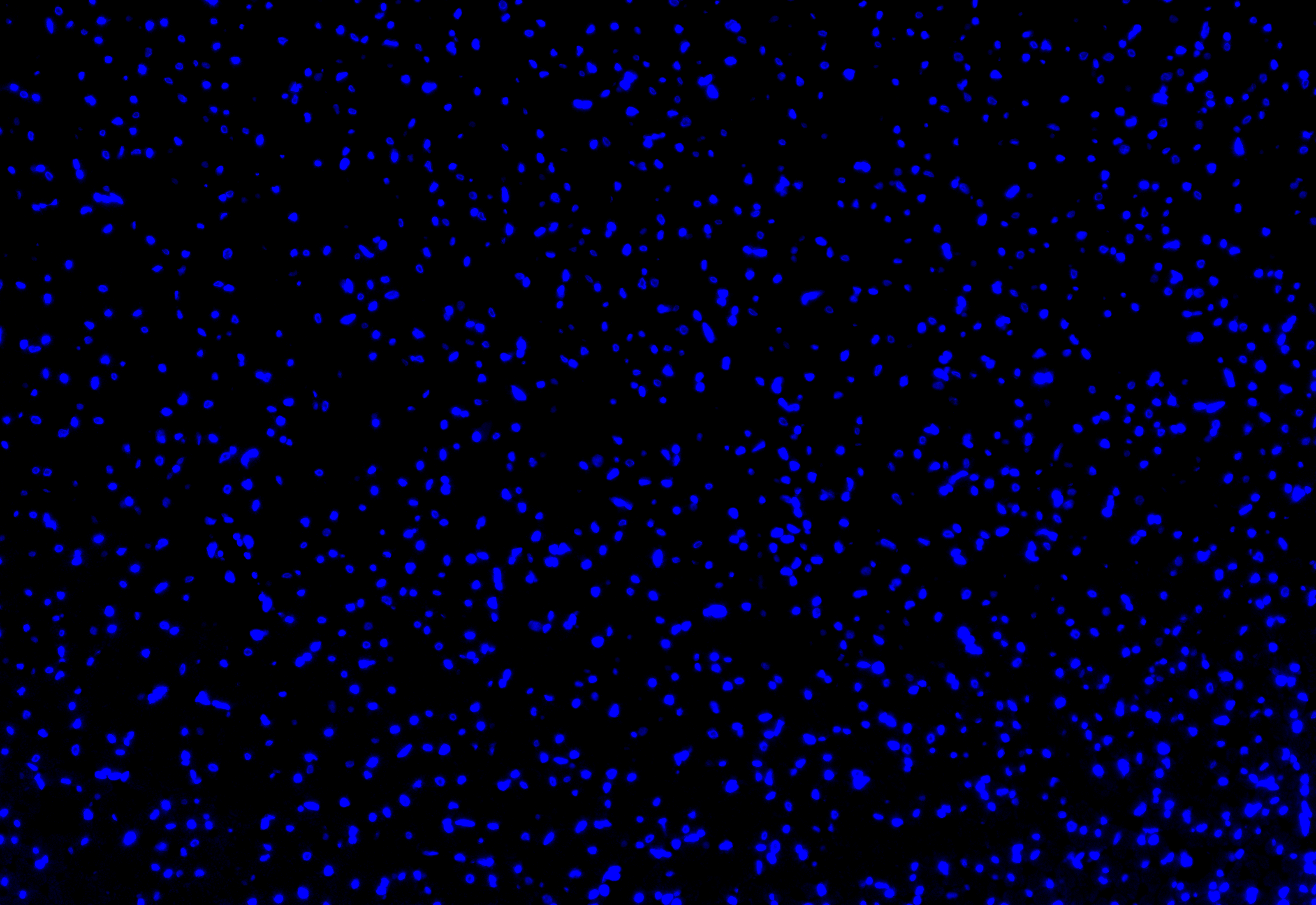

Supplement: Supplemental Material [file KBIE_A_2057632_SM9317.zip › supplementary/Fig4A_IR_DAPI.tif]

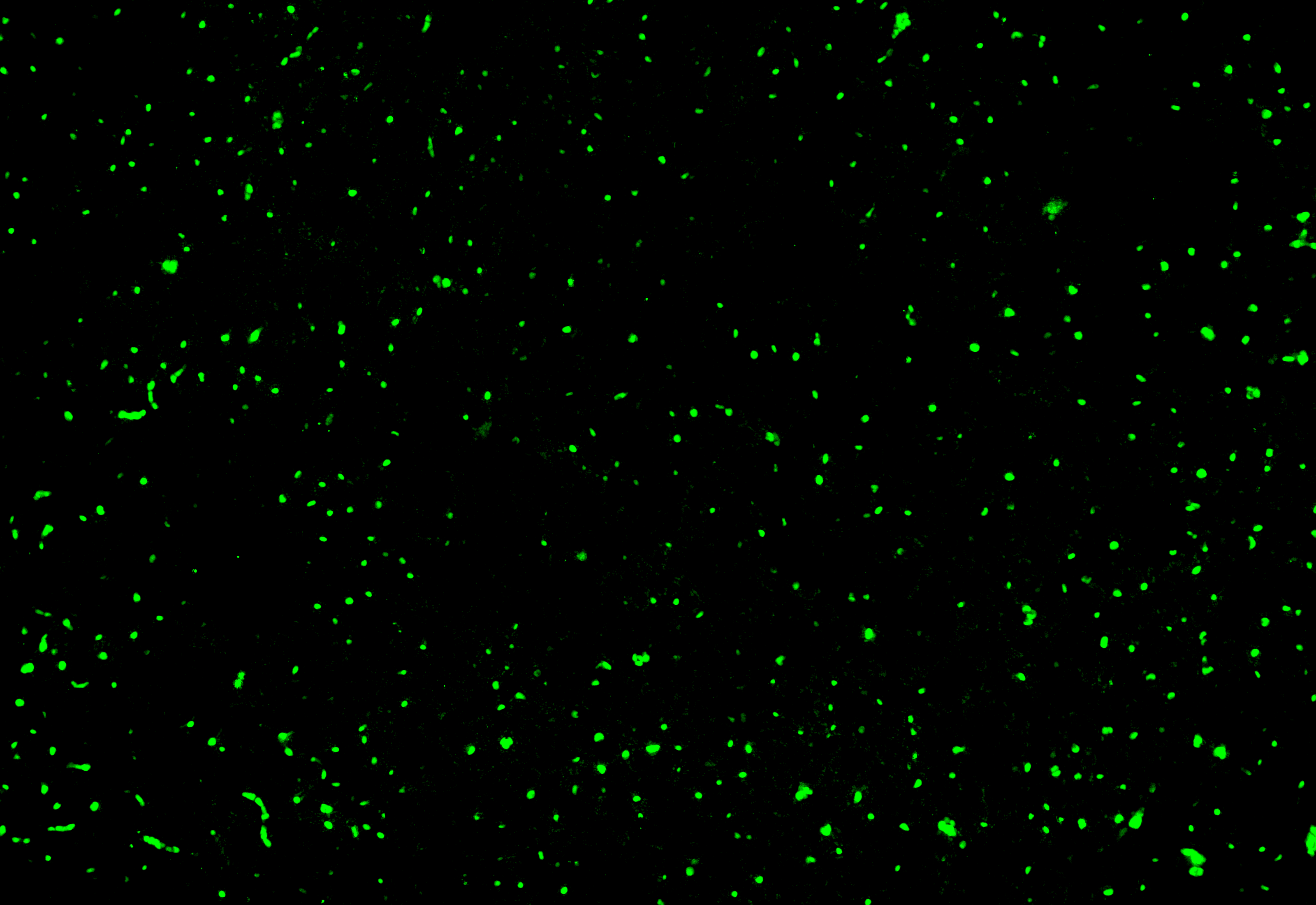

Supplement: Supplemental Material [file KBIE_A_2057632_SM9317.zip › supplementary/Fig4A_IR_Gr_1.tif]

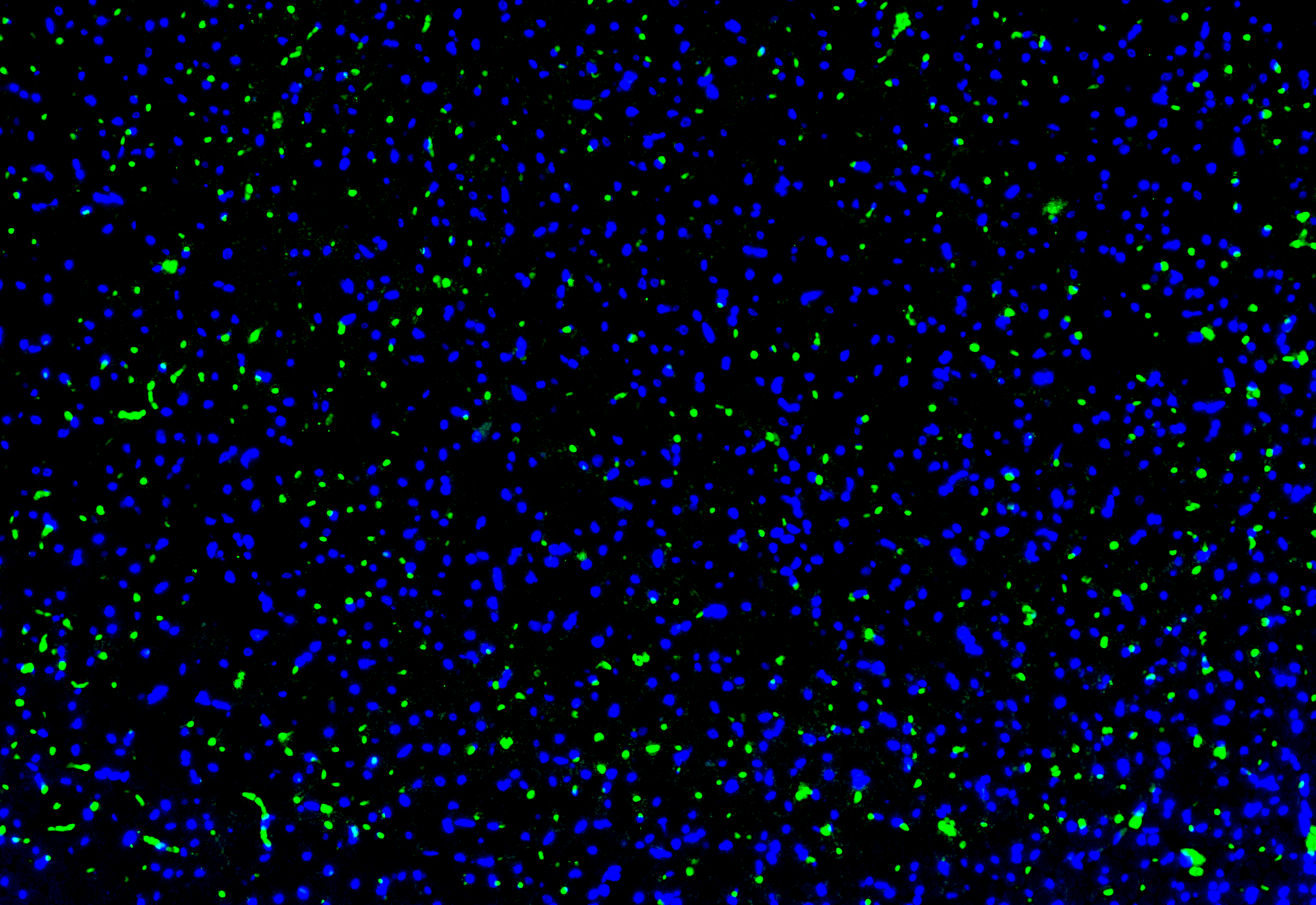

Supplement: Supplemental Material [file KBIE_A_2057632_SM9317.zip › supplementary/Fig4A_IR_Merged.tif]

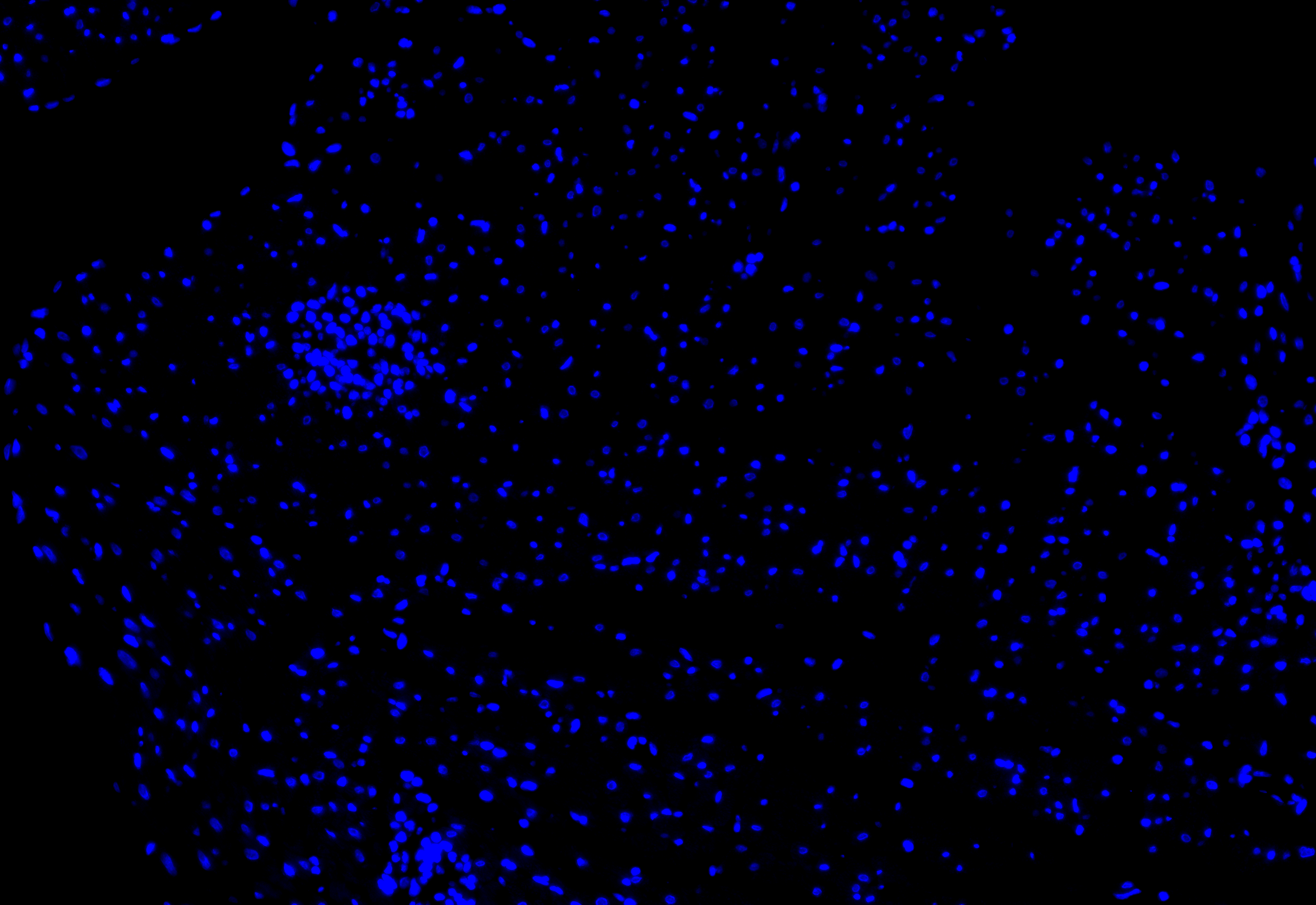

Supplement: Supplemental Material [file KBIE_A_2057632_SM9317.zip › supplementary/Fig4A_IR_Oxycodone_DAPI.tif]

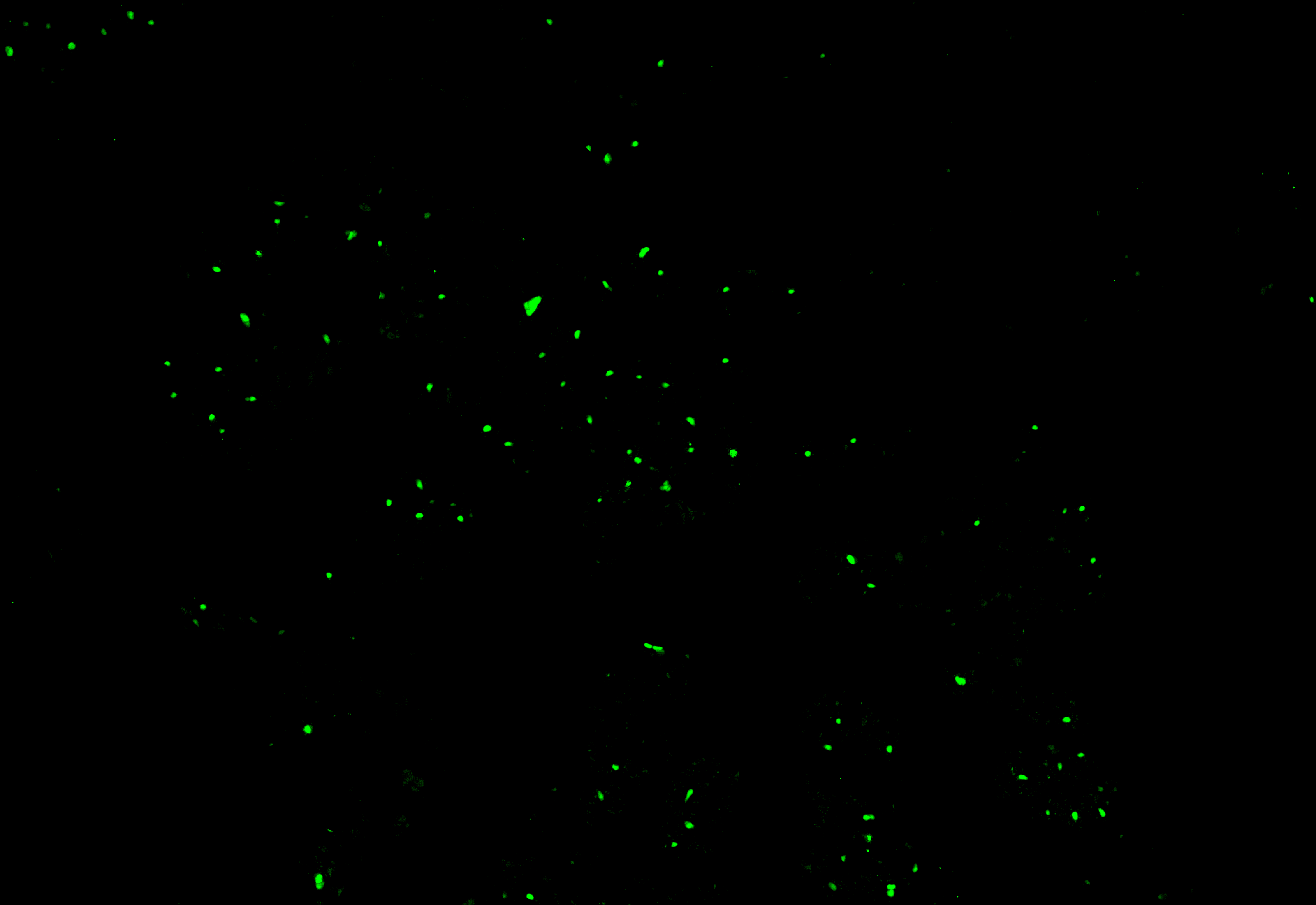

Supplement: Supplemental Material [file KBIE_A_2057632_SM9317.zip › supplementary/Fig4A_IR_Oxycodone_Gr_1.tif]

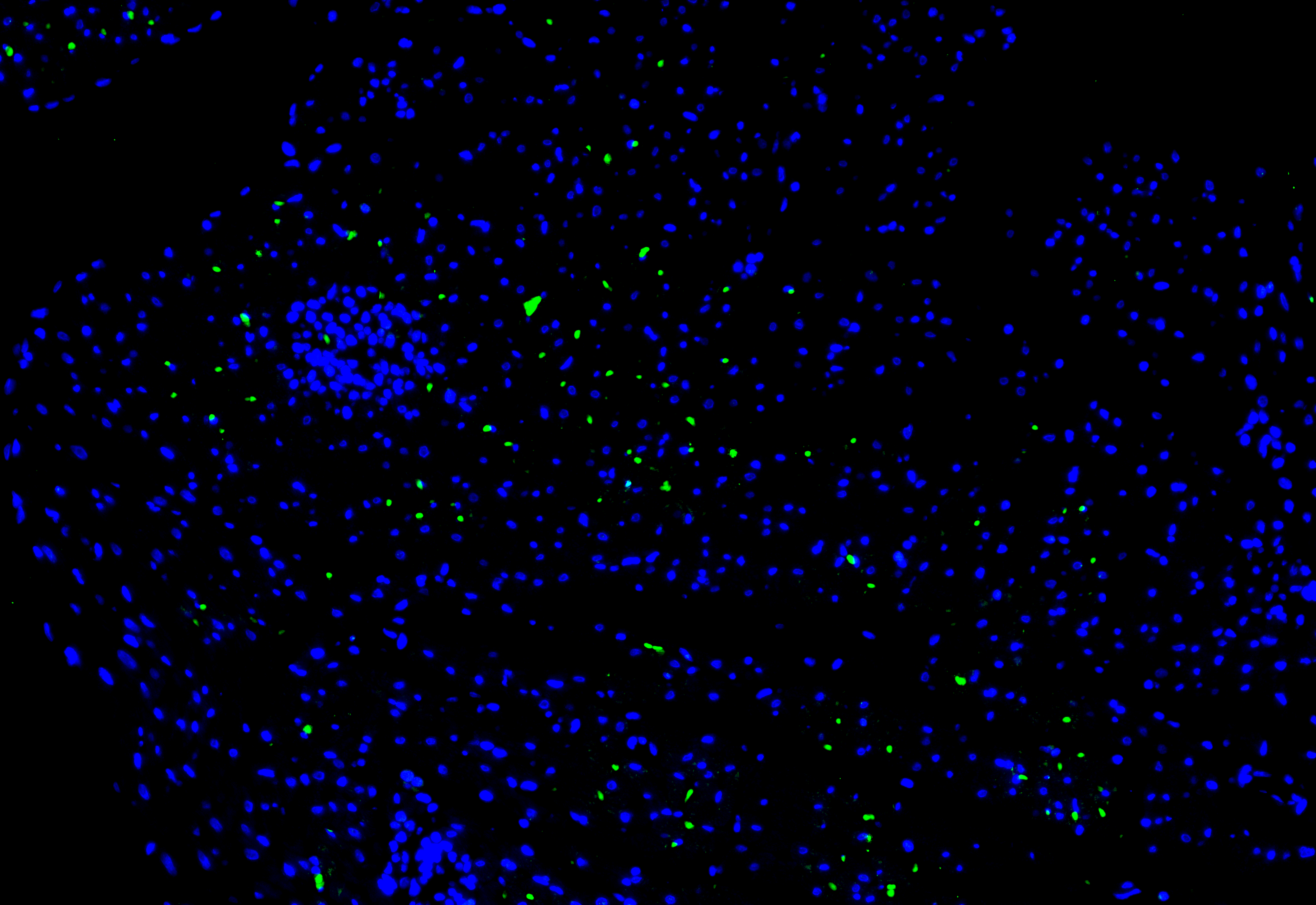

Supplement: Supplemental Material [file KBIE_A_2057632_SM9317.zip › supplementary/Fig4A_IR_Oxycodone_Merged.tif]

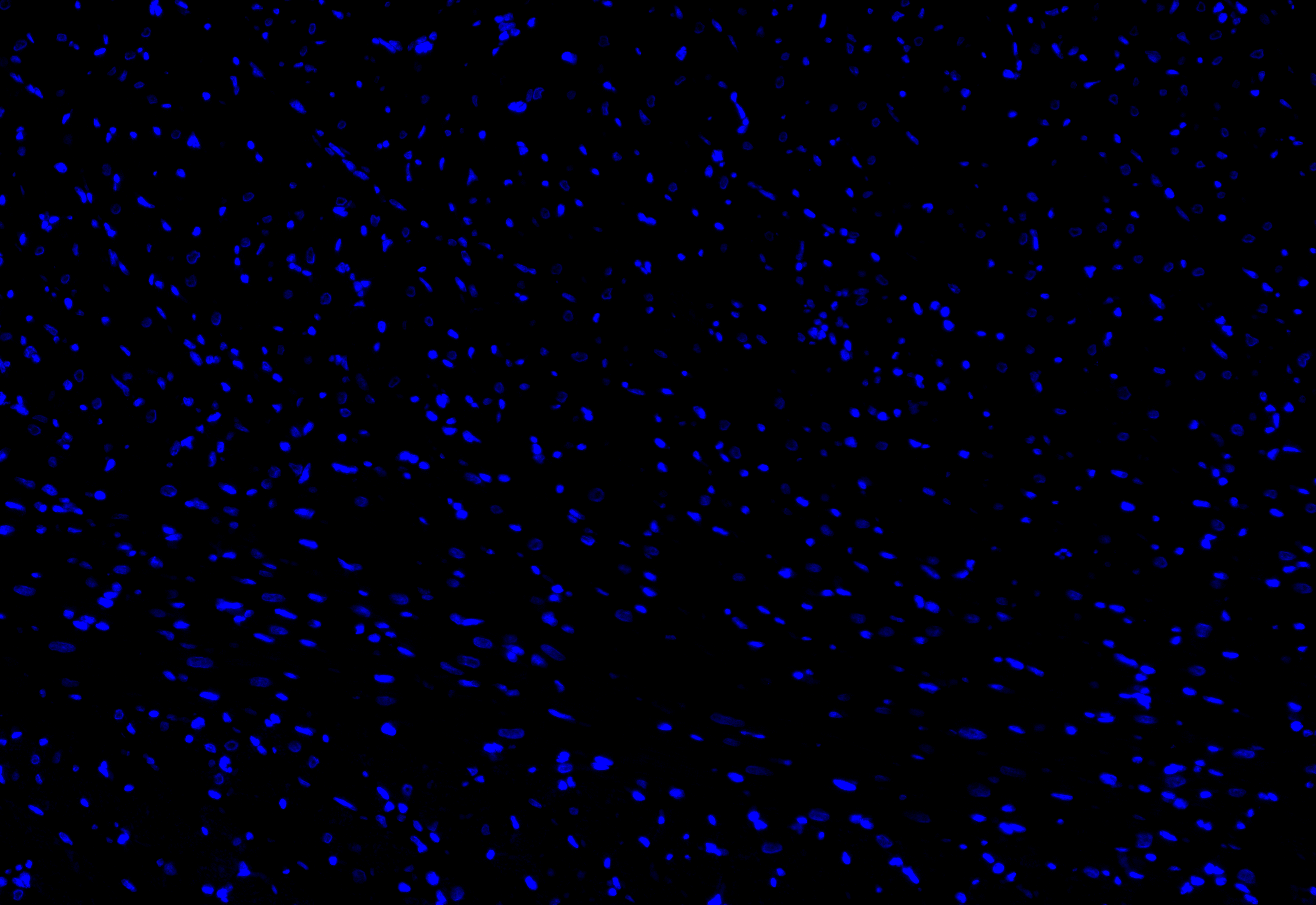

Supplement: Supplemental Material [file KBIE_A_2057632_SM9317.zip › supplementary/Fig4A_Sham_DAPI.tif]

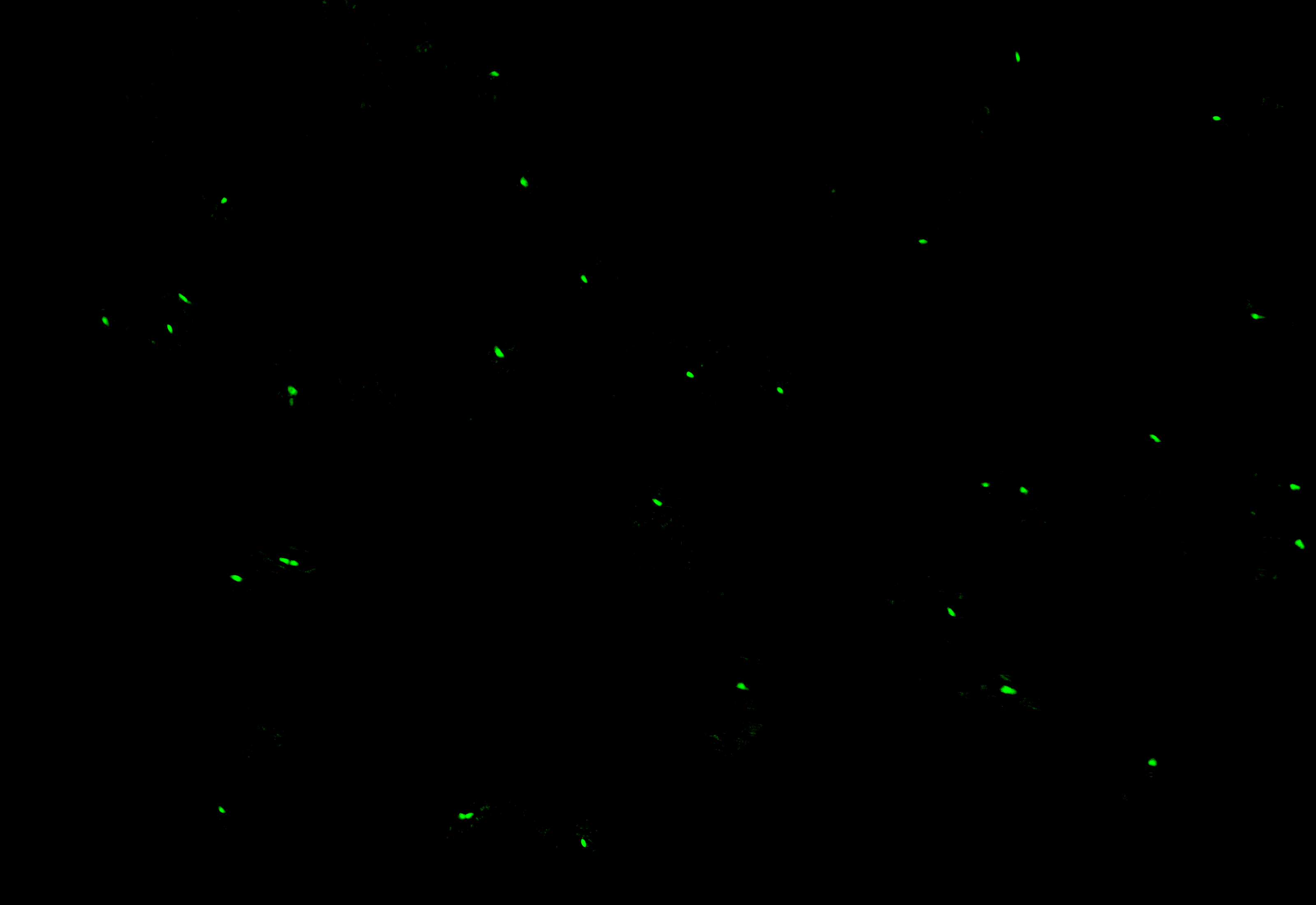

Supplement: Supplemental Material [file KBIE_A_2057632_SM9317.zip › supplementary/Fig4A_Sham_Gr_1.tif]

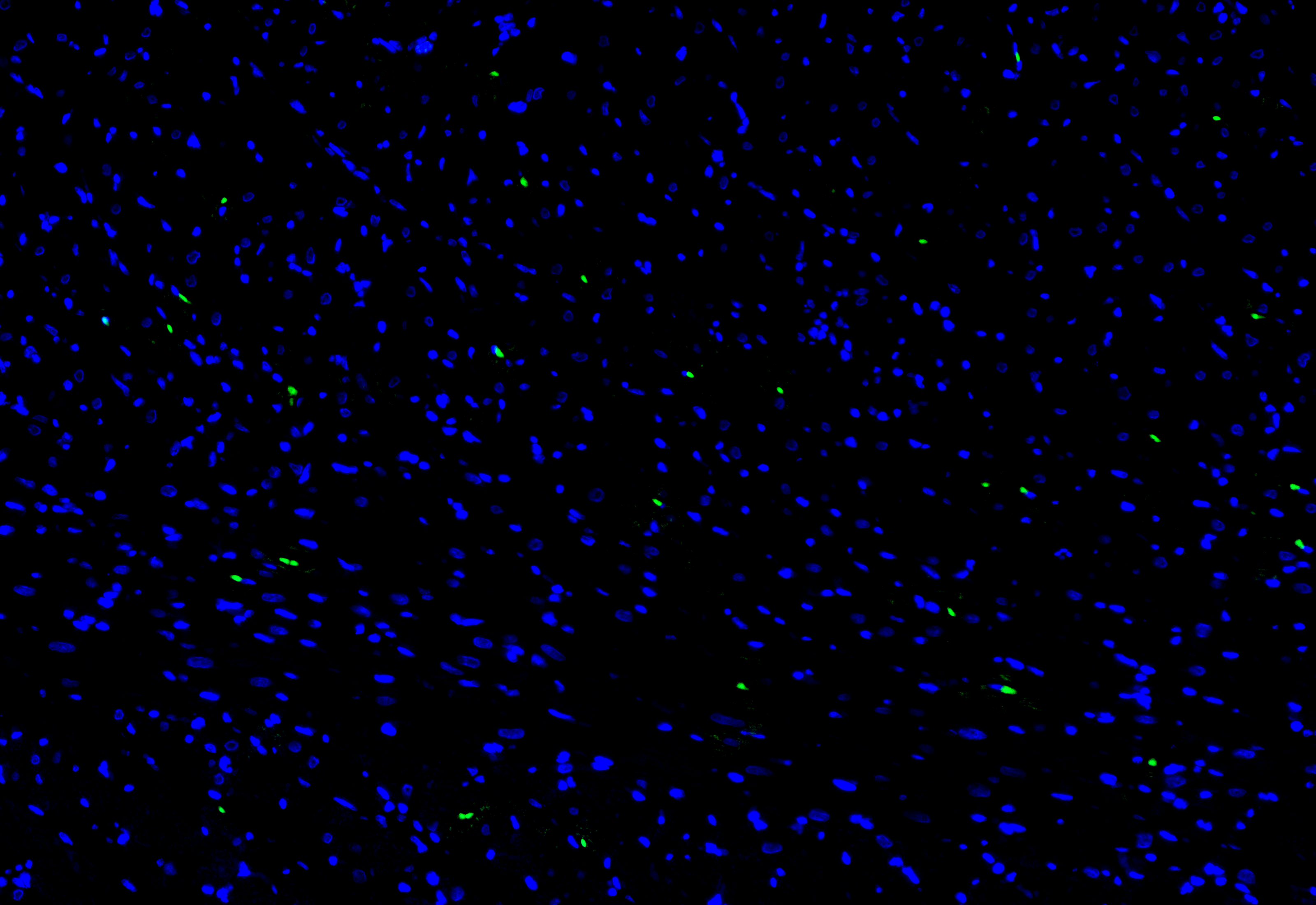

Supplement: Supplemental Material [file KBIE_A_2057632_SM9317.zip › supplementary/Fig4A_Sham_Merged.tif]

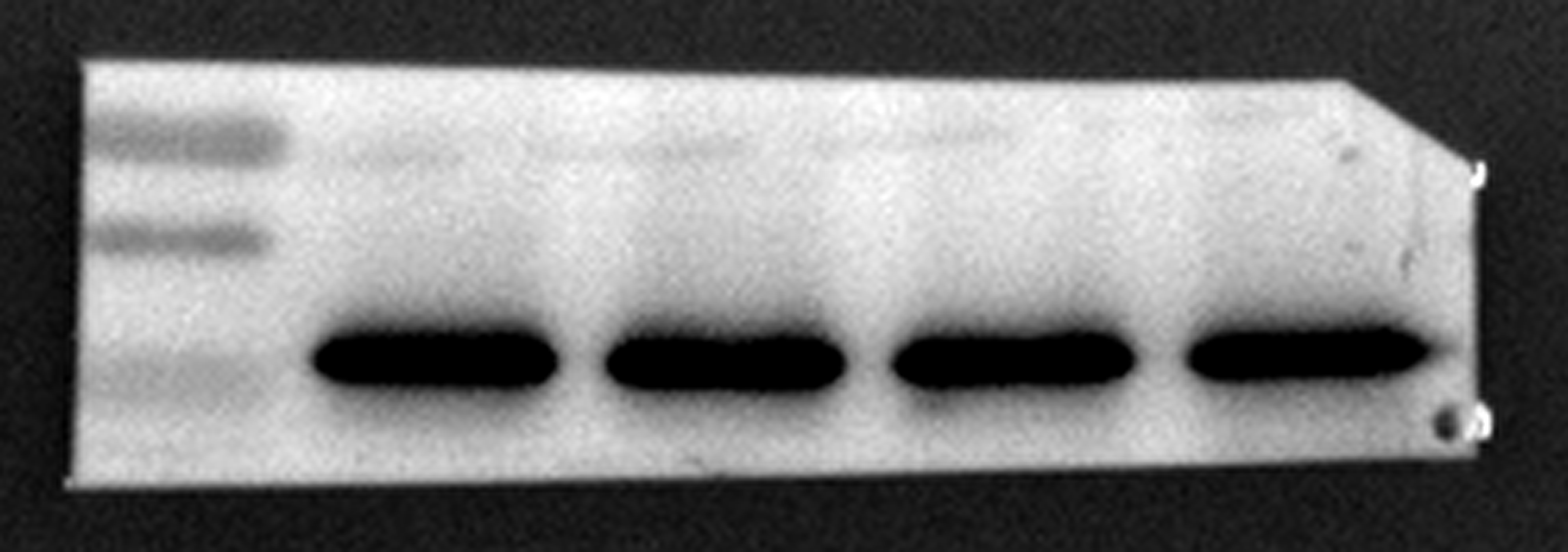

Supplement: Supplemental Material [file KBIE_A_2057632_SM9317.zip › supplementary/Fig4C_GAPDH.tif]

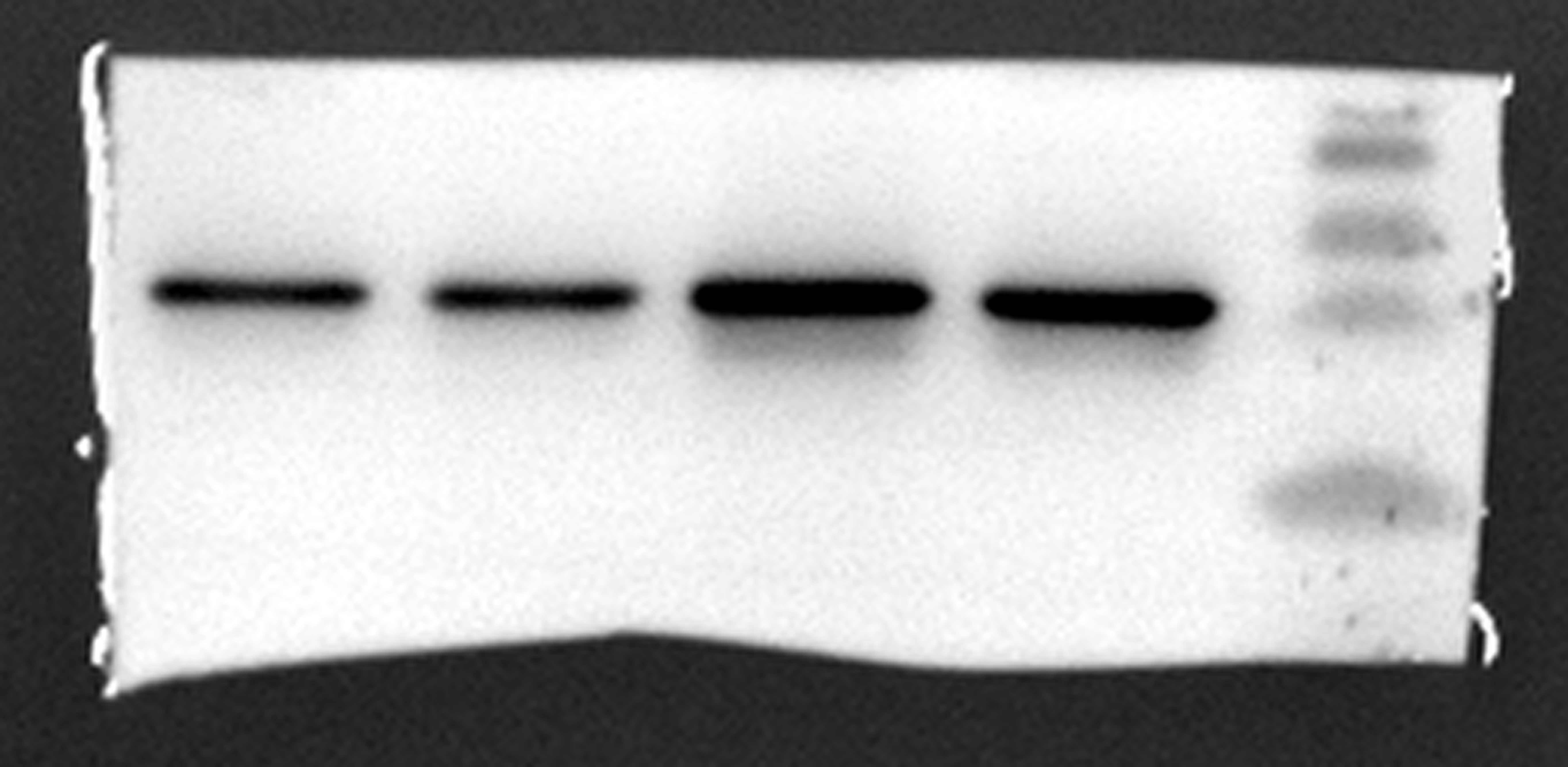

Supplement: Supplemental Material [file KBIE_A_2057632_SM9317.zip › supplementary/Fig4C_MCP_1.tif]

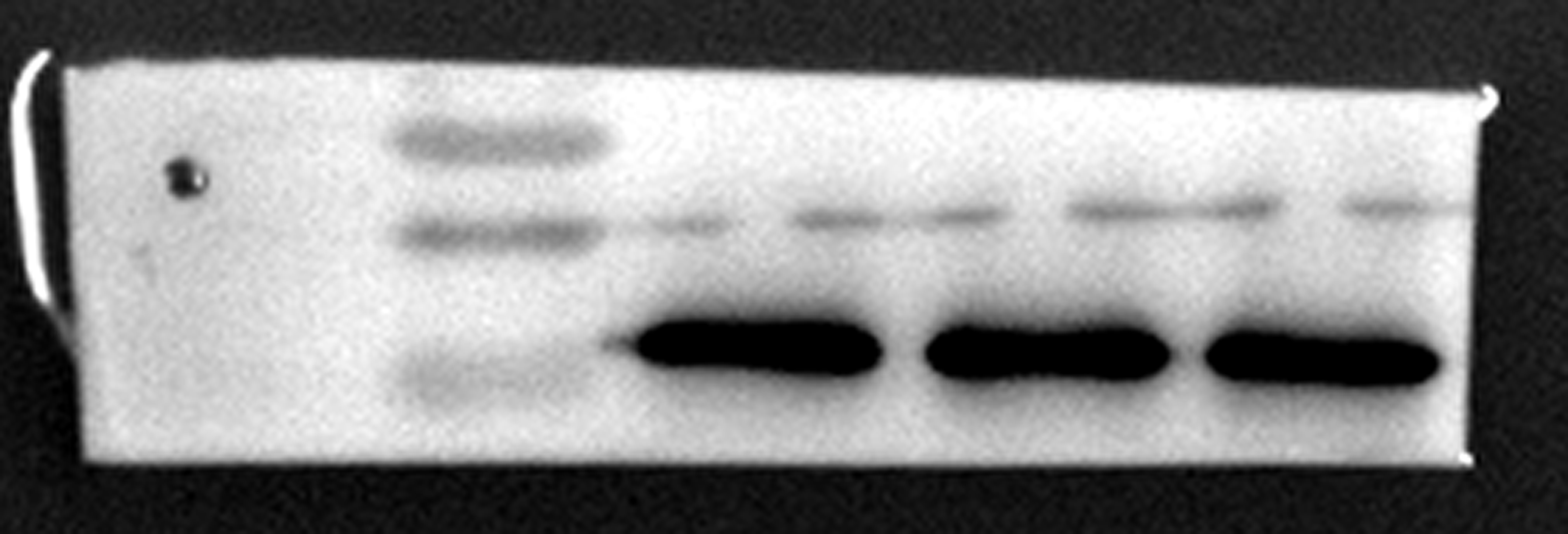

Supplement: Supplemental Material [file KBIE_A_2057632_SM9317.zip › supplementary/Fig5C_GAPDH.tif]

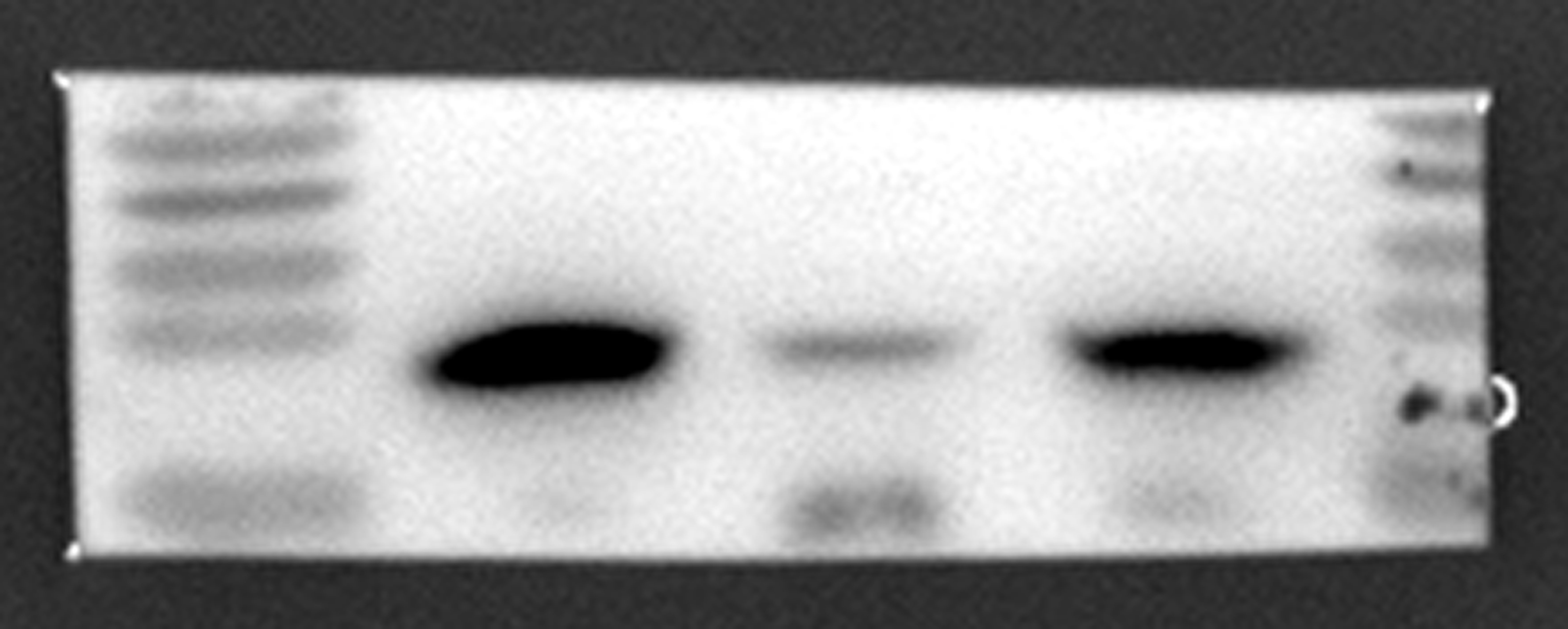

Supplement: Supplemental Material [file KBIE_A_2057632_SM9317.zip › supplementary/Fig5C_SIGMAR1.tif]

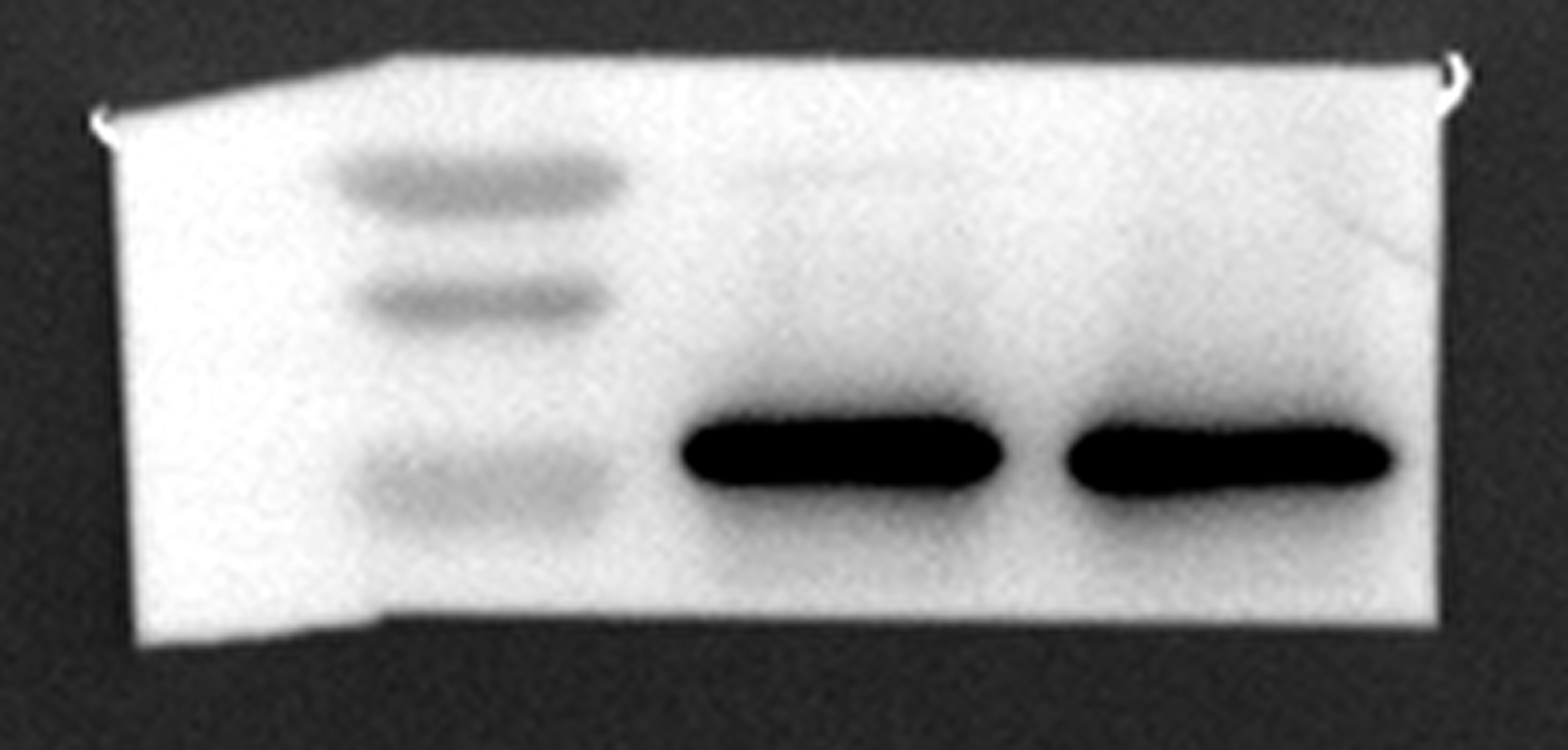

Supplement: Supplemental Material [file KBIE_A_2057632_SM9317.zip › supplementary/Fig5E_GAPDH.tif]

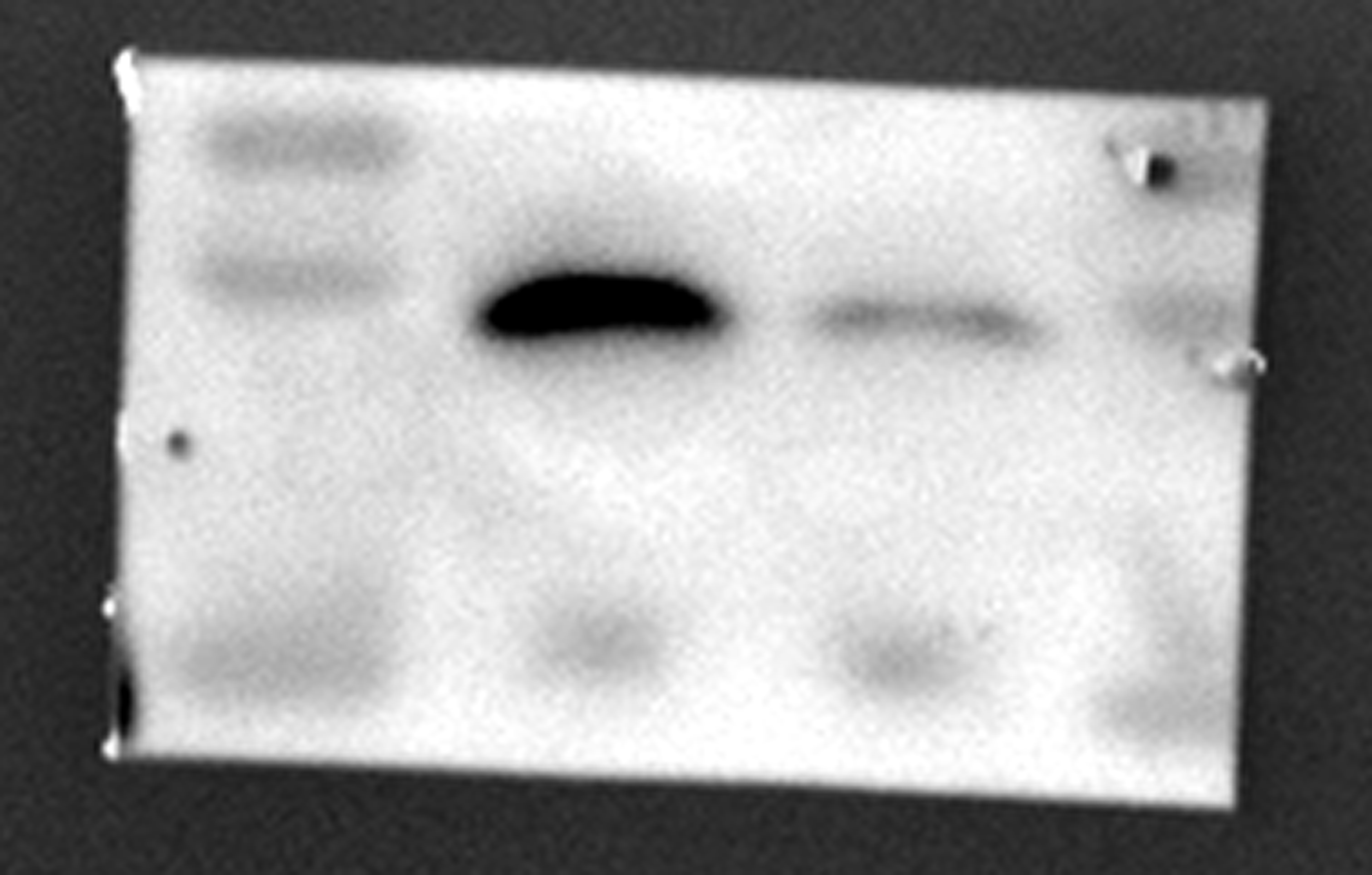

Supplement: Supplemental Material [file KBIE_A_2057632_SM9317.zip › supplementary/Fig5E_SIGMAR1.tif]

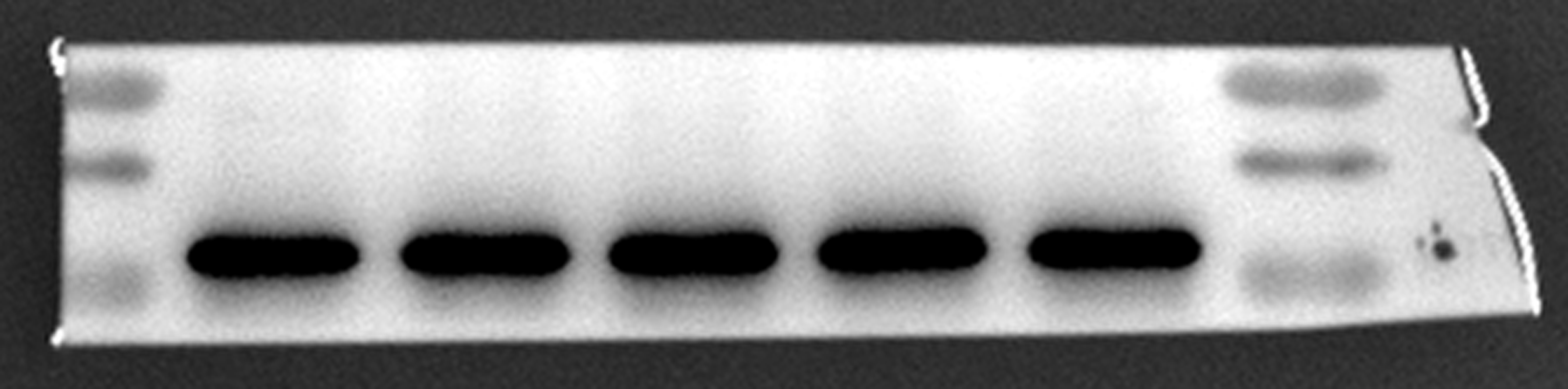

Supplement: Supplemental Material [file KBIE_A_2057632_SM9317.zip › supplementary/Fig5G_GAPDH.tif]

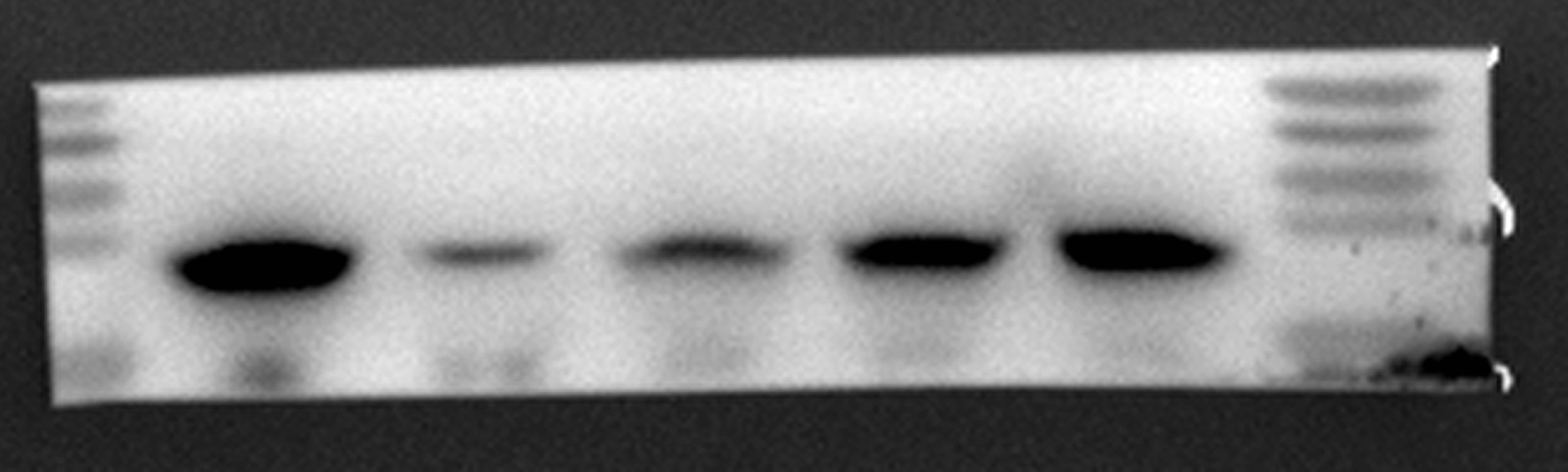

Supplement: Supplemental Material [file KBIE_A_2057632_SM9317.zip › supplementary/Fig5G_SIGMAR1.tif]

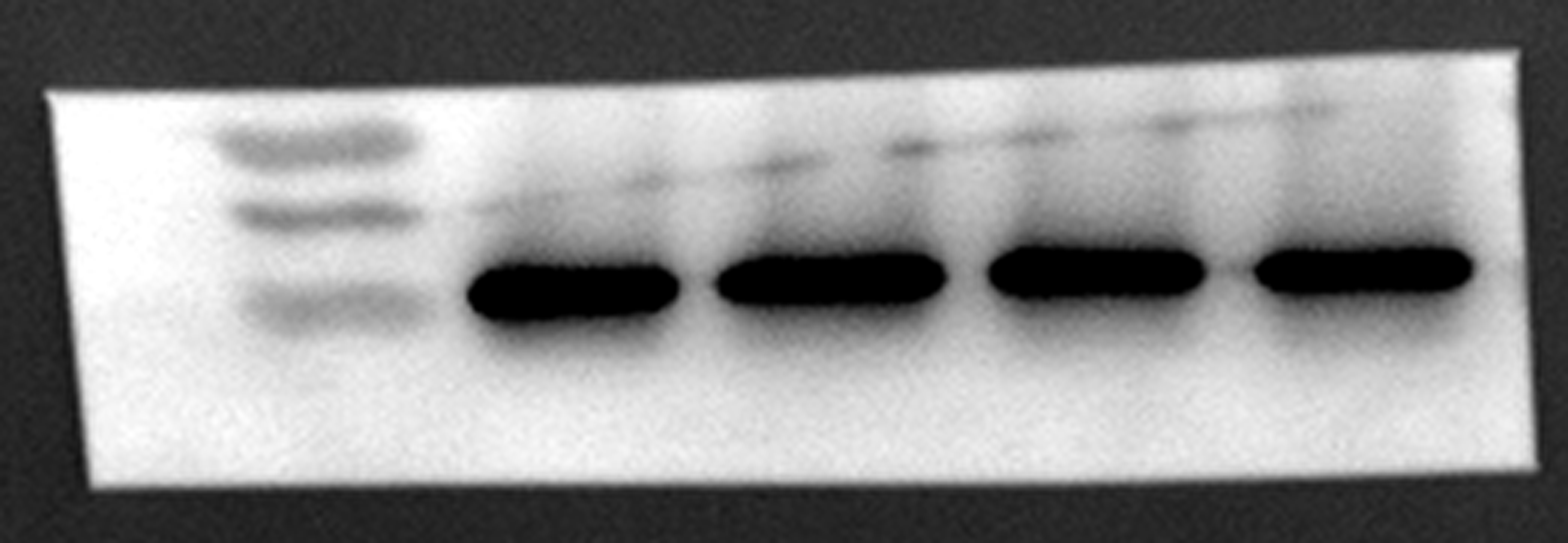

Supplement: Supplemental Material [file KBIE_A_2057632_SM9317.zip › supplementary/Fig6B_GAPDH.tif]

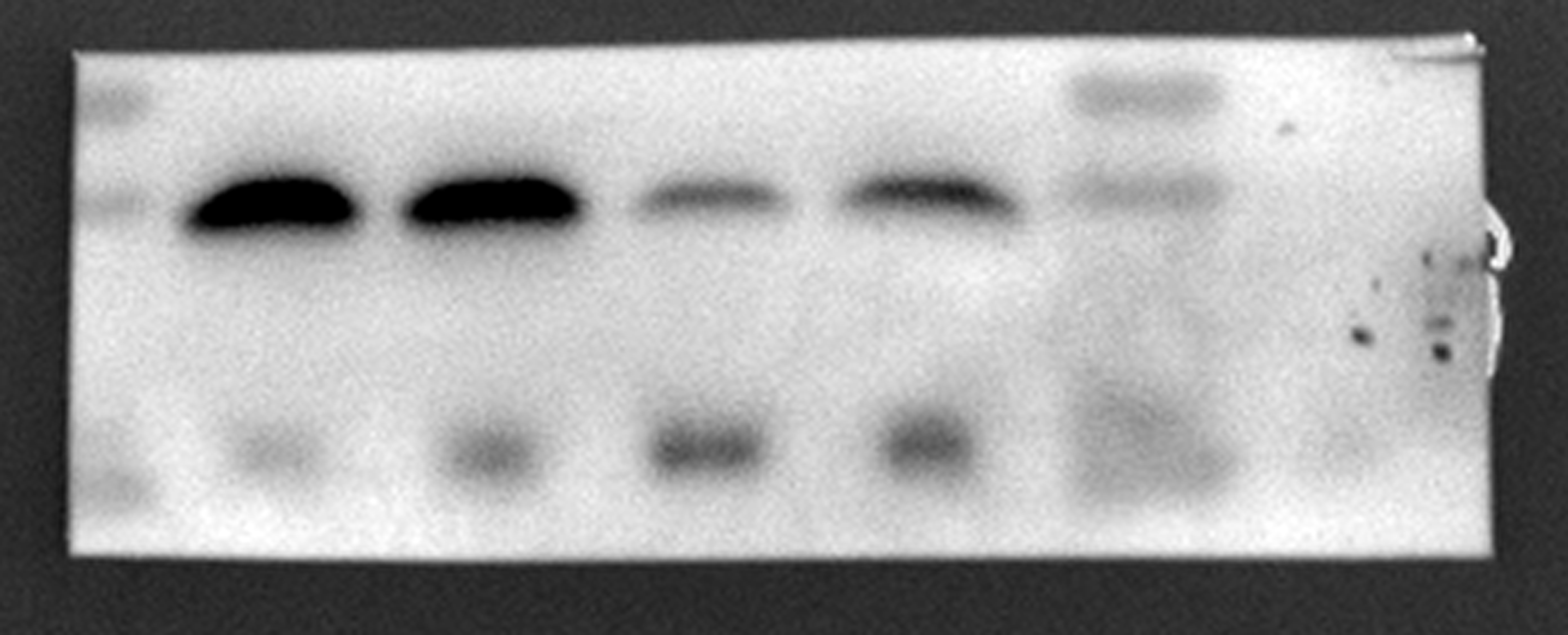

Supplement: Supplemental Material [file KBIE_A_2057632_SM9317.zip › supplementary/Fig6B_SIGMAR1.tif]

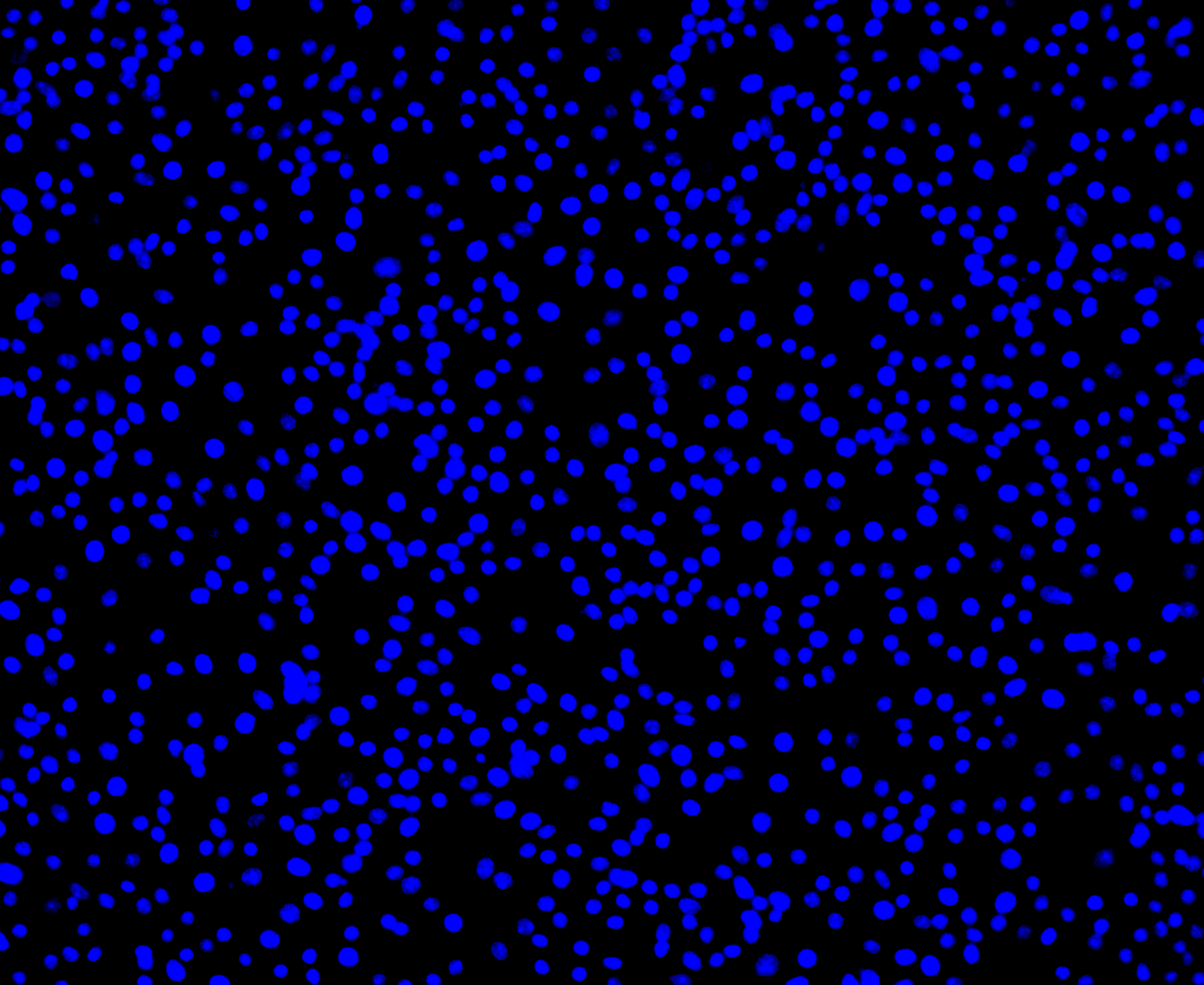

Supplement: Supplemental Material [file KBIE_A_2057632_SM9317.zip › supplementary/Fig6D_Control_DAPI.tif]

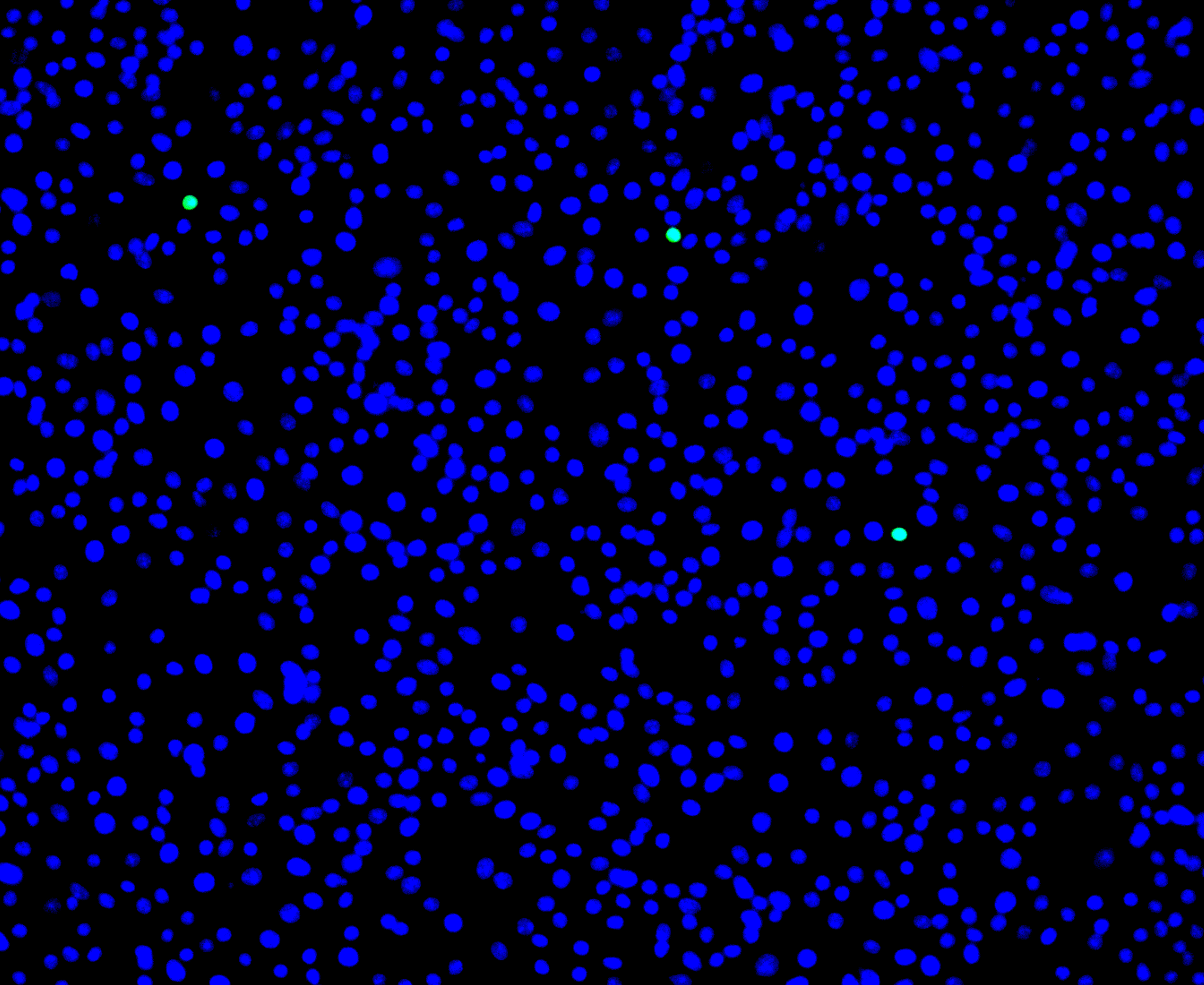

Supplement: Supplemental Material [file KBIE_A_2057632_SM9317.zip › supplementary/Fig6D_Control_Merged.tif]

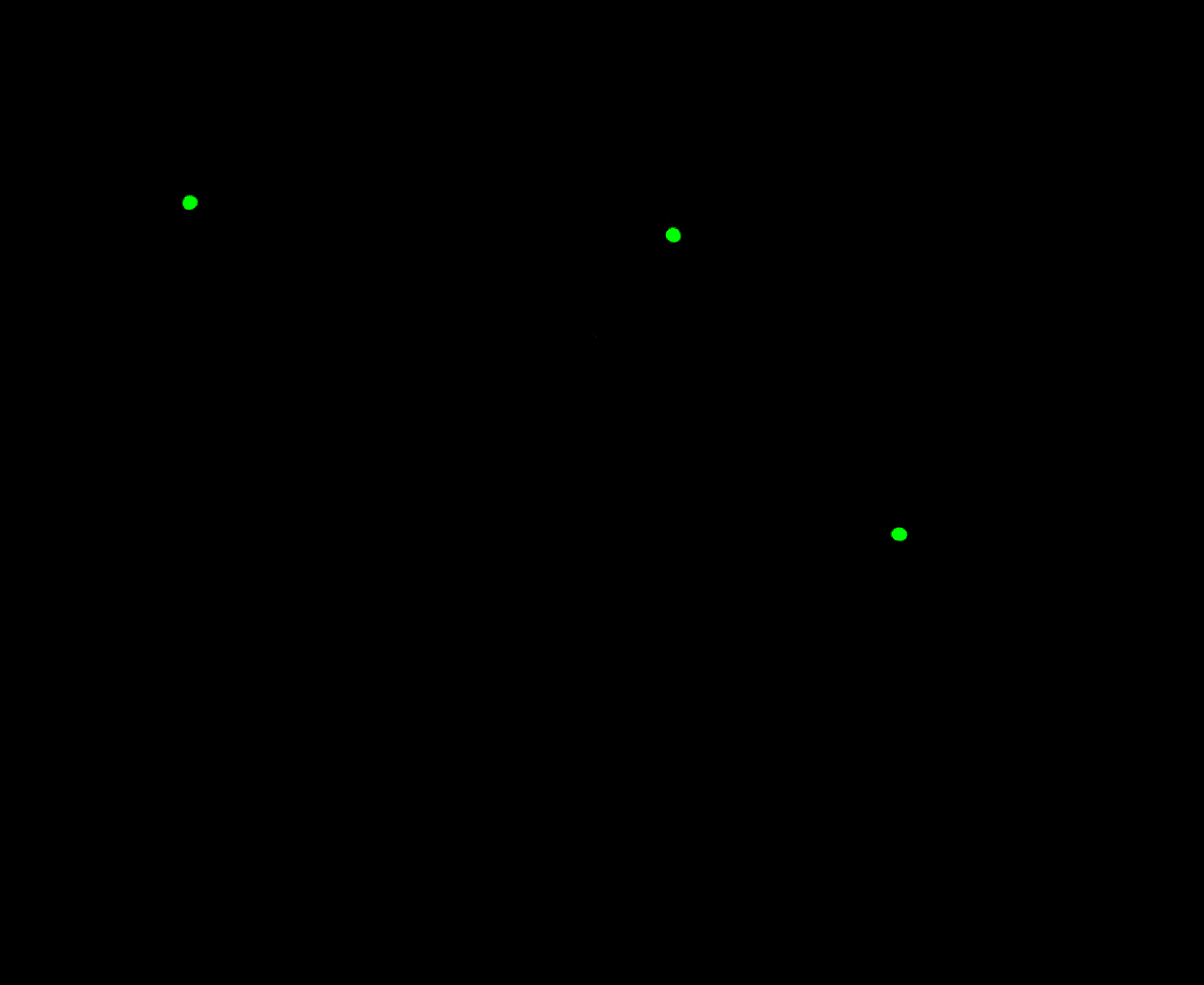

Supplement: Supplemental Material [file KBIE_A_2057632_SM9317.zip › supplementary/Fig6D_Control_Tunel.tif]

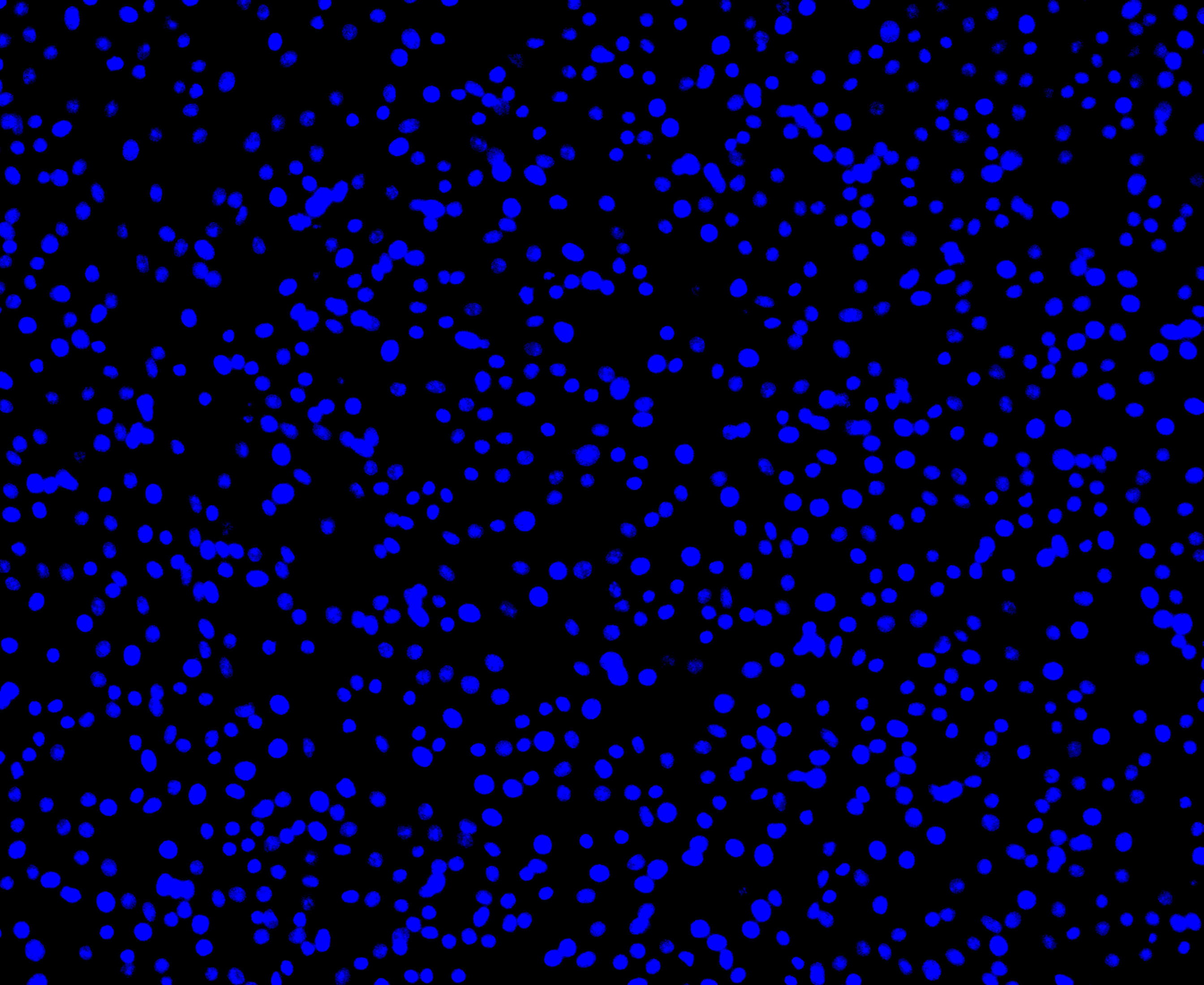

Supplement: Supplemental Material [file KBIE_A_2057632_SM9317.zip › supplementary/Fig6D_HR_1_5 ngmL_Oxycodone_BD1047_DAPI.tif]

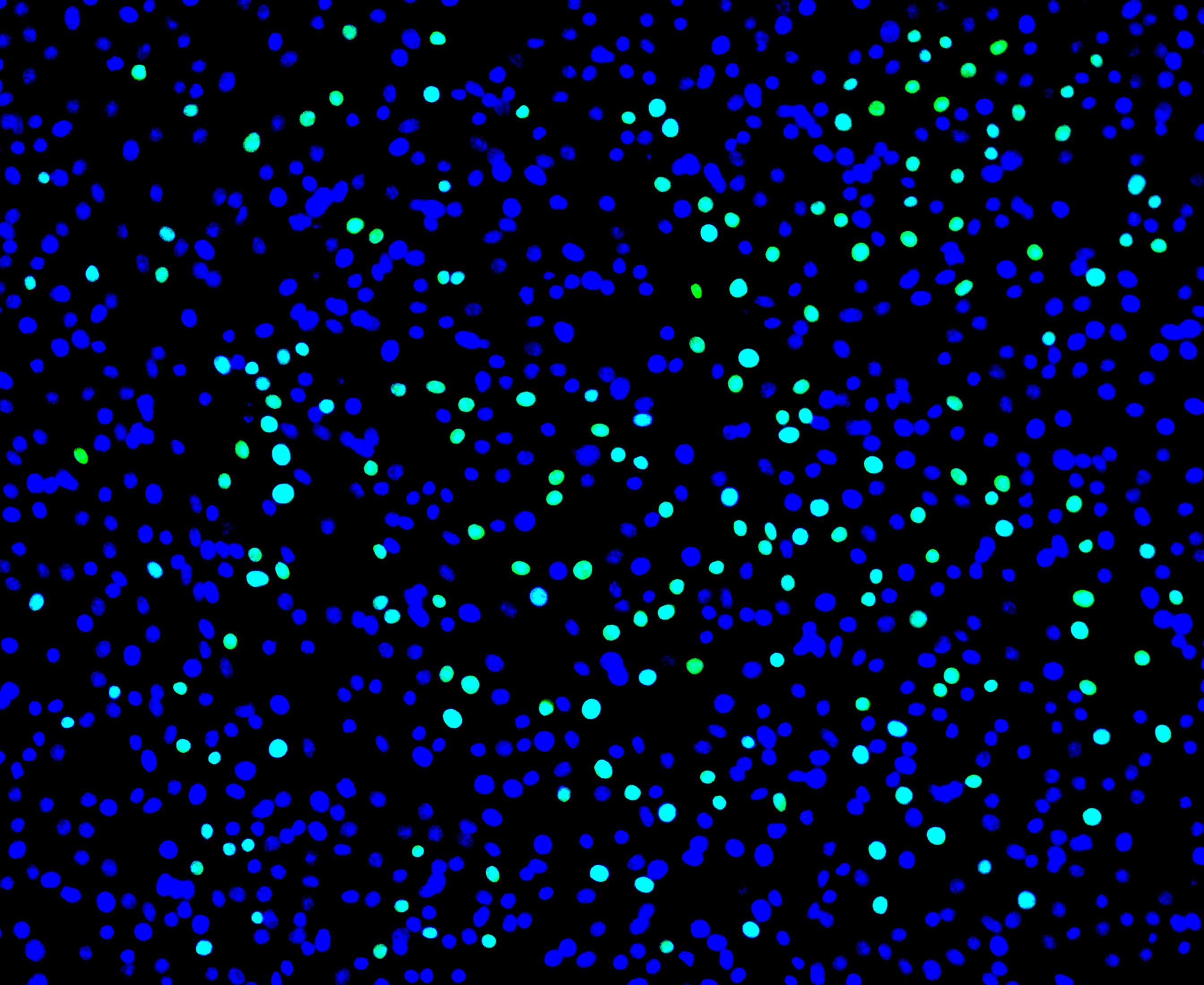

Supplement: Supplemental Material [file KBIE_A_2057632_SM9317.zip › supplementary/Fig6D_HR_1_5 ngmL_Oxycodone_BD1047_Merged.tif]

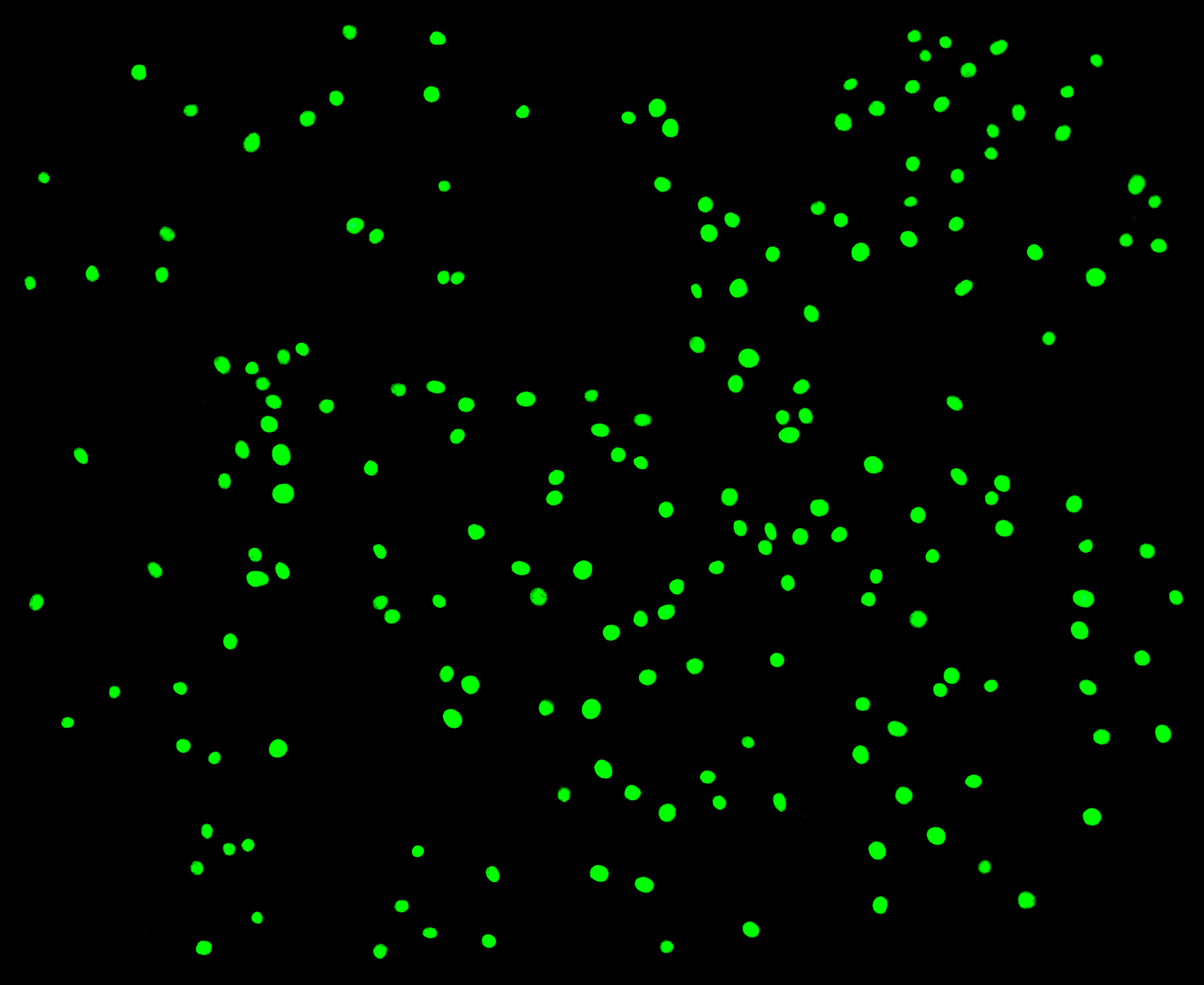

Supplement: Supplemental Material [file KBIE_A_2057632_SM9317.zip › supplementary/Fig6D_HR_1_5 ngmL_Oxycodone_BD1047_Tunel.tif]

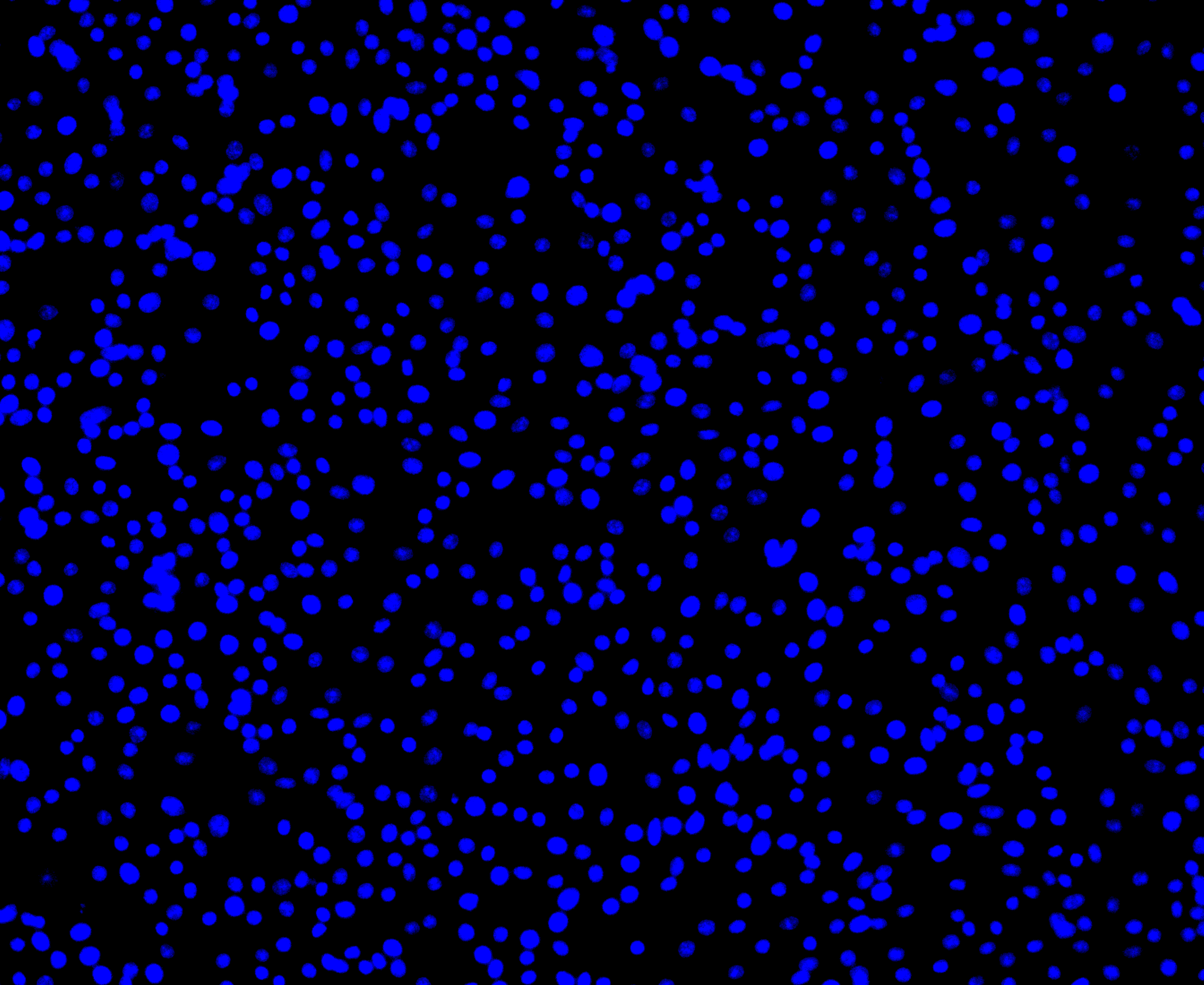

Supplement: Supplemental Material [file KBIE_A_2057632_SM9317.zip › supplementary/Fig6D_HR_1_5 ngmL_Oxycodone_DAPI.tif]

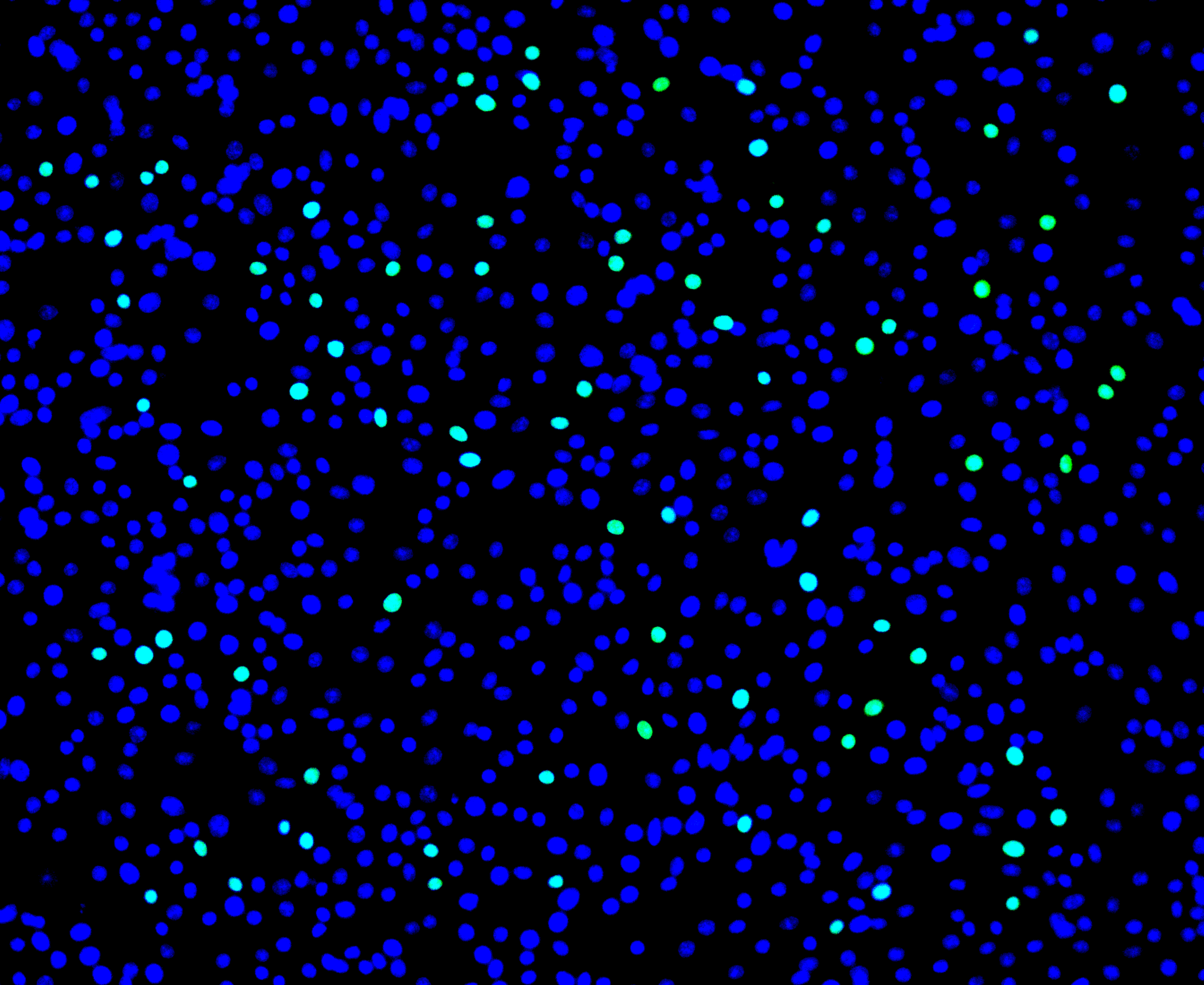

Supplement: Supplemental Material [file KBIE_A_2057632_SM9317.zip › supplementary/Fig6D_HR_1_5 ngmL_Oxycodone_Merged.tif]

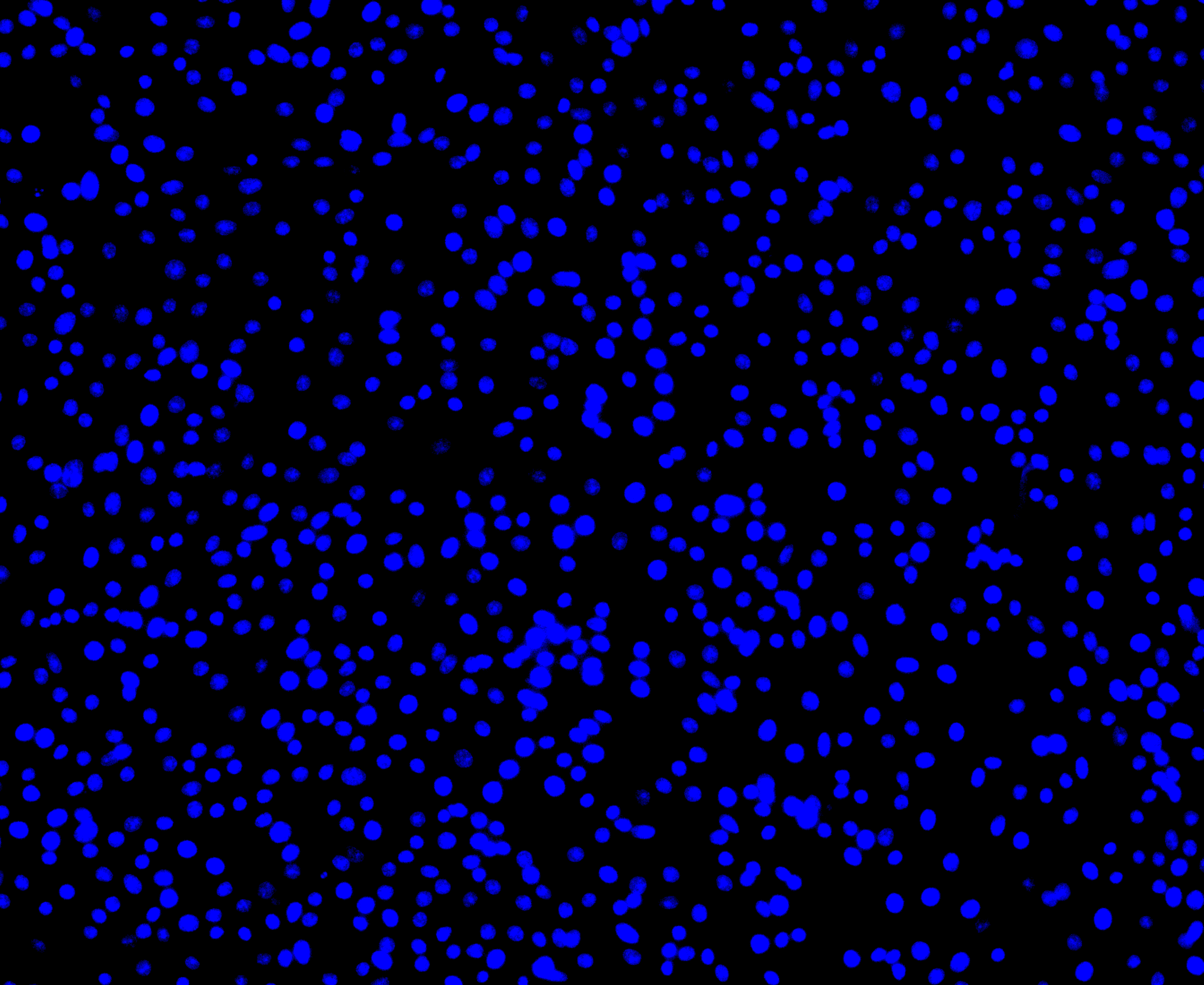

Supplement: Supplemental Material [file KBIE_A_2057632_SM9317.zip › supplementary/Fig6D_HR_1_5 ngmL_Oxycodone_shRNA_NC_DAPI.tif]

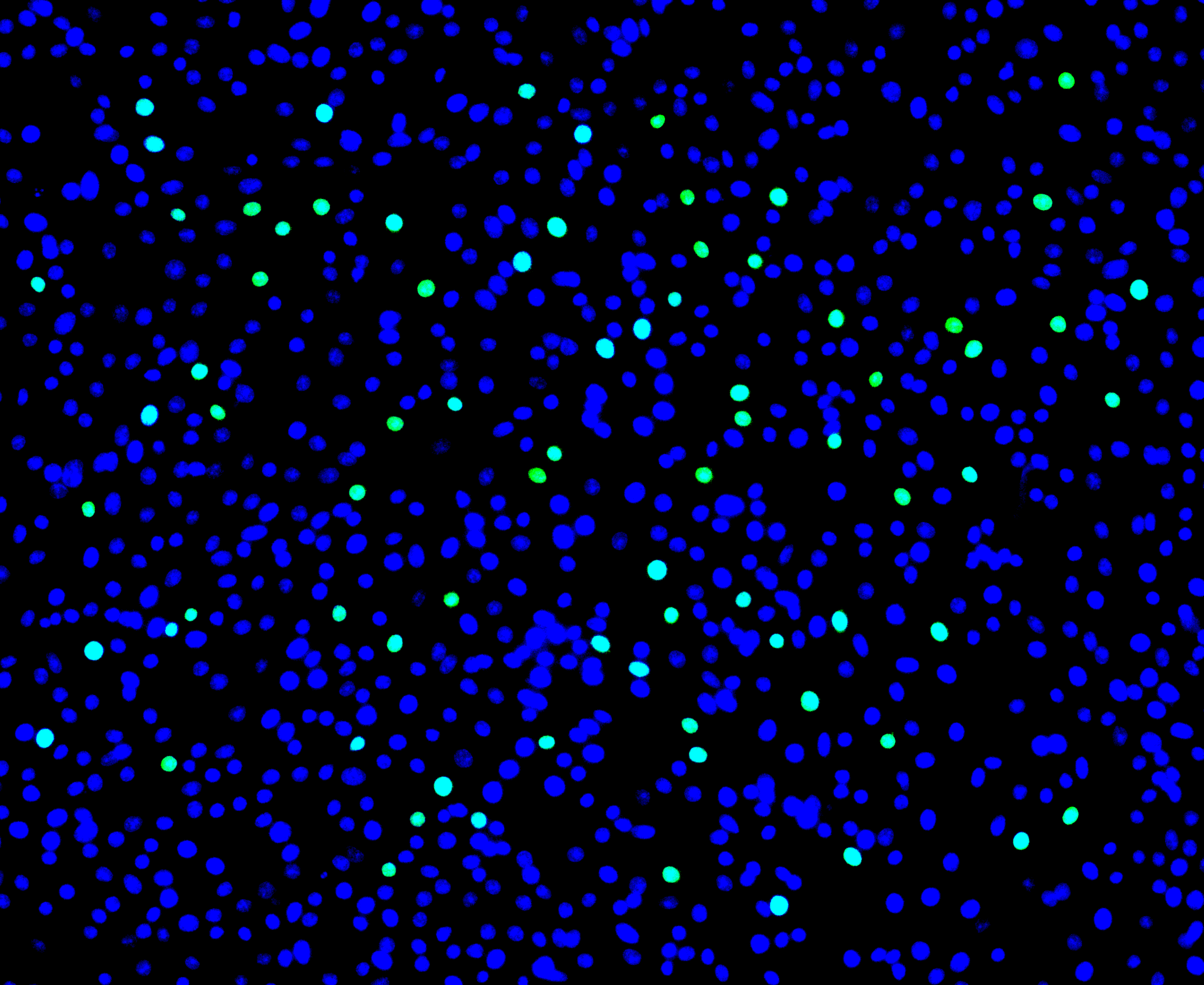

Supplement: Supplemental Material [file KBIE_A_2057632_SM9317.zip › supplementary/Fig6D_HR_1_5 ngmL_Oxycodone_shRNA_NC_Merged.tif]

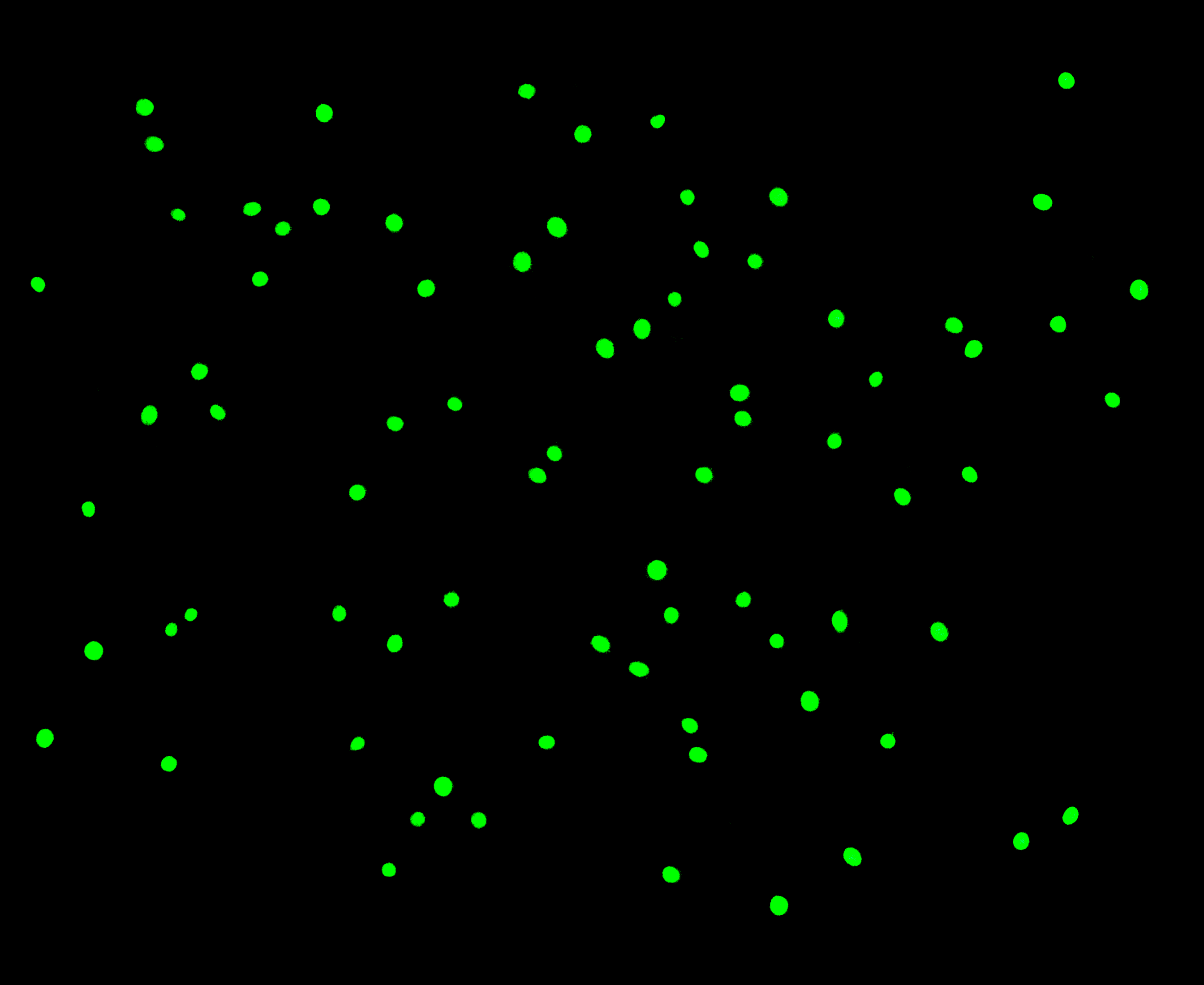

Supplement: Supplemental Material [file KBIE_A_2057632_SM9317.zip › supplementary/Fig6D_HR_1_5 ngmL_Oxycodone_shRNA_NC_Tunel.tif]

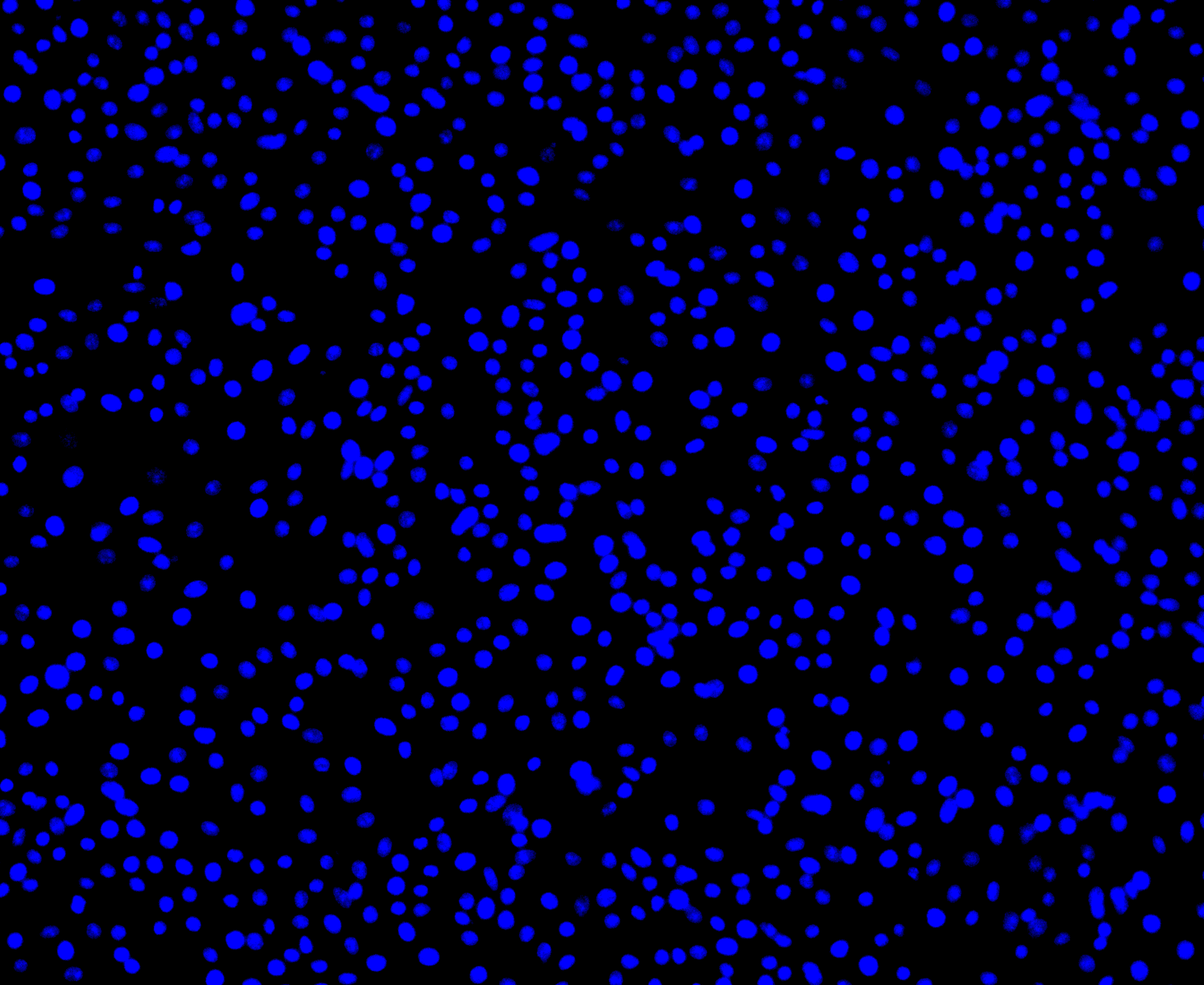

Supplement: Supplemental Material [file KBIE_A_2057632_SM9317.zip › supplementary/Fig6D_HR_1_5 ngmL_Oxycodone_shRNA_SIGMAR1_DAPI.tif]

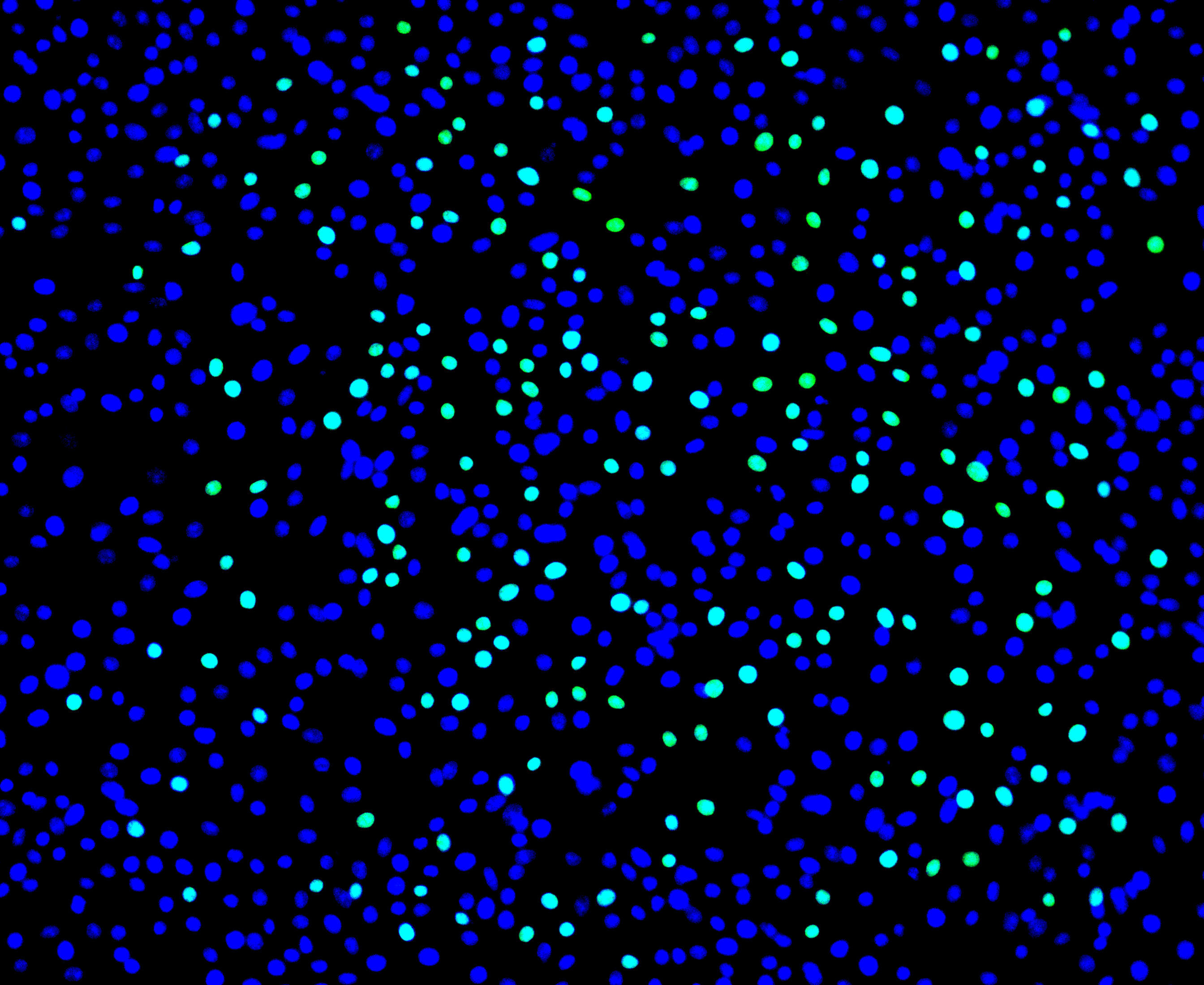

Supplement: Supplemental Material [file KBIE_A_2057632_SM9317.zip › supplementary/Fig6D_HR_1_5 ngmL_Oxycodone_shRNA_SIGMAR1_Merged.tif]

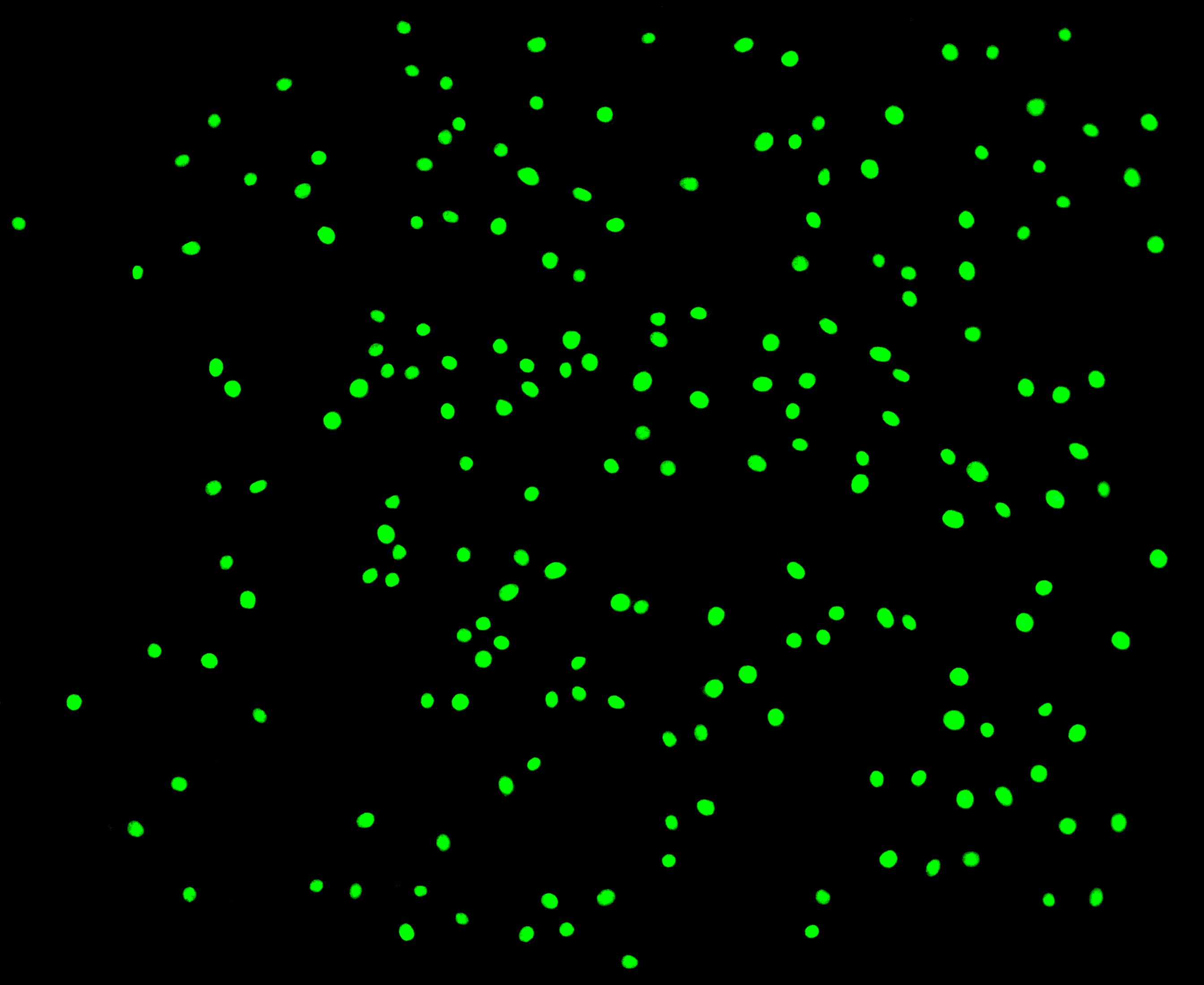

Supplement: Supplemental Material [file KBIE_A_2057632_SM9317.zip › supplementary/Fig6D_HR_1_5 ngmL_Oxycodone_shRNA_SIGMAR1_Tunel.tif]

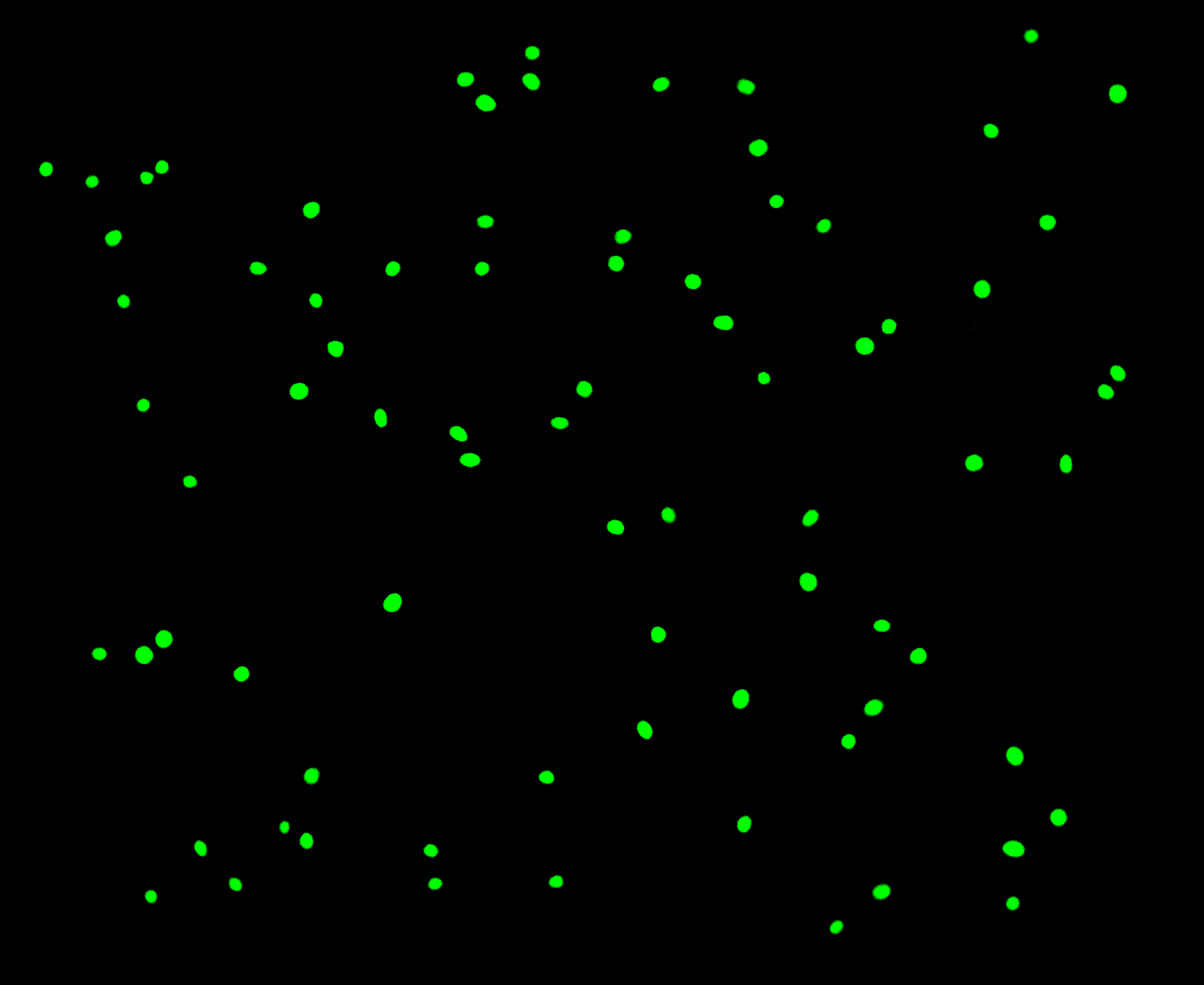

Supplement: Supplemental Material [file KBIE_A_2057632_SM9317.zip › supplementary/Fig6D_HR_1_5 ngmL_Oxycodone_Tunel.tif]

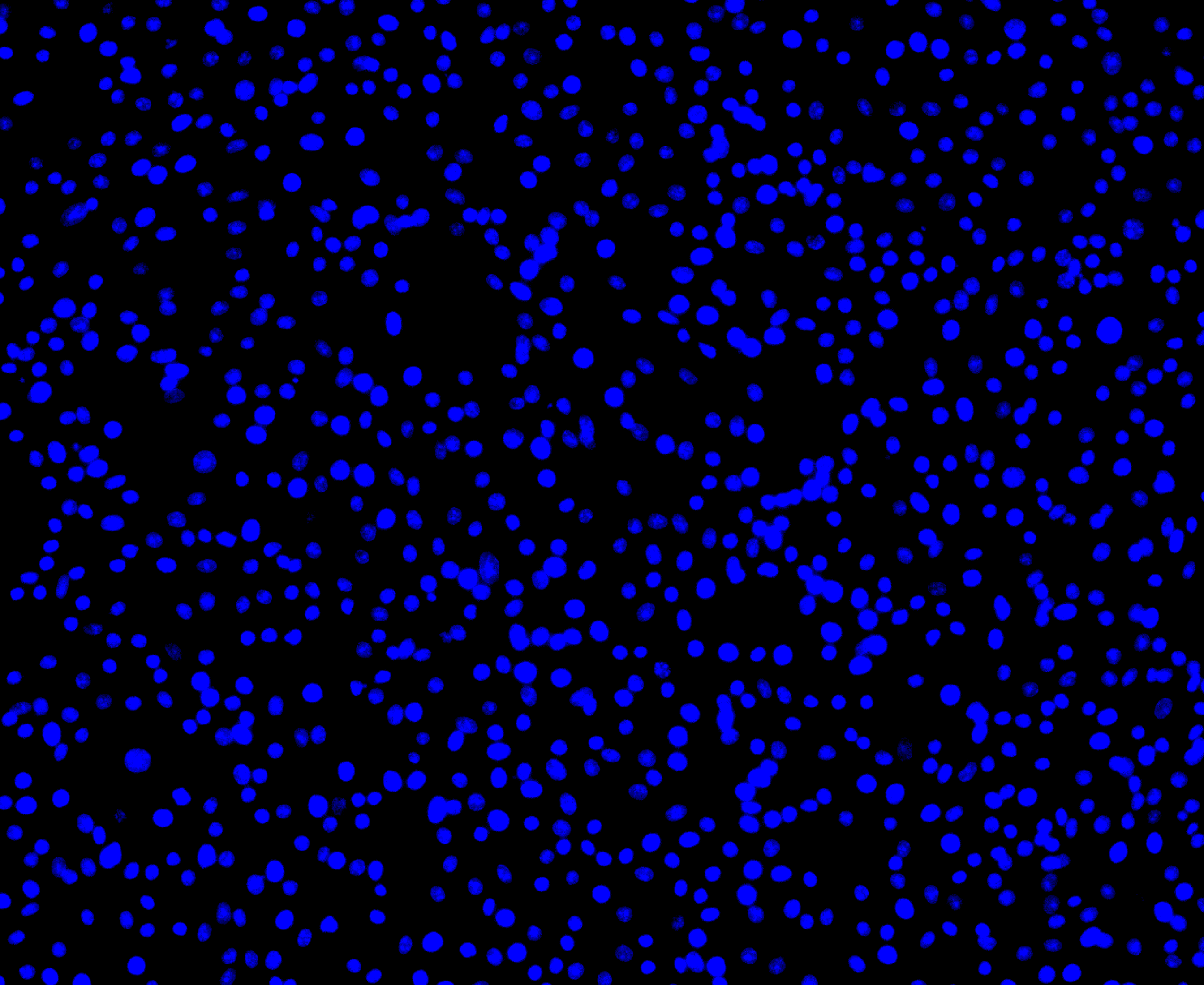

Supplement: Supplemental Material [file KBIE_A_2057632_SM9317.zip › supplementary/Fig6D_HR_DAPI.tif]

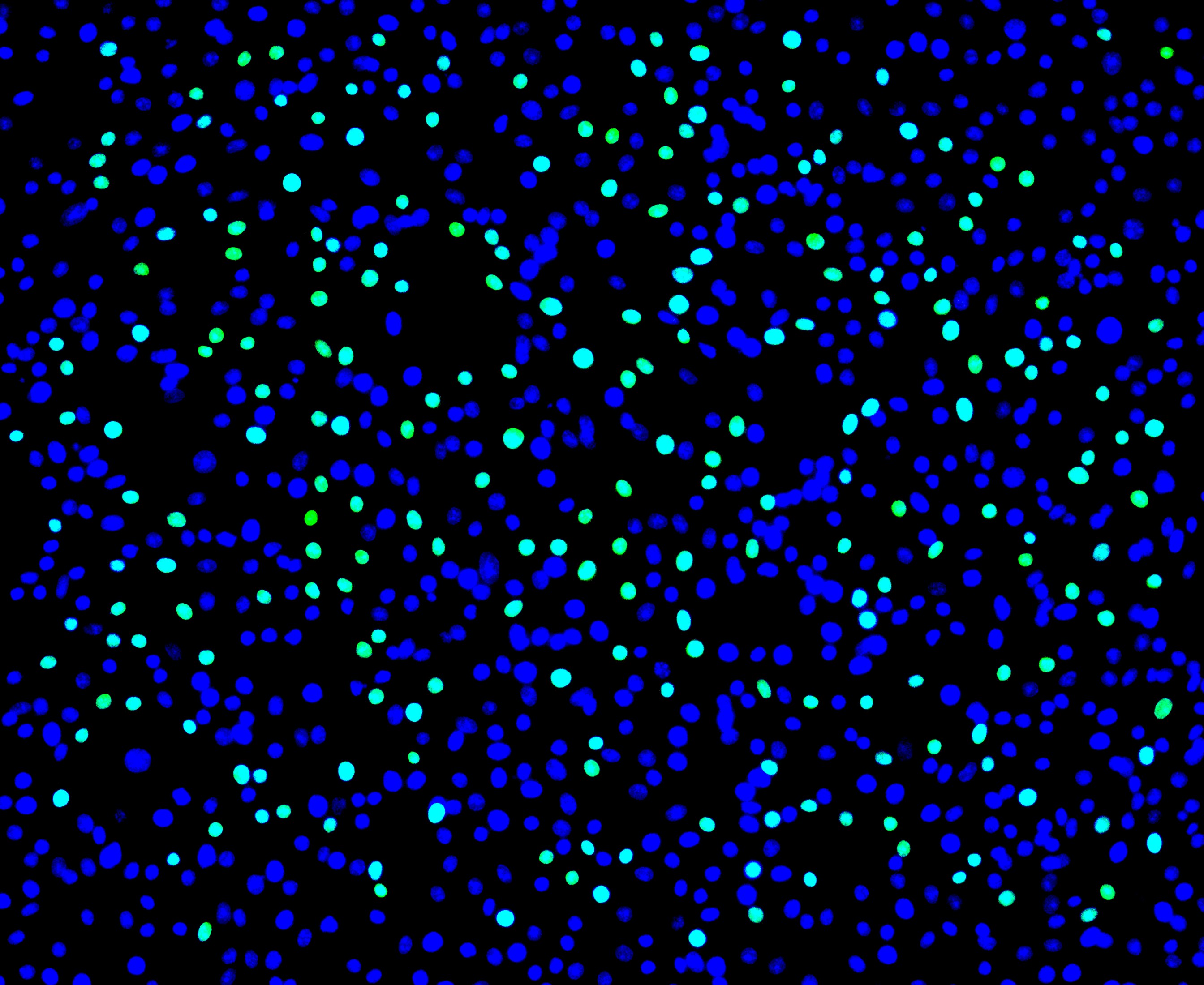

Supplement: Supplemental Material [file KBIE_A_2057632_SM9317.zip › supplementary/Fig6D_HR_Merged.tif]

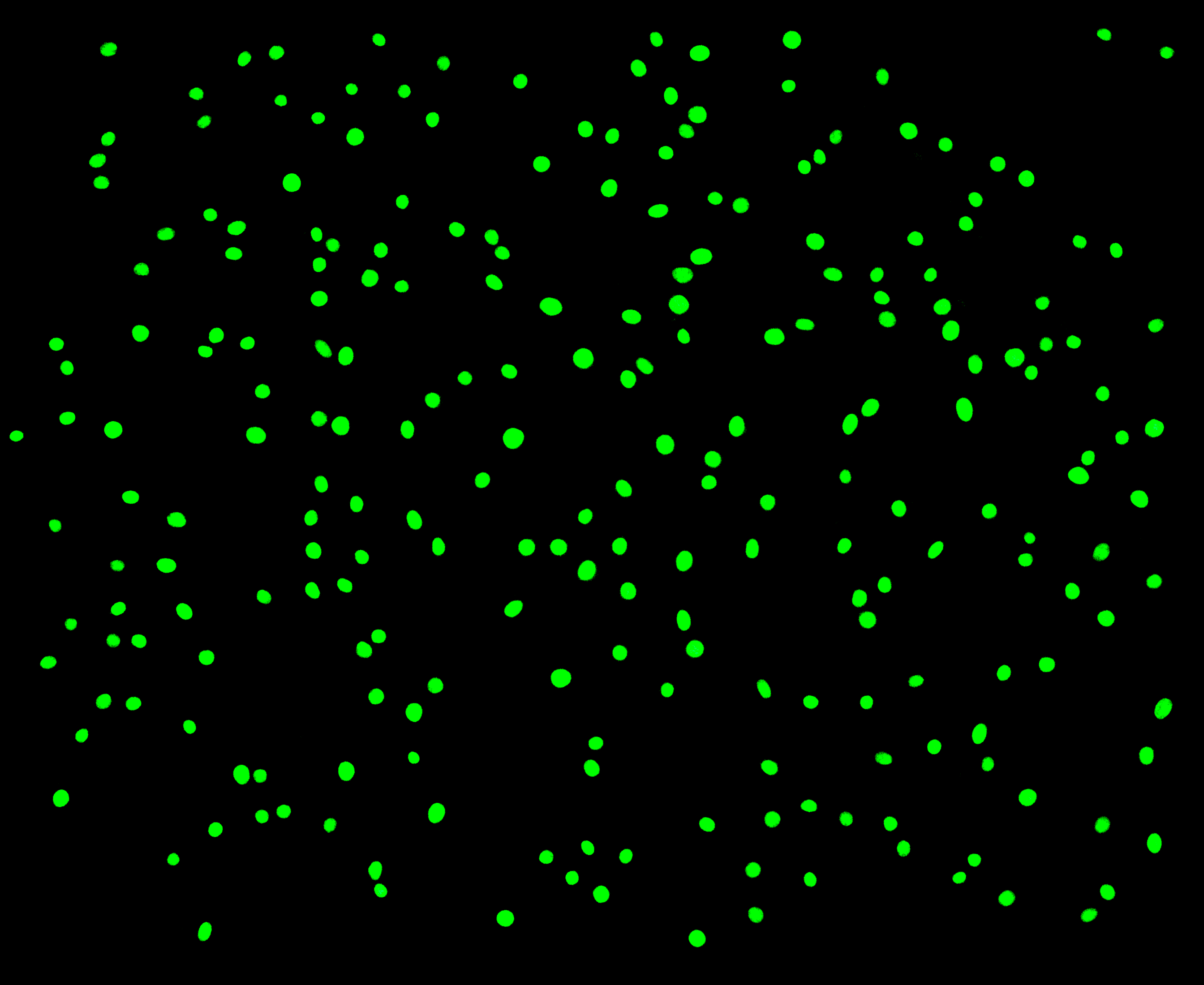

Supplement: Supplemental Material [file KBIE_A_2057632_SM9317.zip › supplementary/Fig6D_HR_Tunel.tif]

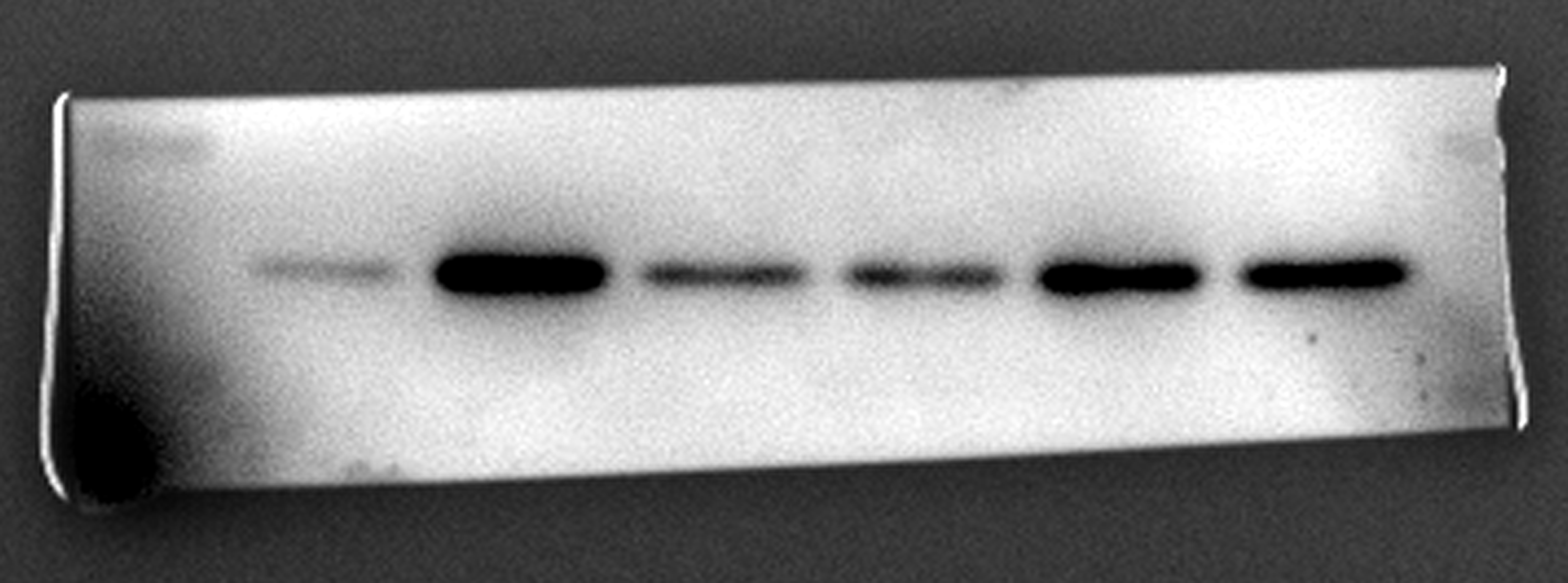

Supplement: Supplemental Material [file KBIE_A_2057632_SM9317.zip › supplementary/Fig6F_Bax.tif]

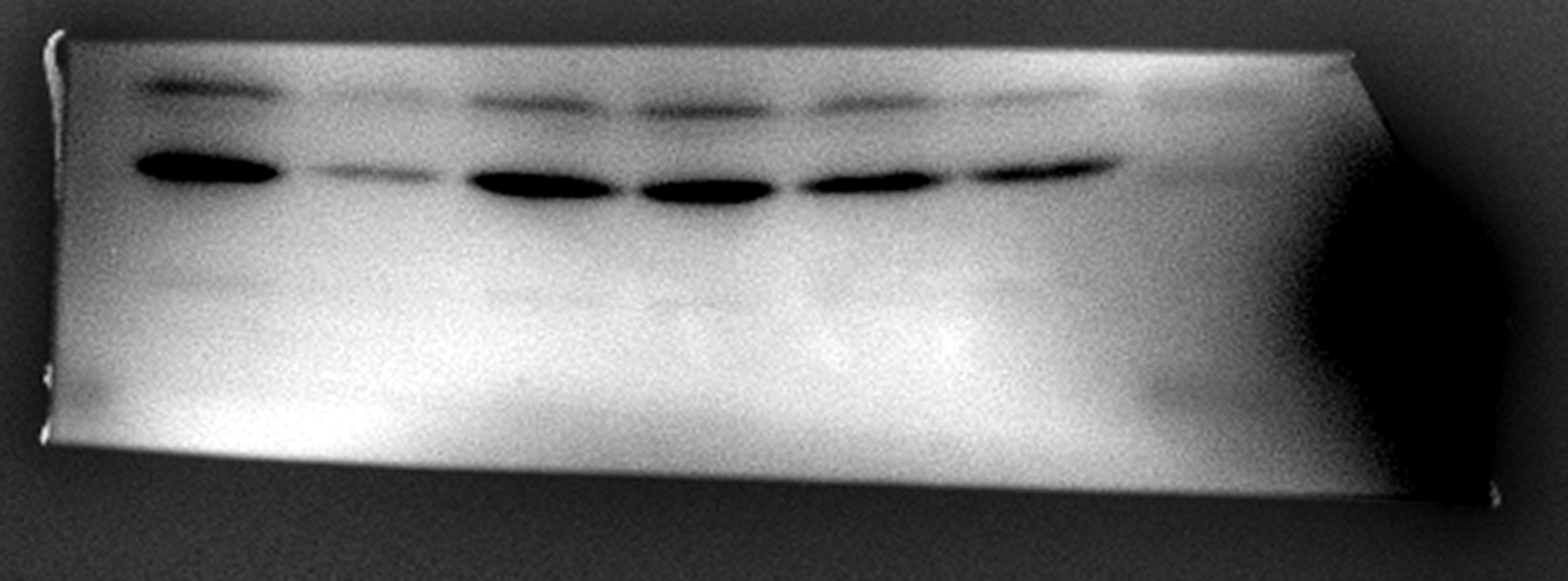

Supplement: Supplemental Material [file KBIE_A_2057632_SM9317.zip › supplementary/Fig6F_Bcl_2.tif]

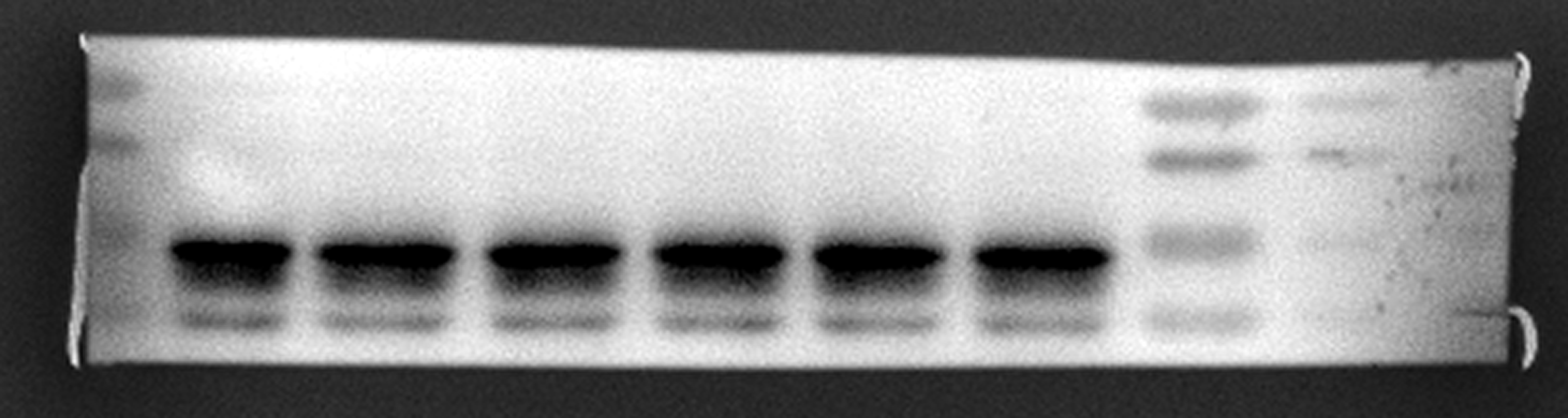

Supplement: Supplemental Material [file KBIE_A_2057632_SM9317.zip › supplementary/Fig6F_caspase3.tif]

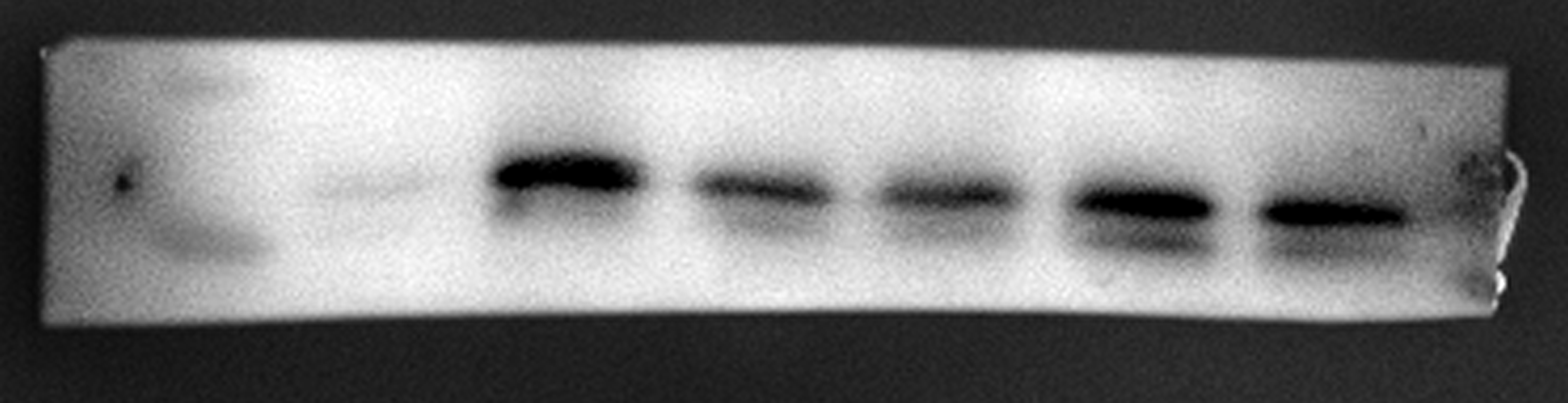

Supplement: Supplemental Material [file KBIE_A_2057632_SM9317.zip › supplementary/Fig6F_cleaved caspase3.tif]

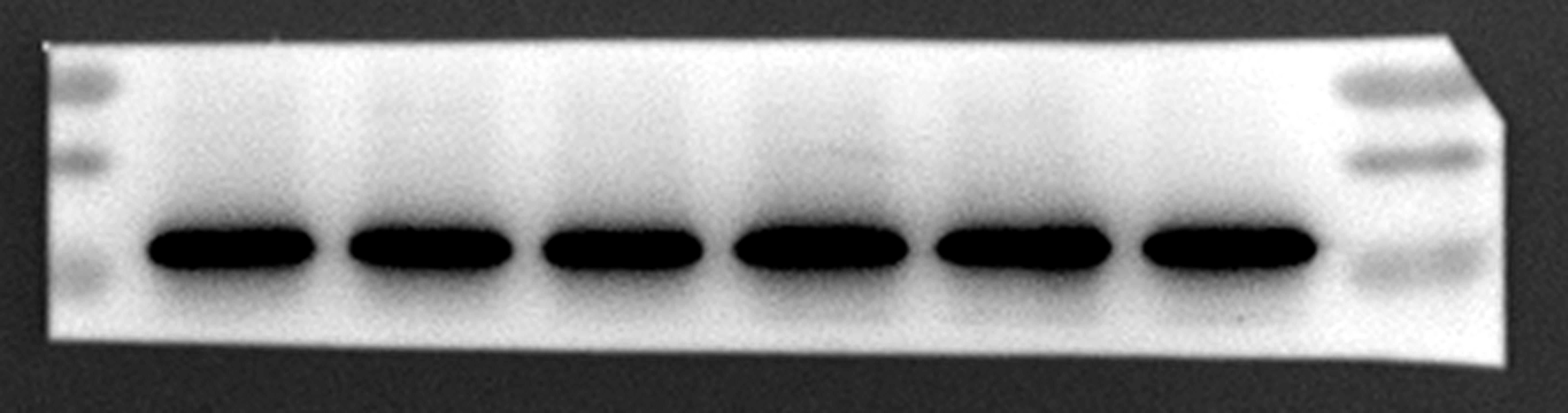

Supplement: Supplemental Material [file KBIE_A_2057632_SM9317.zip › supplementary/Fig6F_GAPDH.tif]

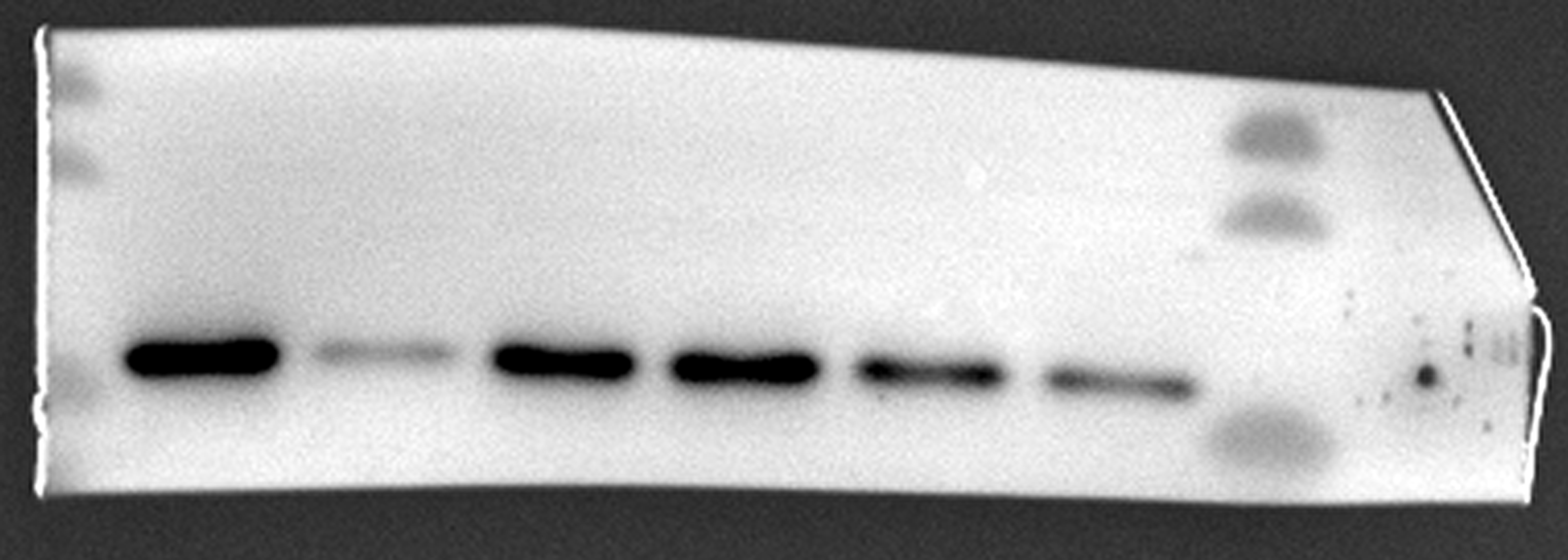

Supplement: Supplemental Material [file KBIE_A_2057632_SM9317.zip › supplementary/Fig7B_claudin_1.tif]

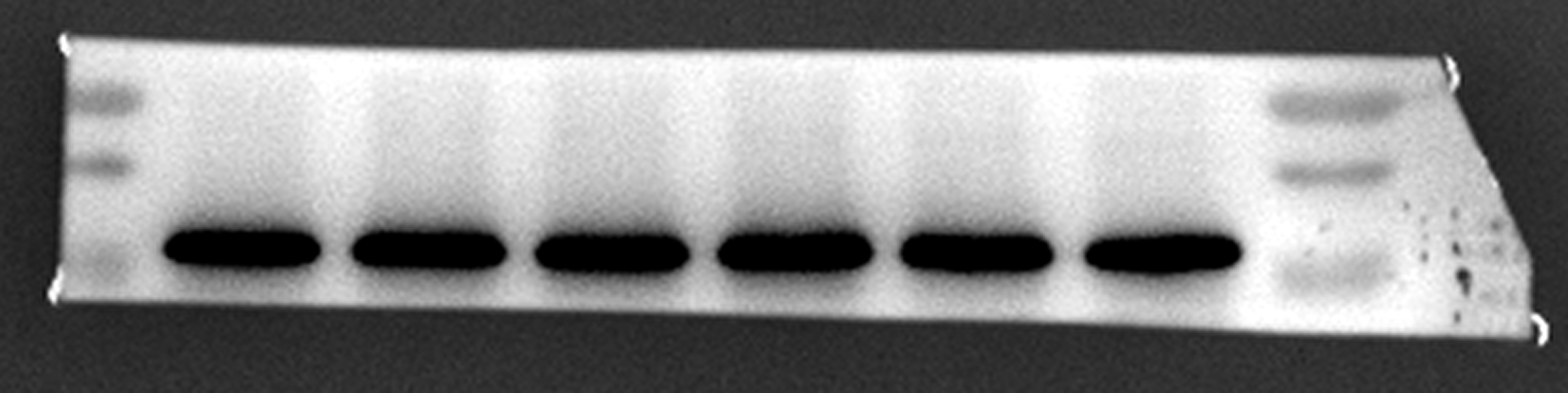

Supplement: Supplemental Material [file KBIE_A_2057632_SM9317.zip › supplementary/Fig7B_GAPDH.tif]

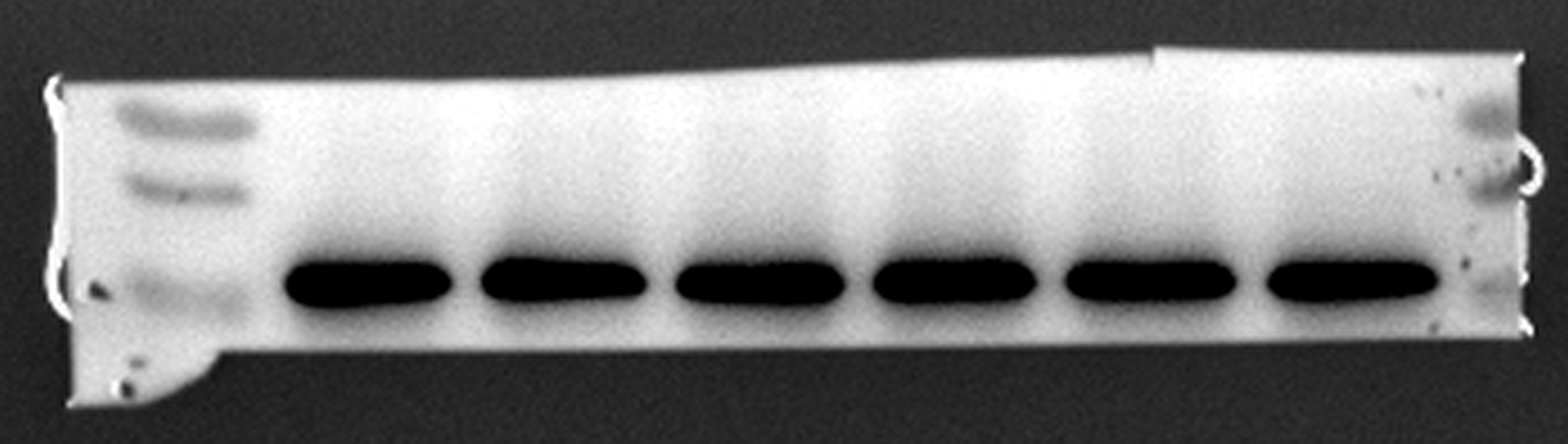

Supplement: Supplemental Material [file KBIE_A_2057632_SM9317.zip › supplementary/Fig7B_GAPDH_1.tif]

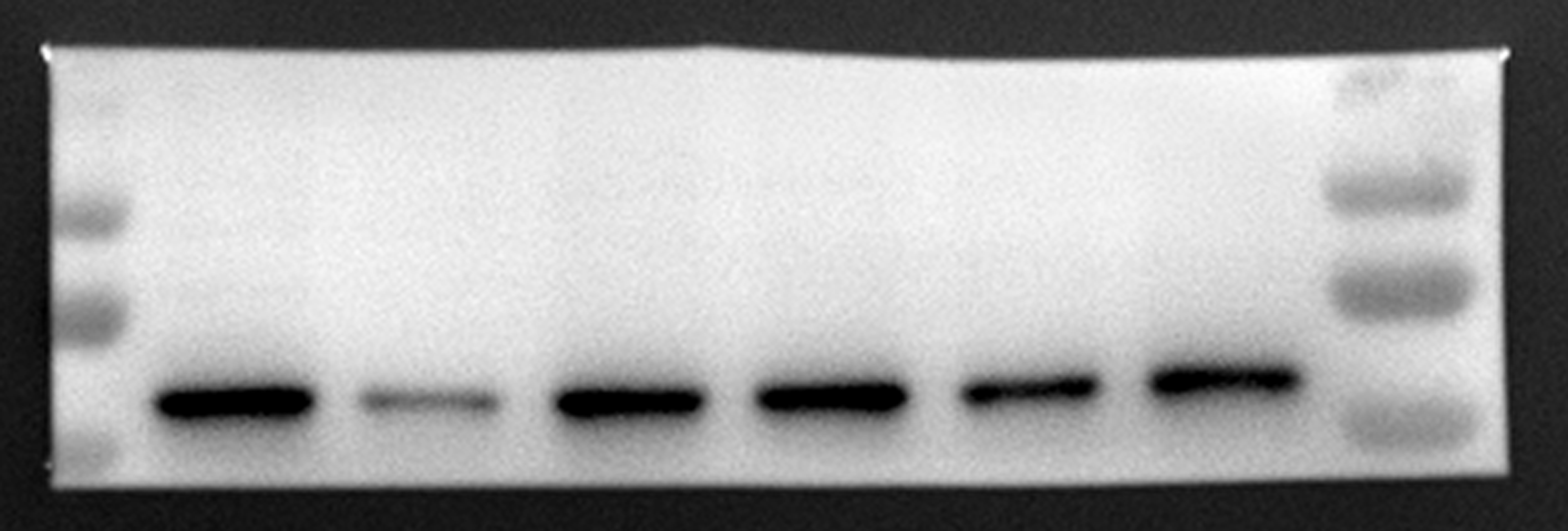

Supplement: Supplemental Material [file KBIE_A_2057632_SM9317.zip › supplementary/Fig7B_occludin.tif]

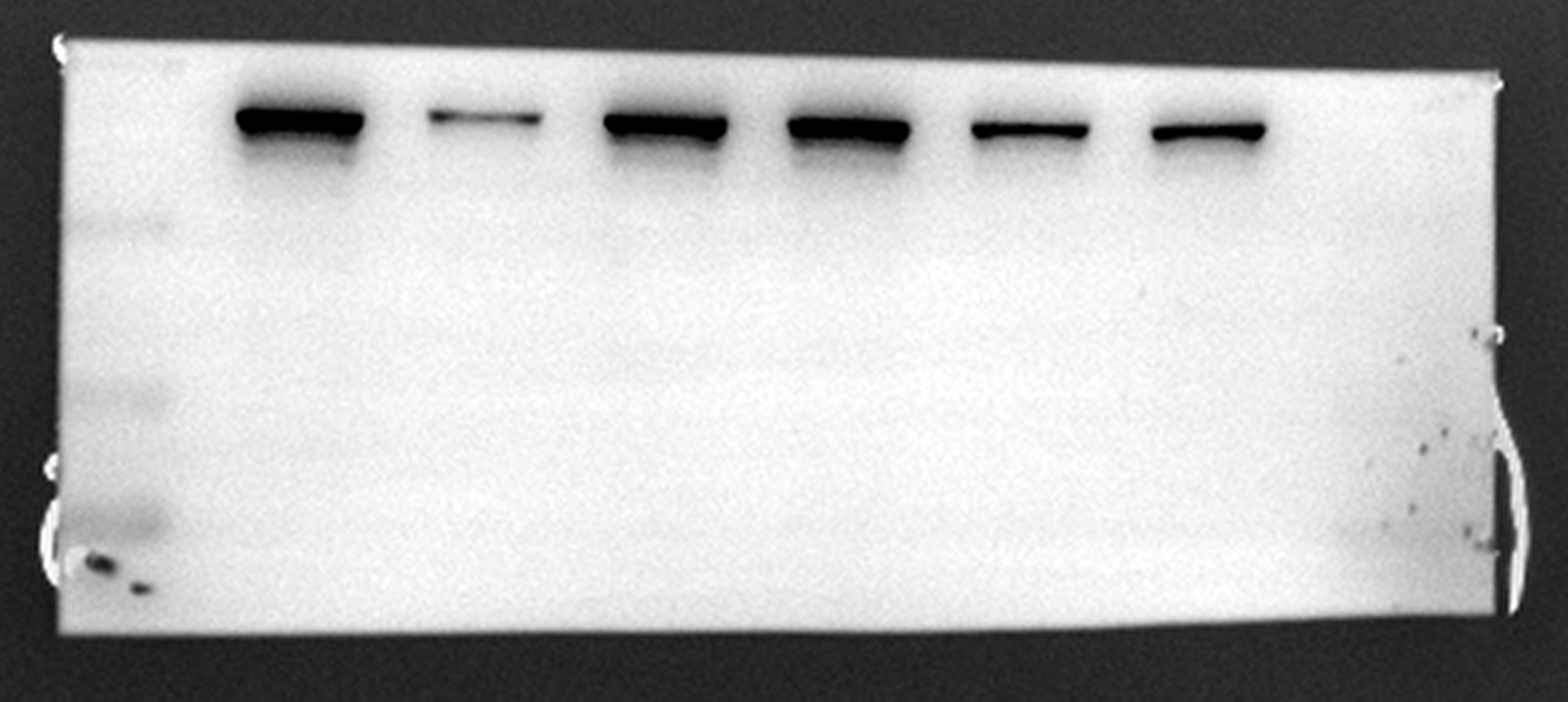

Supplement: Supplemental Material [file KBIE_A_2057632_SM9317.zip › supplementary/Fig7B_ZO_1.tif]

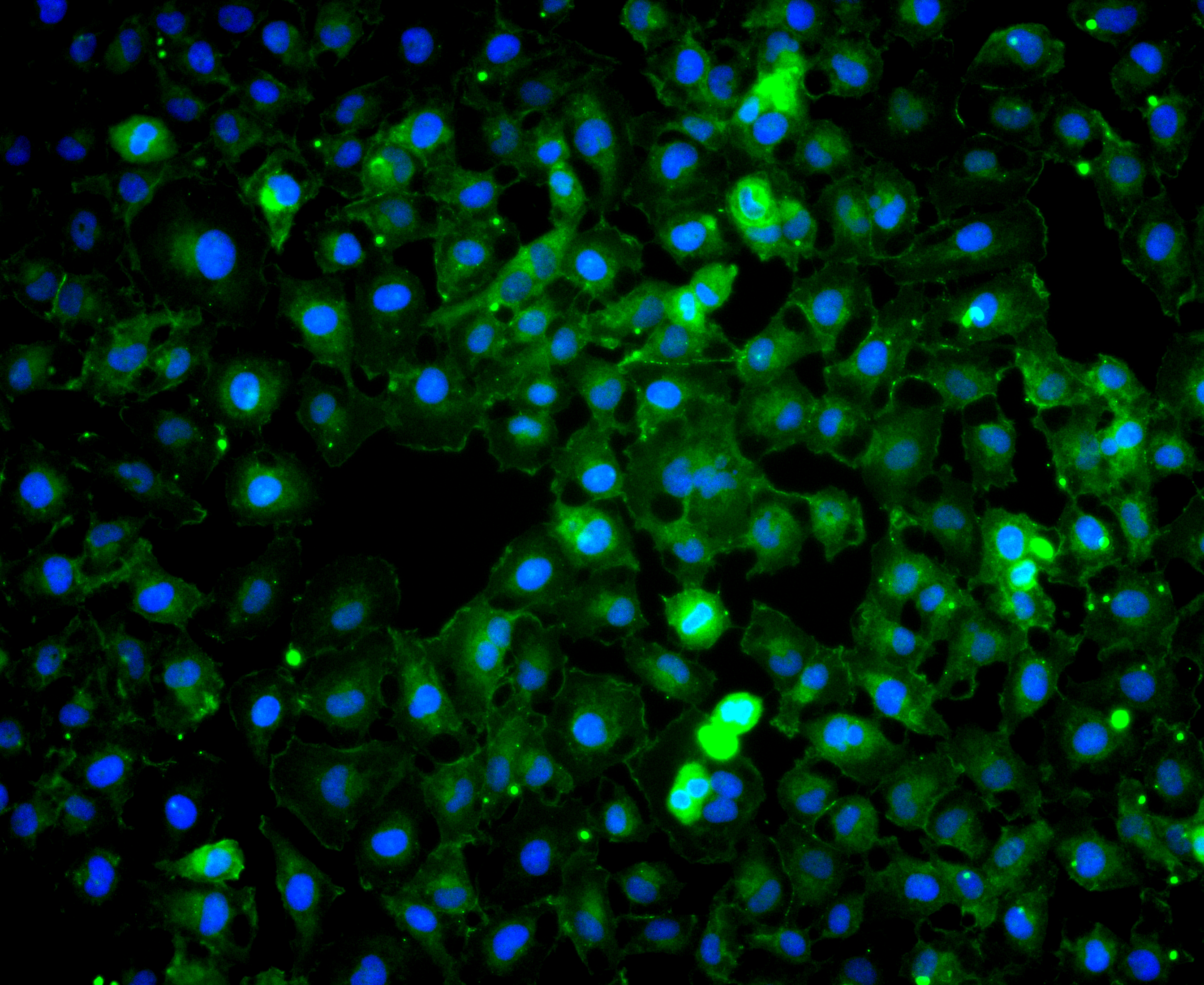

Supplement: Supplemental Material [file KBIE_A_2057632_SM9317.zip › supplementary/Fig7C_Control_Merged.tif]

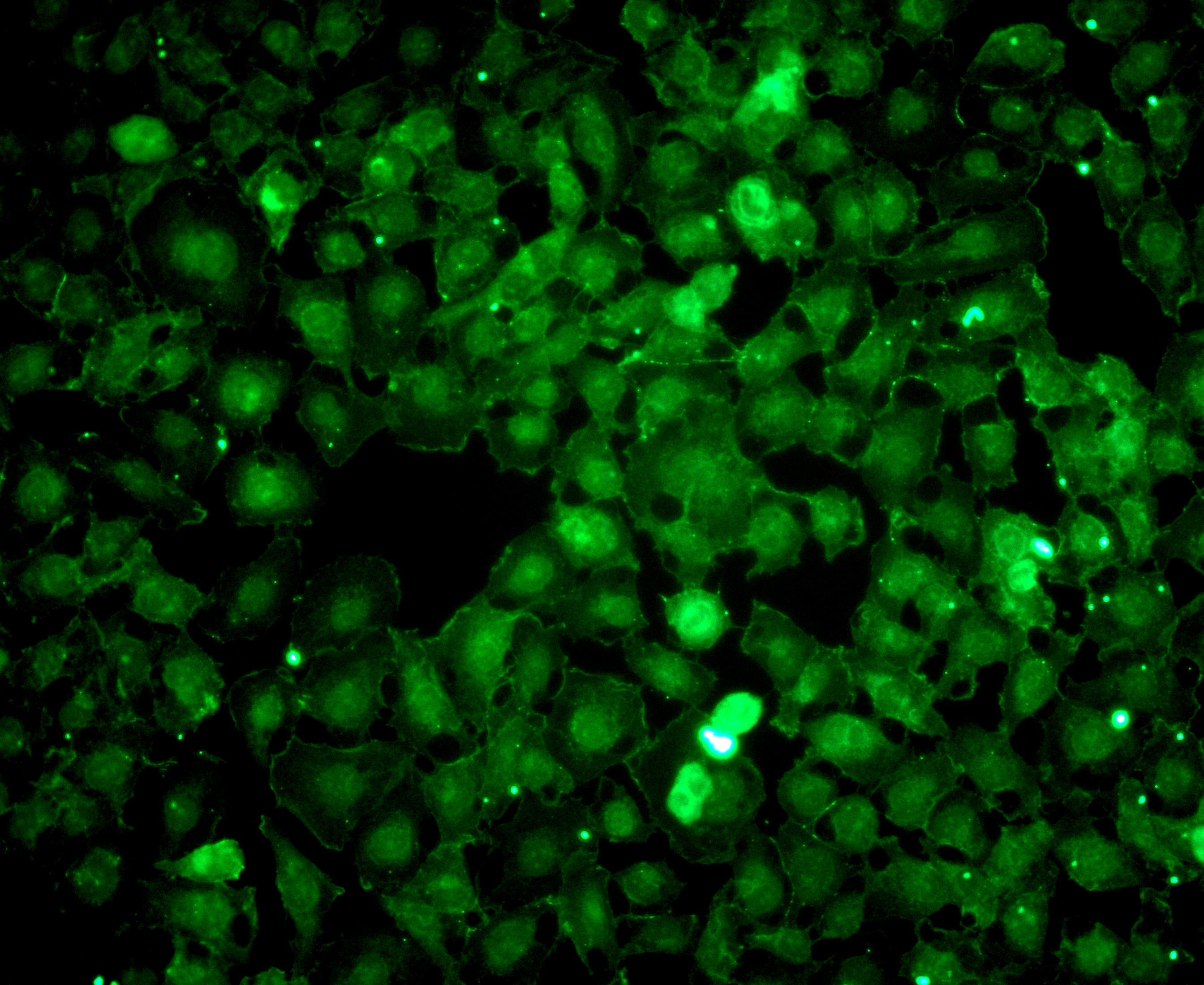

Supplement: Supplemental Material [file KBIE_A_2057632_SM9317.zip › supplementary/Fig7C_Control_ZO_1.tif]

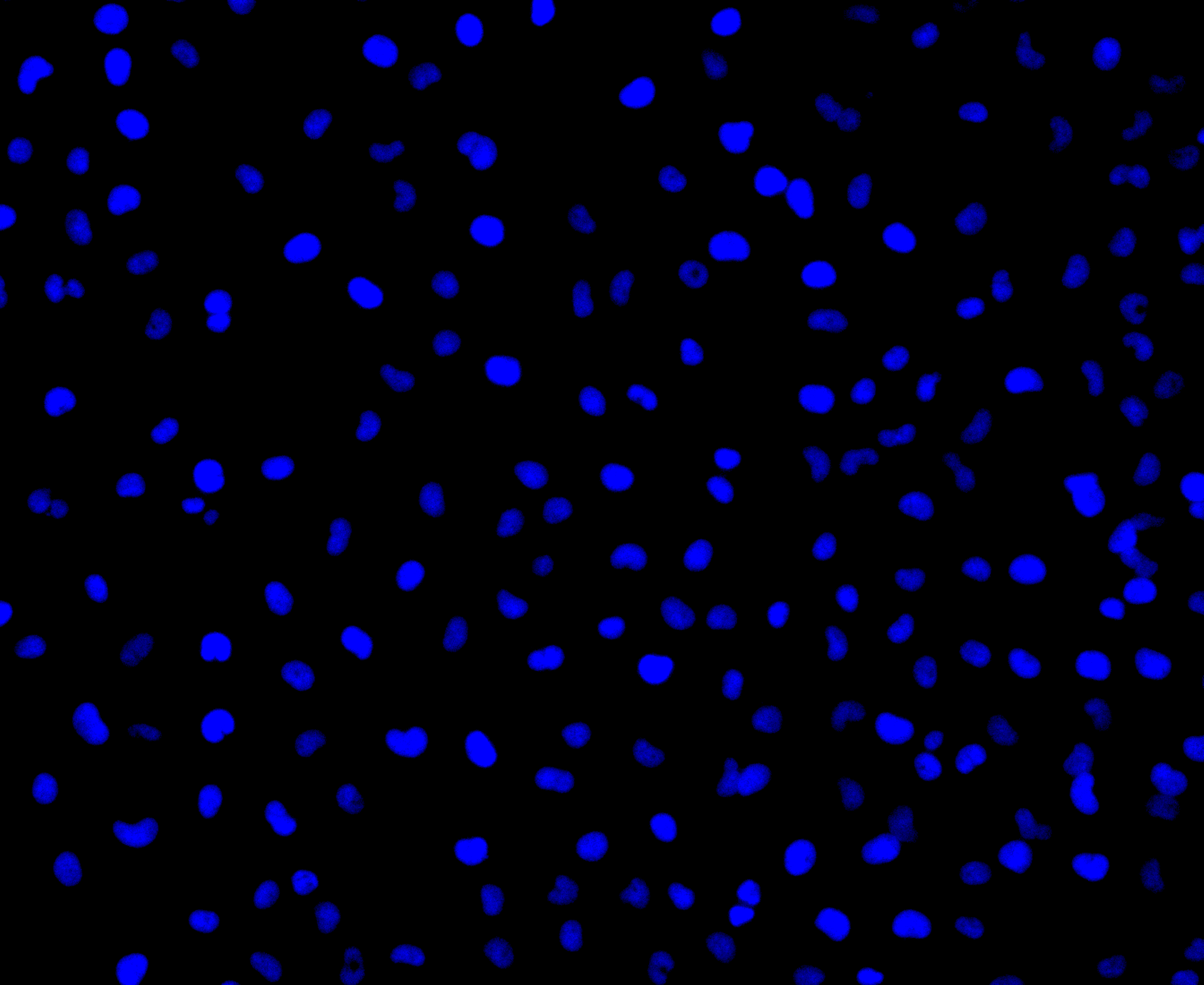

Supplement: Supplemental Material [file KBIE_A_2057632_SM9317.zip › supplementary/Fig7C_HR_1_5 ngmL_Oxycodone_BD1047_DAPI.tif]

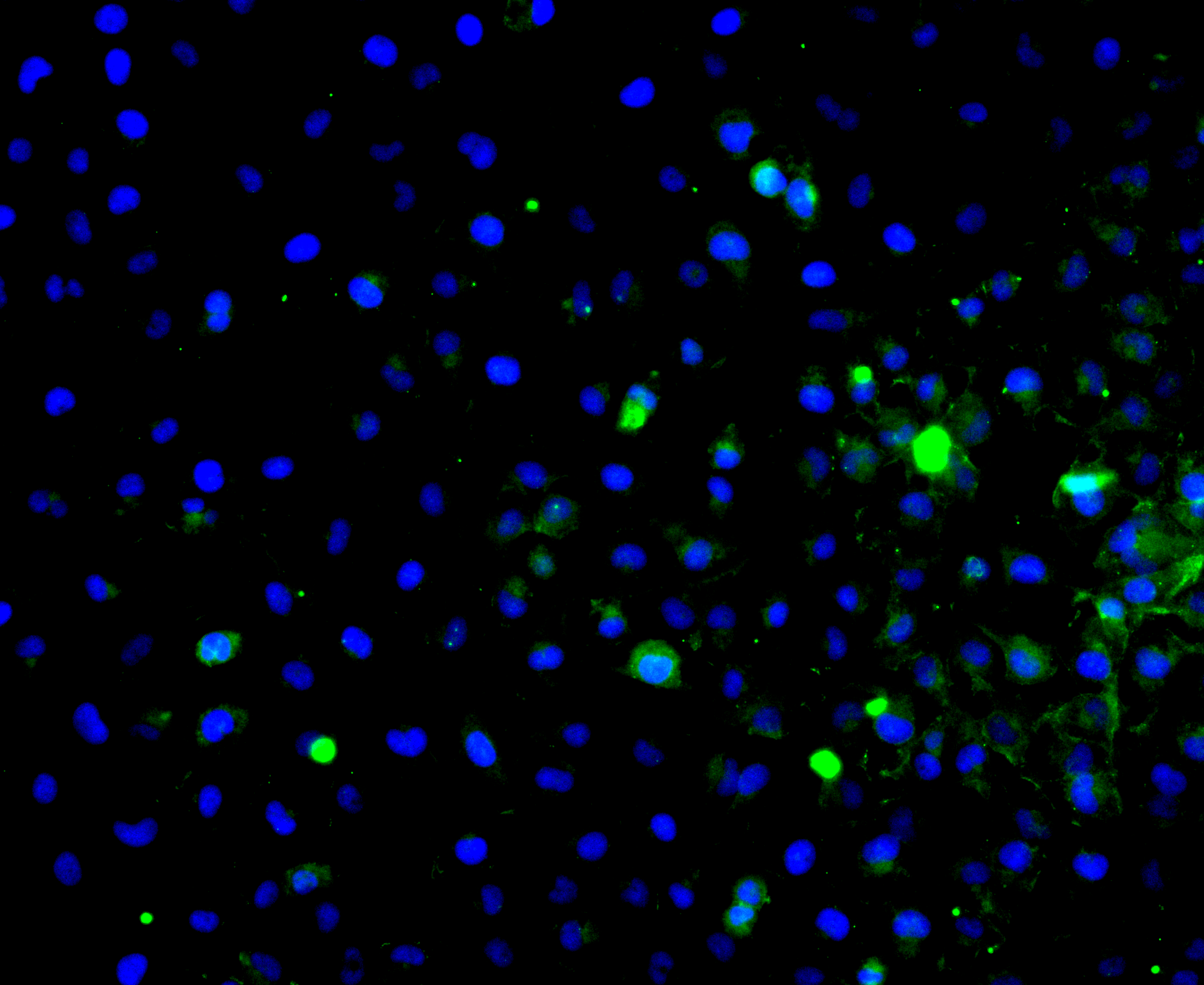

Supplement: Supplemental Material [file KBIE_A_2057632_SM9317.zip › supplementary/Fig7C_HR_1_5 ngmL_Oxycodone_BD1047_Merged.tif]

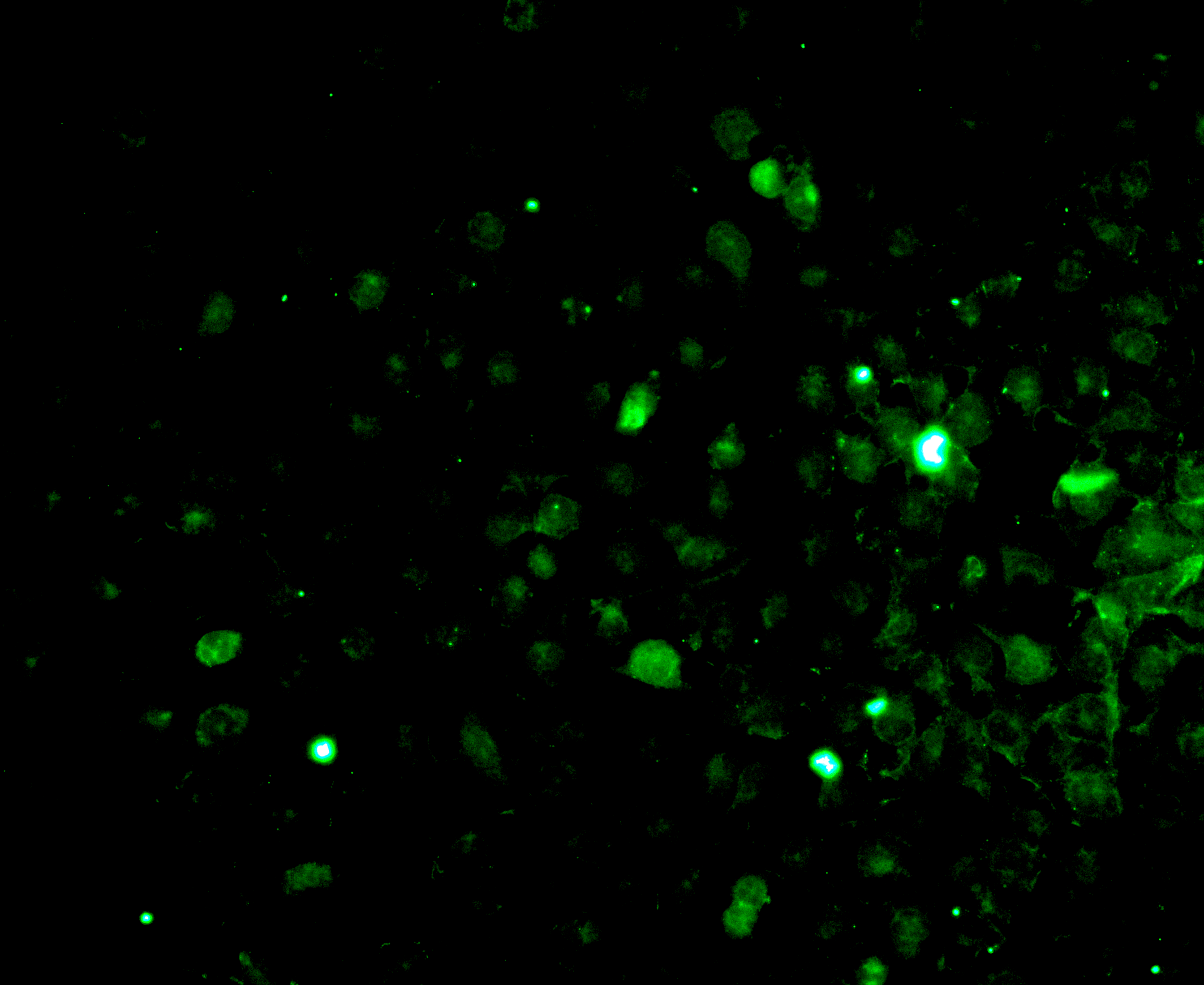

Supplement: Supplemental Material [file KBIE_A_2057632_SM9317.zip › supplementary/Fig7C_HR_1_5 ngmL_Oxycodone_BD1047_ZO_1.tif]

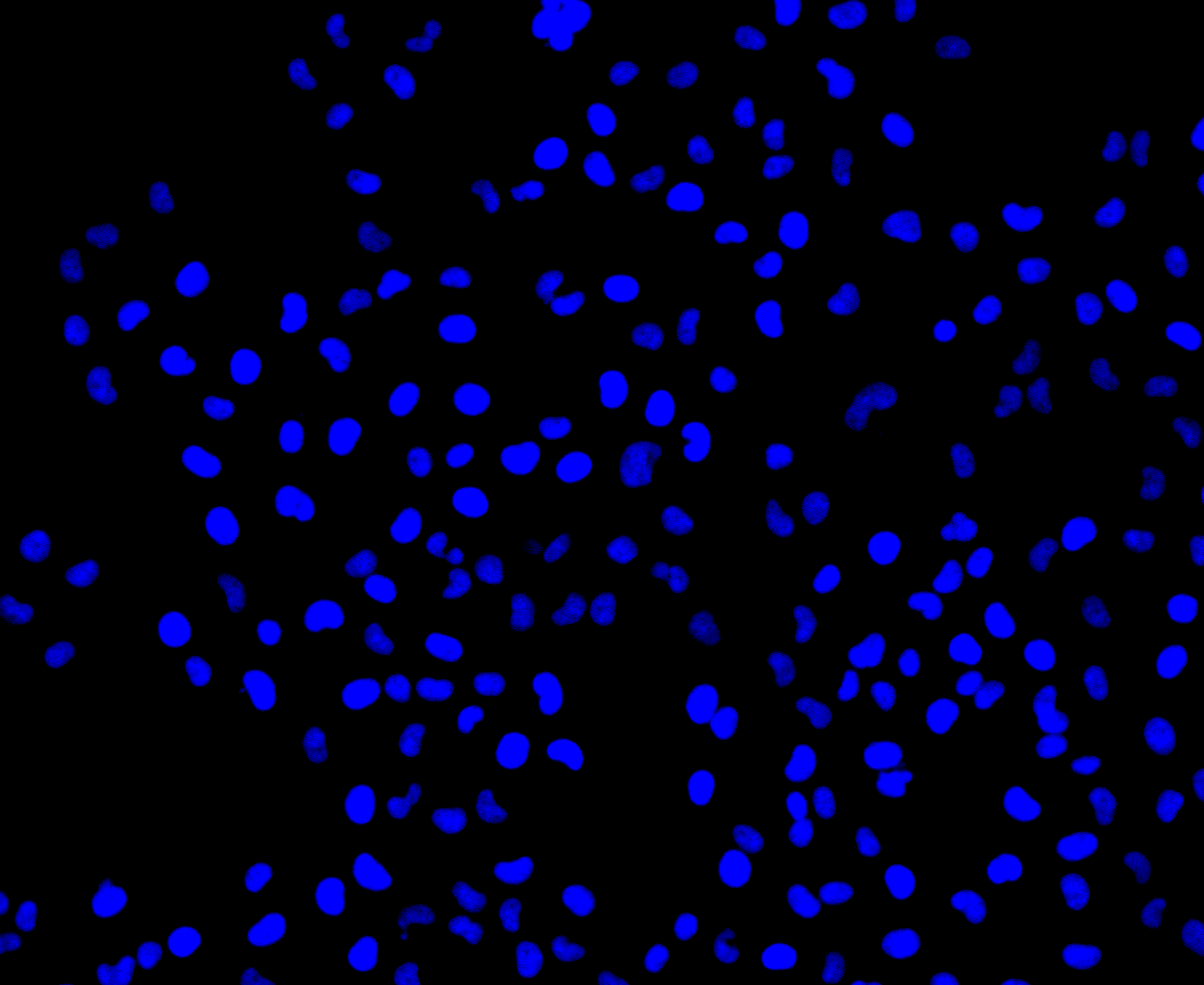

Supplement: Supplemental Material [file KBIE_A_2057632_SM9317.zip › supplementary/Fig7C_HR_1_5 ngmL_Oxycodone_DAPI.tif]

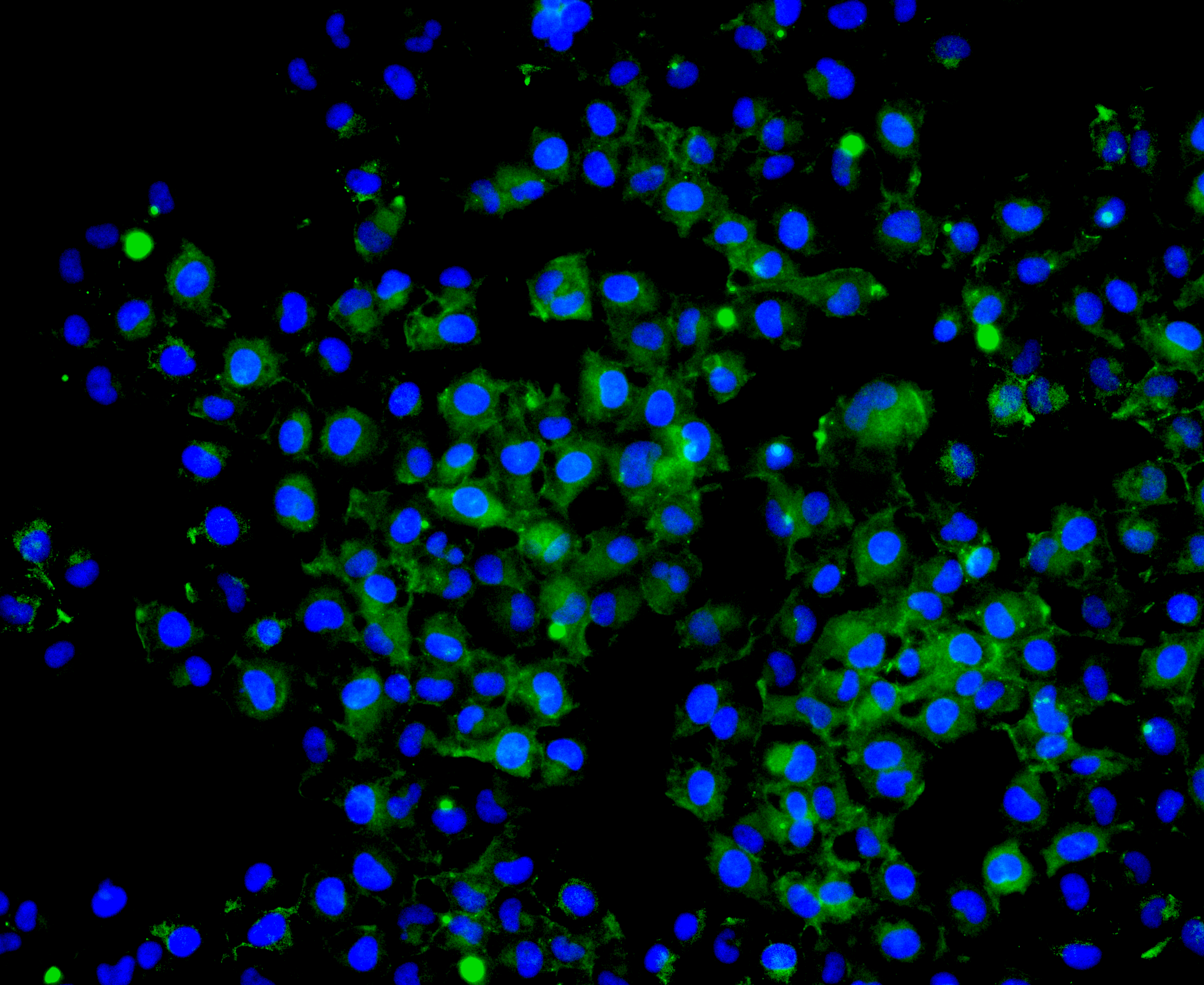

Supplement: Supplemental Material [file KBIE_A_2057632_SM9317.zip › supplementary/Fig7C_HR_1_5 ngmL_Oxycodone_Merged.tif]

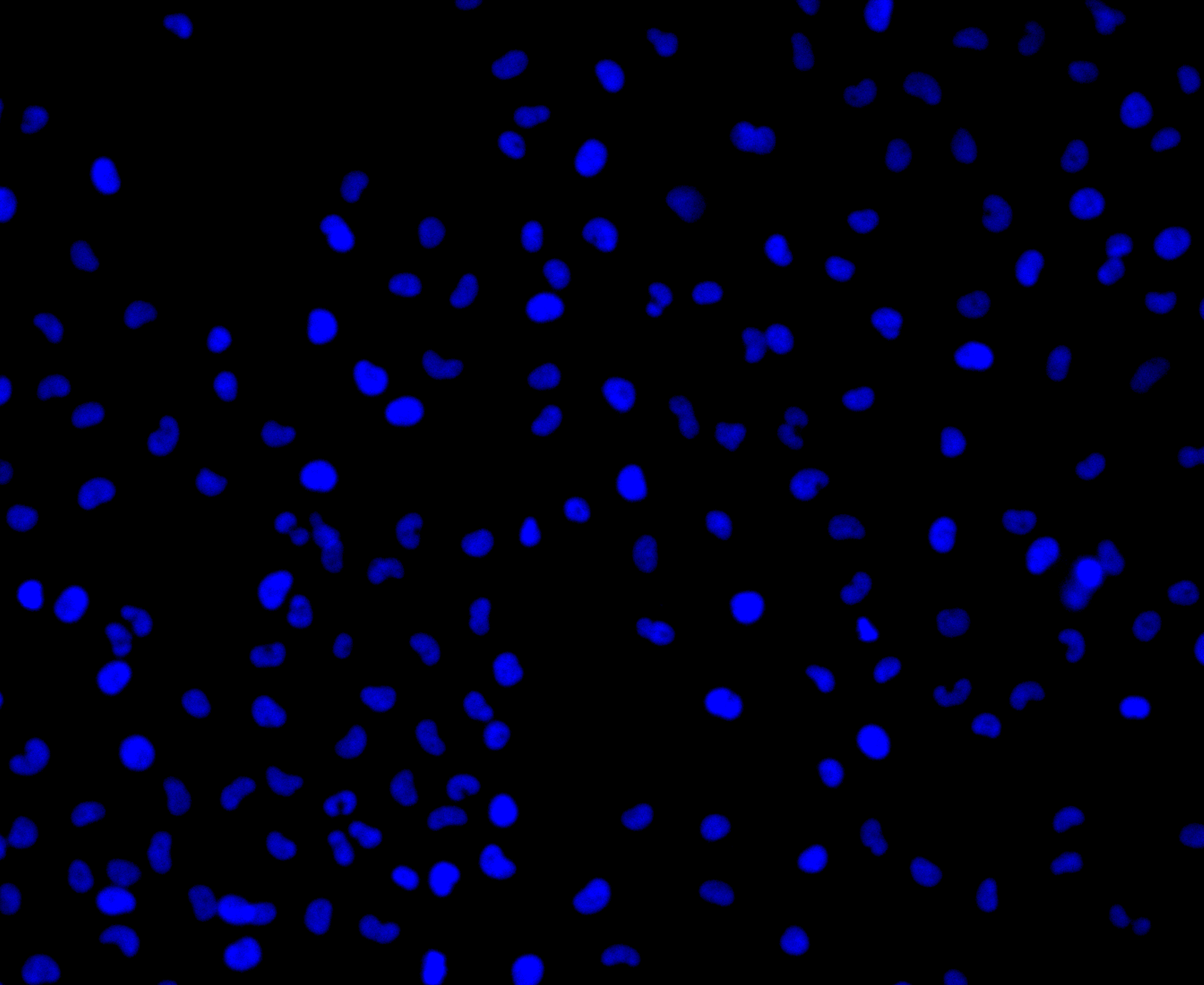

Supplement: Supplemental Material [file KBIE_A_2057632_SM9317.zip › supplementary/Fig7C_HR_1_5 ngmL_Oxycodone_shRNA_NC_DAPI.tif]

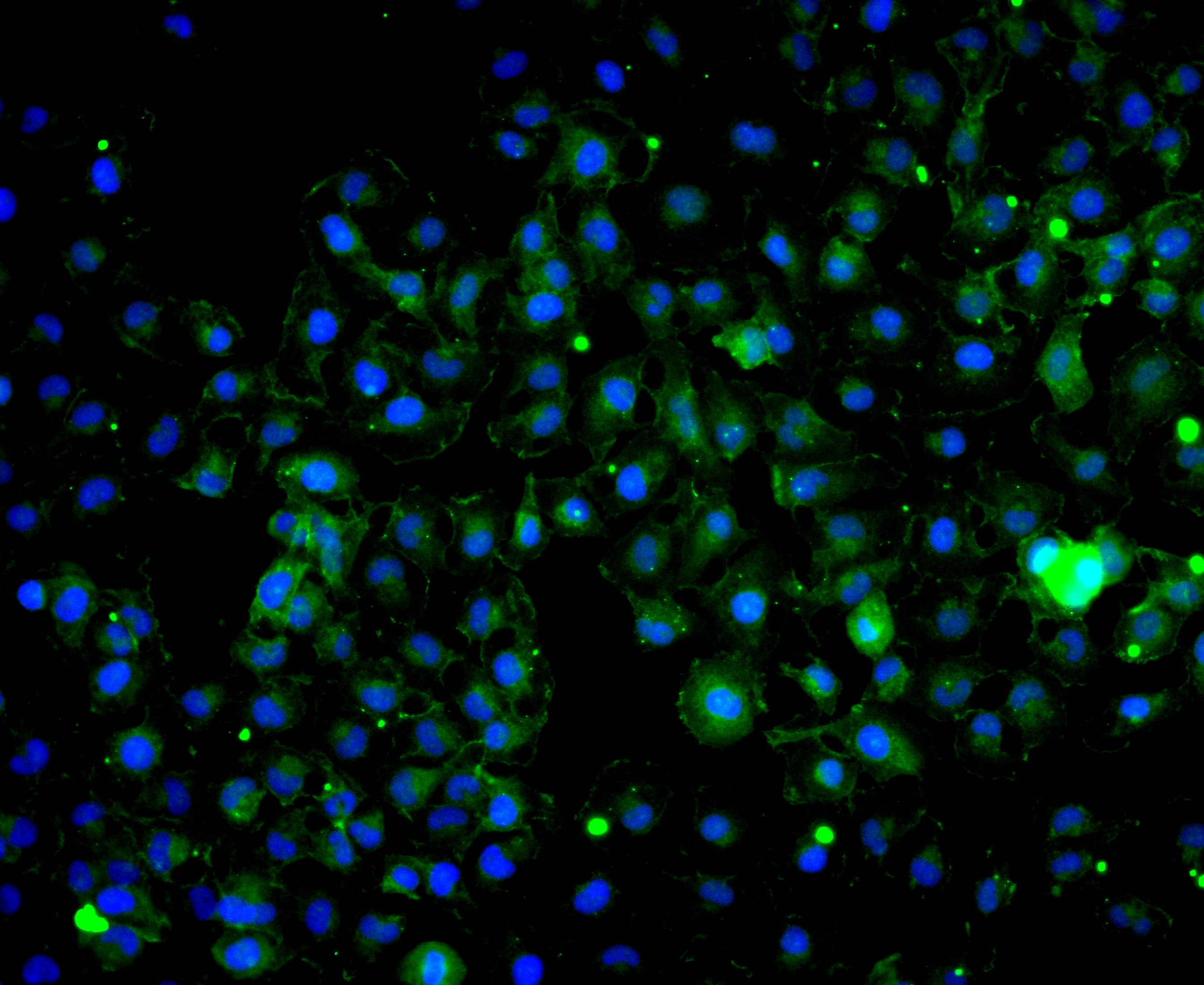

Supplement: Supplemental Material [file KBIE_A_2057632_SM9317.zip › supplementary/Fig7C_HR_1_5 ngmL_Oxycodone_shRNA_NC_Merged.tif]

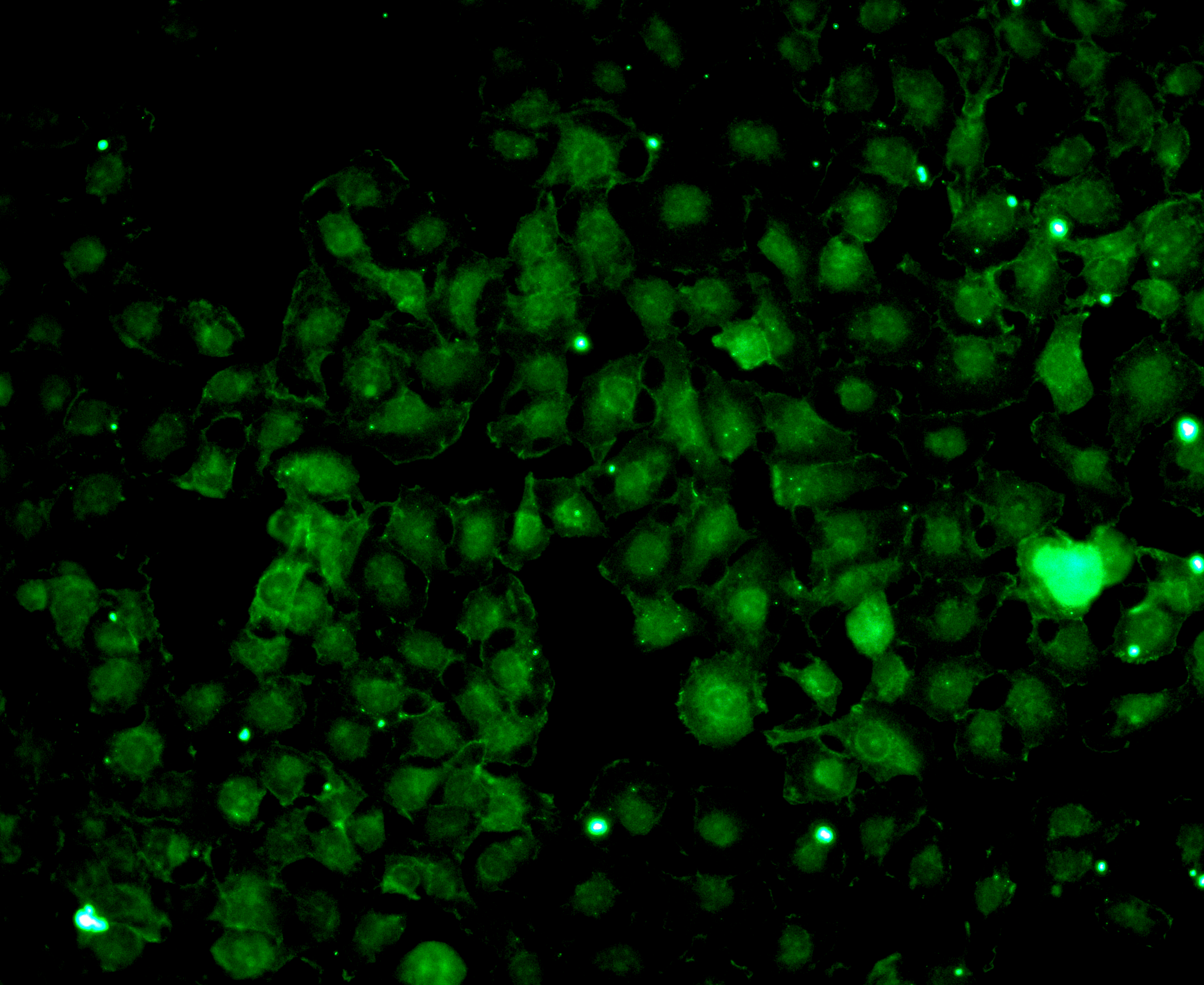

Supplement: Supplemental Material [file KBIE_A_2057632_SM9317.zip › supplementary/Fig7C_HR_1_5 ngmL_Oxycodone_shRNA_NC_ZO_1.tif]

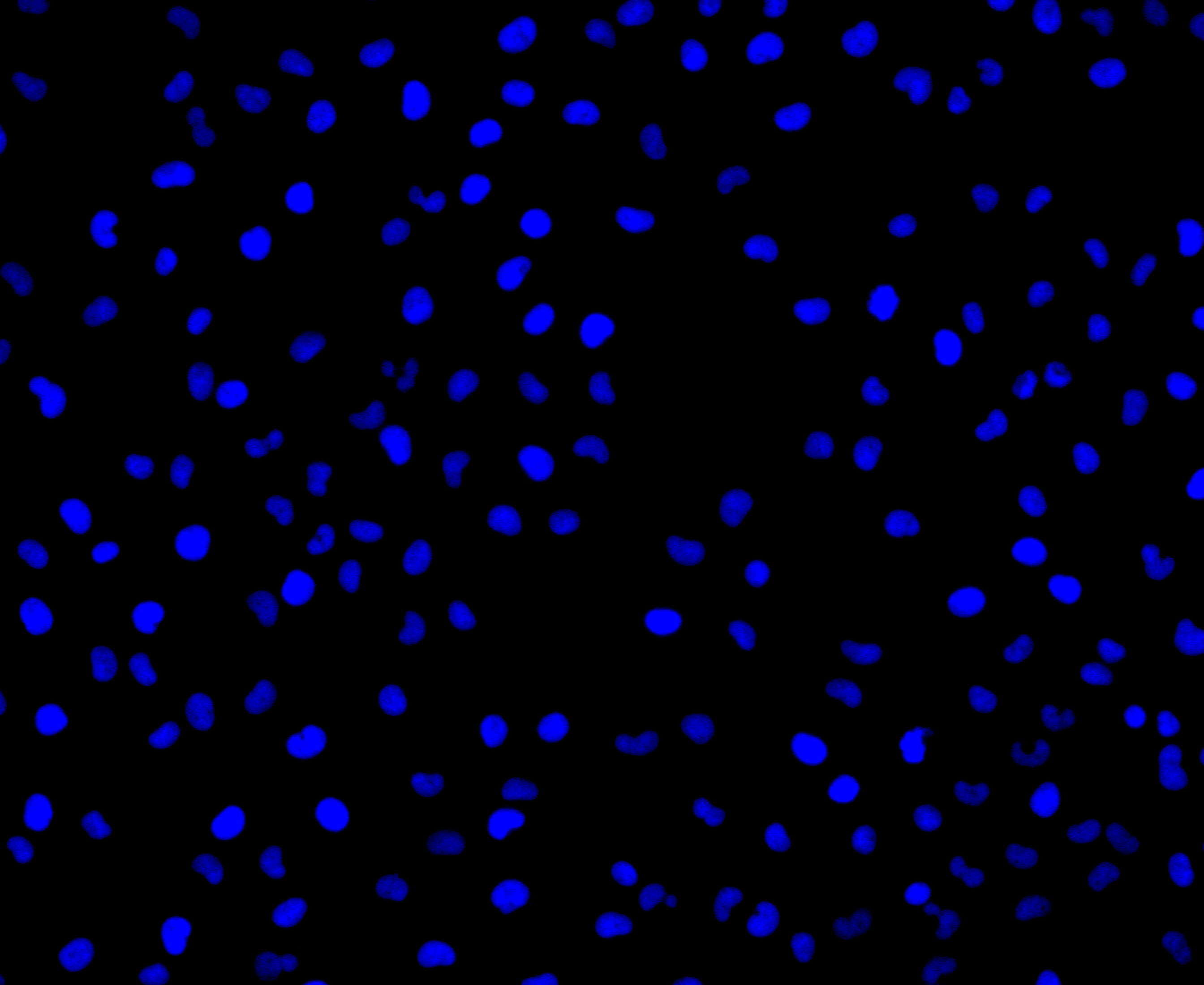

Supplement: Supplemental Material [file KBIE_A_2057632_SM9317.zip › supplementary/Fig7C_HR_1_5 ngmL_Oxycodone_shRNA_SIGMAR1_DAPI.tif]

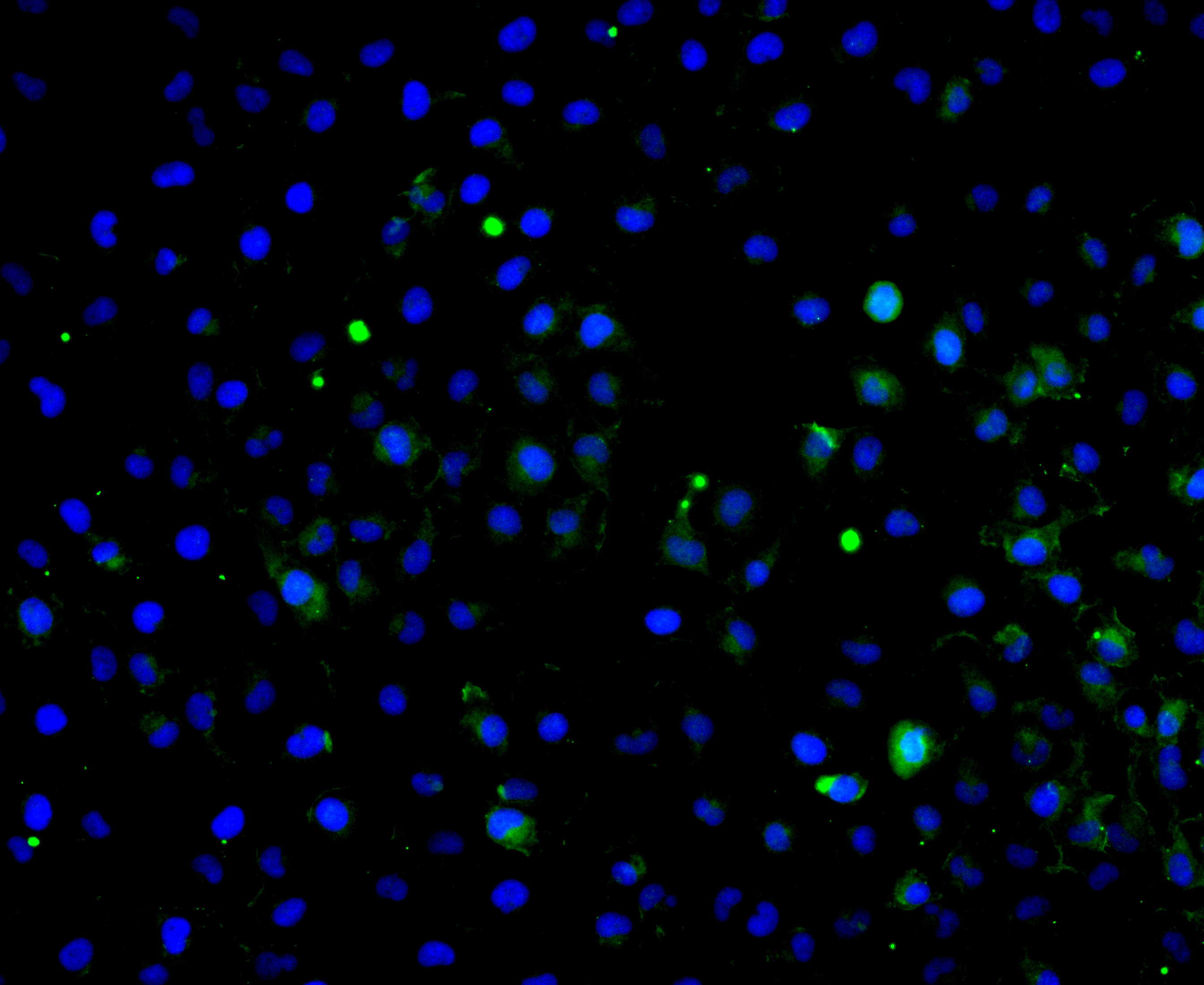

Supplement: Supplemental Material [file KBIE_A_2057632_SM9317.zip › supplementary/Fig7C_HR_1_5 ngmL_Oxycodone_shRNA_SIGMAR1_Merged.tif]
